# Training to Improve Precision and Accuracy in the Measurement of Fiber Morphology

**DOI:** 10.1371/journal.pone.0167664

**Published:** 2016-12-01

**Authors:** Nathan A. Hotaling, Jun Jeon, Mary Beth Wade, Derek Luong, Xavier-Lewis Palmer, Kapil Bharti, Carl G. Simon

**Affiliations:** 1 Biosystems & Biomaterials Division, National Institute of Standards & Technology, Gaithersburg, Maryland; 2 Unit on Ocular and Stem Cell Translational Research, National Eye Institute, National Institutes of Health, Bethesda, Maryland; 3 Integrated Biosciences PhD Student, Department of Polymer Science, The University of Akron, Akron, Ohio; 4 Department of Polymer Science, The University of Akron, Akron, Ohio; Institute of Materials Science, GERMANY

## Abstract

An estimated $7.1 billion dollars a year is spent due to irreproducibility in pre-clinical data from errors in data analysis and reporting. Therefore, developing tools to improve measurement comparability is paramount. Recently, an open source tool, DiameterJ, has been deployed for the automated analysis of scanning electron micrographs of fibrous scaffolds designed for tissue engineering applications. DiameterJ performs hundreds to thousands of scaffold fiber diameter measurements from a single micrograph within a few seconds, along with a variety of other scaffold morphological features, which enables a more rigorous and thorough assessment of scaffold properties. Herein, an online, publicly available training module is introduced for educating DiameterJ users on how to effectively analyze scanning electron micrographs of fibers and the large volume of data that a DiameterJ analysis yields. The end goal of this training was to improve user data analysis and reporting to enhance reproducibility of analysis of nanofiber scaffolds. User performance was assessed before and after training to evaluate the effectiveness of the training modules. Users were asked to use DiameterJ to analyze reference micrographs of fibers that had known diameters. The results showed that training improved the accuracy and precision of measurements of fiber diameter in scanning electron micrographs. Training also improved the precision of measurements of pore area, porosity, intersection density, and characteristic fiber length between fiber intersections. These results demonstrate that the DiameterJ training module improves precision and accuracy in fiber morphology measurements, which will lead to enhanced data comparability.

## 1.1 Introduction

Recently, considerable concern about the inability of researchers to reproduce biomedical research findings has been brought to the forefront of the field [1–8]. Diverse research fields have been highlighted where reproducibility of data is low including psychology[9], stem cell biology[2,10], drug therapy[5,6,11], and cancer biology[12,13]. It has been reported by industry that drug-lead findings published in high-impact journals are reproducible in as little as 11%[5] of the cases. A recent survey of pharmaceutical companies conducted by the Alliance for Regenerative Medicine found that “product consistency and lack of standards" is the greatest challenge facing the field of regenerative medicine.[2,4]]. As such, tools to improve product consistency and to assess product quality are paramount for the advancement of clinical research.

Fiber-based scaffolds (nanofibers, microfibers) are a promising biomaterial for therapeutic use. In tissue engineering they have been used to help direct cell morphology[14–16], phenotype[17,18], and differentiation[19,20]. However, nanofiber scaffolds are generally heterogeneous in both fiber diameter and fiber orientation when electrospun. This makes assessment of the nanofiber scaffold structure and morphology difficult to do in a reproducible way. Thus, there is interest to obtain automated characterization tools of these scaffolds that can quickly and accurately assess fiber properties to enhance product consistency [21,22]. Due to the nanometer scale of fiber diameter, traditional light/fluorescent microscopes are unable to accurately assess nanofiber morphology and thus scanning electron microscopes (SEM) are typically used to assess structure. From SEM micrographs the most prevalent measurement made to assess nanofiber morphology is the fiber diameter.[23] Currently, manual measurement of fiber diameter from fiber SEM images is done using line tools in image analysis programs such as ImageJ/FIJI [24,25]. Recently, a publication comparing software tools and manual measurements on images of nanofibers showed that hundreds to thousands of nanofiber measures were required to converge on a consistent result. [22]

To help improve the process of analyzing micrographs for assessing fiber morphology, several algorithms have been published and recently, DiameterJ, an open access tool for assessing fiber morphology via a plugin for ImageJ, was developed [23,26]. DiameterJ enables automated analysis of scanning electron micrographs to rapidly determine fiber diameter, pore size, porosity, fiber intersection density and the characteristic lengths of fibers between intersections. Since its release, there have been many requests from the community (more than 15 institutions including industry, academia and public sector) for guidance on how to use DiameterJ for assessing fibrous scaffold morphology. In working with users it has also become clear that there is wide variability in how DiameterJ is being used to analyze fiber micrographs and how users are analyzing the large amount of data that DiameterJ produces. Methodological variance in data analysis can impair the comparability of work done in different laboratories [27]. Thus, we have developed a free DiameterJ training module that has been deployed on the web. The module includes 8 training documents, many interactive web pages, quizzes and tests where the participants download and analyze SEM images of fibers with verified fiber diameter values. Participant performance in the analysis of the test images has been tracked and demonstrates that the precision and accuracy of their reported fiber morphology metrics improves after completion of the training. To the Authors knowledge, no publication has developed and validated a training methodology designed to improve accuracy and reproducibility of the analysis of nanofiber morphological features and this study represents a first of its kind in the field.

## 1.2 Materials and Methods

All the raw data used to create the plots in the manuscript have been included in S1 File for the reader to analyze.

### 1.2.1 Steel Wire Samples

Scanning electron micrographs (SEM) of steel wire of known diameters were used for training. Small gauge (48 and 50 gauge) steel wire was chosen as an ideal material for training because 1) it has a highly uniform diameter and 2) because the diameter is large enough that it can be verified by other measurements [23]. Specific sizes of wire were chosen because they were commercially available and because they were small enough to be imaged using the “high magnification” settings within the SEM yet large enough to be sized by alternative means (calipers, light microscopy, impedance). To prepare the training images, two gauges of steel wire (316 stainless steel wire, HSM Wire, Inc.), diameters of 31.1 ± 0.1 μm (48 gauge) and 25.6 ± 0.1 μm (50 gauge) were obtained. The diameters of the wires were verified by light microscopy, calipers and resistance measurements as described in Hotaling et al. [23]. A sample for imaging was prepared by entangling a meter of each wire gauge by folding them in half and rolling them between gloved-hands in opposing circular motions. To remove dust, the entangled samples were washed with successive immersion (5 mL) and vortexing (5 min) in each of the following solvents: dichloromethane, dimethyl sulfoxide, tetrahydrofuran and hexafluoroisopropanol (HFIP) (Sigma-Aldrich).

### 1.2.2 SEM Microscopy

SEM imaging was performed according to the guidelines provided by Goldstein et al. [28]. This current work focuses on the robustness of the image analysis and so does not address variability in scanning electron microscopy imaging technique [28,29]. Steel wire samples (two gauge sample) were mounted onto SEM stubs using carbon tape (Ted Pella, Inc.), placed under house vacuum overnight with desiccant and gold sputter-coated (120 s at 75 mA, Desk V HP, Denton Vacuum, approximately 10 nm of gold deposition). Twelve scanning electron micrographs were captured of each sample (Hitachi S4700 SEM, 5kV, 10mA, ≈13 mm working distance, magnification 300X).

### 1.2.3 Participant Composition and Consent

No personal information was collected from participants. By checking a box all participants indicated that they gave their informed consent that their anonymized performance data would be publicly disseminated and published. If participants checked this box the record of their consent and the subsequent participant response to questions given in the training were recorded. If participants did not check this box, no data was recorded. All participants whose data is shown in this study consented to the use of their anonymized data for public dissemination and publication. The Human Subjects Protection Office (HSPO) of NIST declared the research exempt from IRB review in document MML-16-0025. Prior to taking the training all participants affirmed that they had proficiency in common spreadsheet software. At the time of this writing, all participants who had completed the training and consented, were included in the analysis.

### 1.2.4 Training Overview

**Fig 1:**
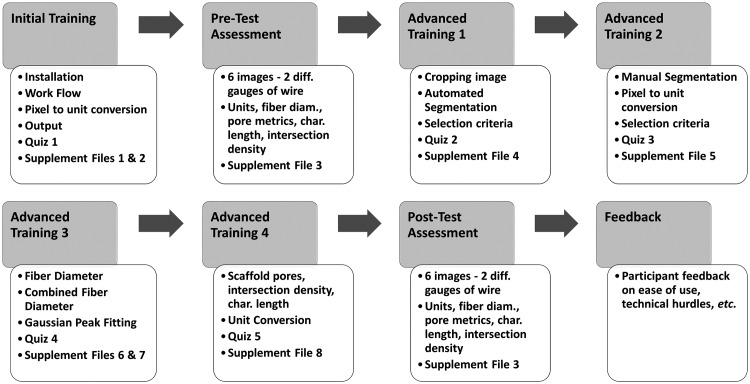
General overview of training process for each participant.

The entire training module, including all documents, quizzes and images are freely available at https://sites.google.com/site/diameterj/. This training is not a formal designation and does not represent a certification by NIST or the authors of this manuscript. Upon accessing the training module, participants are assigned a randomly generated identification number that enables progress to be tracked. An overview of the entire training module is in Fig 1. The module tracked participant progress so that they were able to stop or start training, as fit their schedule. Prior to the Pre-Test participants were given installation and user instructions (S2 File) as well as an introduction to the software (S3 File) to understand how it works and what DiameterJ outputs.

### 1.2.5 Pre-Test Assessment

For the Pre-Test, six SEM micrographs of steel wire were given to each participant and they were asked to analyze the images with DiameterJ. Participant output for the Pre-Test was collected using the form provided in S4 File. Participants were asked for fiber diameter, pore area, porosity, intersection density and length between intersections.

### 1.2.6 Advanced Training 1 to 4

Upon completing the Pre-Test, participants were given a four-step advanced training series (Fig 1). Each step used a combination of interactive web pages and written documentation to cover distinct aspects of the analysis process including: image cropping and segmentation (S5 File), manual segmentation and pixel to unit conversion (S6 File), fiber diameter analysis (S7 File), combined fiber analysis (S8 File) and fiber metric analysis (S9 File). After each step, participants were given quizzes (Quizzes 2 to 5) to ensure that they mastered the concepts covered in each document. Upon completion of Quiz 5, participants were considered “fully trained.”

### 1.2.7 Post-Test Assessment

Once fully trained, participants took a Post-Test in which they analyzed a new set of 6 images of the same steel wire sample that was analyzed in the Pre-Test. Participant analysis of these images was collected via the same document as the “Pre-Test” (S4 File) so that no variability was introduced from differences in question wording. Users reported that the training takes between 3 h and 10 h to complete.

### 1.2.8 Participant Performance Analysis

The variance in the participant-reported values for the five fiber metrics (fiber diameter, pore area, porosity, intersection density, length between intersections) were assessed as a measure of precision (the closeness of agreement between test results)[30,31] before and after training. However, only fiber diameter could be assessed for accuracy (the closeness of agreement between a test result and an accepted value)[30,31] because it was the only metric for which acceptable values have been established: fiber diameter of the wires (25.6 μm and 31.1 μm) was verified by light microscopy, scanning electron microscopy, caliper measurements and resistivity measurements [23]. Acceptable values for pore area, porosity, intersection density and length between intersections have not been established and require three-dimensional imaging measurements to ascertain.

### 1.2.9 Statistical Methods

Results of statistical tests are in S1 Table. All data were assessed for normality using skewness, kurtosis and hypothesis tests (Shapiro-Wilk, Shapiro-Francia, Lilliefors, Anderson-Darling, Cramer-von-Mises). For the hypothesis tests P < 0.05 indicates rejection of the null hypothesis that the data is normal. Skewness and kurtosis values that were less than -1 or greater than 1 were considered non-normal. Normal data were plotted as mean with standard deviation and non-normal data were plotted as median with first and third quartiles. Variances were assessed using Levene’s test or Flinger-Killeen’s test as appropriate (based on normality and outliers). For assessing precision, the variance of the Pre-Test was compared to the Post-Test for the five metrics (diameter, pore area, percent porosity, intersection density, and characteristic length). A Fisher’s exact test was used to compare the proportions of participants that identified a bimodal fiber diameter distribution in the Pre-Test vs. the Post-Test. In order to assess fiber diameter accuracy, the errors in the reported fiber diameter values were compared for the Pre-Test vs. the Post-Test (Repeated measures analysis of variance, ANOVA). Finally, for informational purposes only, the means or medians for the Pre-Test vs. the Post-Test were compared using Mann-Whitney or Wilcoxon Signed Rank test as appropriate. No conclusions were drawn from these tests because they were not part of the goal of the current work. For all tests a P < 0.05 was considered significant.

## 1.3 Results

### 1.3.1 Pre- and Post-Test Data

**Fig 2:**
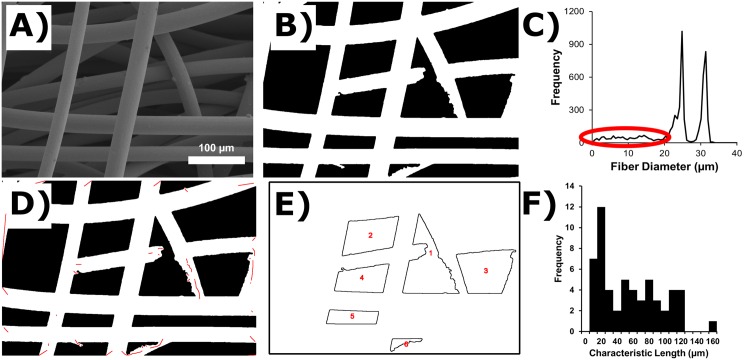
Examples of DiameterJ output. (A) One of the scanning electron micrographs (SEM) that was used in participant performance assessment is shown. Two gauges of steel wire were entangled to make the sample for the image: 48 gauge (31.1 μm dia.) and 50 gauge (25.6 μm dia.). (B) Segmentation of the SEM image in A. (C) Histogram of all diameters that were calculated from the image by DiameterJ. Red oval indicates “noise”. (D) Red lines indicate location of fiber centerlines that had a dia. between 0 and 20 micrometers and that should be considered “noise”. (E) Outlines of pores (pores touching the edges of the image have been removed). (F) Histogram of characteristic lengths between fiber intersections (bin size 10 μm).

Participants were given 6 images of steel wire with two different gauges to analyze in both the Pre-Test and the Post-Test. An example of the images that participants were asked to analyze can be seen in Fig 2A. Participants learned to segment images into a binary image prior to analysis (Fig 2B). DiameterJ can apply 24 different segmentation algorithms to an image so that the user may select the best segmentation. Next, participants used DiameterJ to analyze the segmented images to determine five metrics: fiber diameter, pore area, percent porosity, intersection density and characteristic length between intersections. Fig 2C shows the diameter histogram created by DiameterJ for the image in Fig 2A and 2B. Fig 2C has many small diameters (between 1 μm and 20 μm) recorded by DiameterJ which are circled in red and which appear to be noise. However, if a participant wanted to identify where in the image these diameters were located to ensure that they were not “real” diameters the participants had the option of creating Fig 2D which shows the location of all diameters circled in red in Fig 2C as red centerlines overlaid onto the segmented image. Fig 2E shows an outline of all pores that were analyzed in the image which were used by the participant to calculate the mean pore area and percent porosity. Finally, a histogram of all characteristic lengths of fibers (lengths of wire between wire intersections) can be seen in Fig 2F.

### 1.3.2 Precision and Accuracy of Diameter Measurements

**Fig 3:**
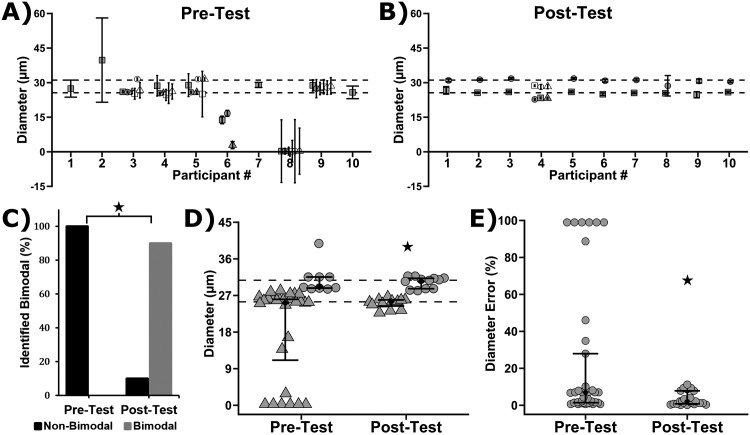
Fiber diameter measurements from 10 participants before and after training. (A,B) Reported fiber diameter and reported standard deviation from each participant in the Pre-Test (A) and Post-Test (B). Note that the test images contained a mix of steel wires with two diameters (48 gauge = 31.1 μm dia.; 50 gauge = 25.6 μm dia.). The dotted lines indicate the known wire diameters. (C) Plot showing prevalence of identifying two diameters of wire (Bimodal) in the steel sample as a function of the Pre-Test and Post-Test. Plot of the number of participants that correctly determined that the test images contained a mix of two wire diameters (bimodal). There was a significant difference between the Pre-Test and Post-Test (Fisher’s Exact Test, P < 0.001). (D) Gray symbols are the individual participant responses and black diamonds are median with first and third quartiles (9 ≤ n ≤ 28). Participant responses that were < 28.35 μm or > 28.35 μm were binned with the 50 gauge (25.6 μm dia., Triangles) or 48 gauge (31.1 μm, Circles) wire, respectively. Note that 28.35 μm is the midpoint between 25.6 μm and 31.1 μm. The star indicates a significant difference between the Pre-Test and Post-Test (variances for 25.6 μm and 3.1. μm were pooled; Levene's Test, 9 ≤ n ≤ 28, P < 0.05). (E) Gray symbols are the errors of the individual participant responses and black diamonds are the error median with first and third quartiles (9 ≤ n ≤ 28). The star indicates a significant difference (2-way analysis of variance, P < 0.05).

Both the Pre-Test and Post-Test asked participants to analyze scanning electron micrographs (SEM) of stainless steel wires with two distinct diameters, 25.6 ± 0.1 μm and 31.1 ± 0.1 μm. Fig 3A shows the Pre-Test and Fig 3B shows the Post-Test results of what each participant reported for fiber diameter and standard deviation. The dotted lines indicate the known diameter of the wires. Visual comparison of the Pre-Test and Post-Test reveals that training results in a general improvement in accuracy (distance between data points and the dotted lines) and precision (spread of the data points). Before training, none of the participants correctly determined that two fiber diameters were present in the sample: participants recorded 1, 3 or 6 diameters (Fig 3C). After training, 9 of 10 participants correctly determined that 2 fiber diameters were present (Fig 3C), which was a significant improvement (S1 Table, Fisher’s Exact Test, P < 0.001).

The range of the participant-reported fibers diameters was 0.25 μm to 39.8 μm for the Pre-Test, while the range was 22.7 μm to 31.8 μm for the Post-Test (Fig 3D), indicating that training served to reduce the range of reported fiber diameters. Training also reduced the variance in the reported diameters in the Post-Test as compared to the Pre-Test (Fig 3D, Levene’s tests, P = 0.03). In addition, the error in fiber diameter measurements as compared to the accepted values (25.6 μm dia. and 31.1 μm) was reduced in the Post-Test (Fig 3D, 2-way analysis of variance, P < 0.05), indicating that the accuracy of participant measurements improved with training. Taken together, these results demonstrate that the precision and accuracy of fiber diameter measurements were improved by completing the DiameterJ training module.

### 1.3.3 Precision of Assessment of Other Scaffold Metrics

**Fig 4:**
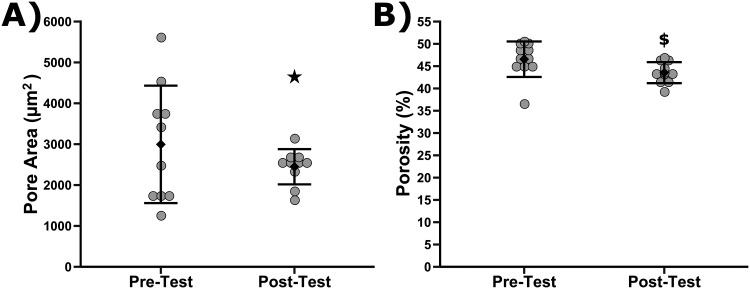
Plot of participant reported pore areas (A) and reported porosity (B) before and after training. Grey symbols are the individual responses while diamonds are mean with standard deviation (10 ≤ n ≤ 11). The star indicates that variance in pore area responses for the Pre-Test vs. Post-Test was statistically different (Levene's Test, P < 0.05). Dollar sign indicates means were statistically different between the Pre-Test and Post-Test (Wilcoxon Signed Rank Test, P<0.05).

After diameter, participants were next asked to analyze pore area and percent porosity. An analysis of the precision of respondents’ answers before and after training was performed in Fig 4. Fig 4A shows the mean pore area reported by participants in the Pre-Test and Post-Test. The Post-Test was statistically smaller than the variance of answers for the Pre-Test, P = 8.83 x 10^−4^. Fig 4B shows the mean percent porosity identified by participants in the Pre- and Post-Test. No differences in the variance of percent porosity values were found between Pre- and Post-Test. Of note is that the average reported percent porosity identified by participants after training did decrease as compared to the Pre-Test to a statistically significant degree (Wilcoxon Signed Rank Test, p = 0.018). The average reported pore area was not different between the Pre- and Post-Test. Accuracy could not be assessed for these metrics because there are no accepted values for these metrics as they are difficult to ascertain from two-dimensional imaging and may require three-dimensional imaging measurements.

**Fig 5:**
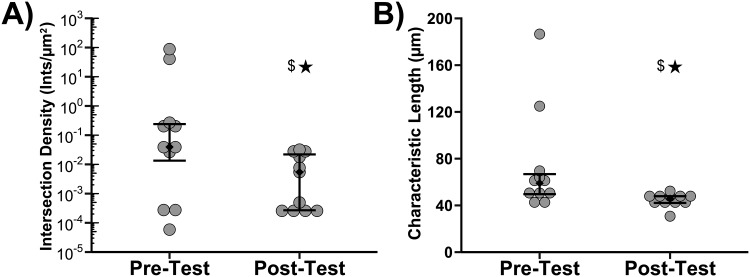
Plot of participant reported intersection density (A) and reported characteristic length between intersections (B) before and after training. Gray symbols are individual responses and diamonds are median with first and third quartiles (n = 11). Stars indicate that variances were statistically different between the Pre-Test and Post-Test (Flinger-Killeen's Test, P < 0.05). Dollar sign indicates means were statistically different between the Pre-Test and Post-Test (Wilcoxon Signed Rank Test, P<0.05).

Finally, participants were asked to report the intersection density and characteristic length of the fibers in both the Pre- and Post-Test. An analysis of the precision of respondents’ answers before and after training was performed in Fig 5. Fig 5A shows the reported mean intersection density of each participant in the Pre- and Post-Test. The variance of answers for the Post-Test was statistically smaller than the variance of answers for the Pre-Test, P = 9.38 x 10^−3^. Note that intersection density was plotted on a log scale and that the Pre-Test answers scaled over 6 orders of magnitude. Fig 5B shows the reported mean characteristic fiber length of each participant in the Pre- and Post-Test. Using Flinger-Killeen's Test it was found that the variance of answers for the Post-Test was statistically smaller than the variance of answers for the Pre-Test, P = 0.013. Of note is that the average reported intersection density and characteristic length identified by participants after training did decrease as compared to the Pre-Test to a statistically significant degree (Wilcoxon Signed Rank Test, p = 0.042 and p = 0.019 respectively).

## 1.4 Discussion

The results demonstrated that participant measurements of fiber morphological properties showed statistically significant improvement in their precision and accuracy after completion of the training. The training module was iteratively improved following test runs by more than 10 participants. The performance results presented herein are from 10 additional students and their feedback has been used to make further improvements that are reflected in the version that is currently available online: https://sites.google.com/site/diameterj/.

Comparability in biomedical research has become a critical issue that has captured the attention of the public [32]. There are many tools to improve confidence in measurement results and improve comparability [33] and user training and standard operating procedures (SOP) are relevant to the current work. User training is an accepted practice for improving reproducibility of measurement processes [34], whereas education and instruction of the operators enables them to improve measurement performance. Training modules frequently require the establishment of an SOP so that trainees get consistent instruction. In one sense, the DiameterJ training module serves as an SOP that the participants have learned to follow. The “inter-participant variability” for the measurement of fiber diameter is represented by the Post-Test data in Fig 3E: the between-participant median error was 2.1%, with first and third quartiles of 1.3% and 5.7%, respectively. In addition, these data indicate that there is a 95% chance that the median error for future participants who complete the training will fall between 1.1% and 2.6% (1-sample Sign test, 95% confidence intervals).

The training above assesses the accuracy of participants’ analysis only for fiber diameter. This is because fiber diameter was able to be independently validated as described by Hotaling et al.[23] However, DiameterJ also produces many other metrics and outputs that are of interest for researchers. These metrics are collectively referred to as “Image Dependent Metrics” because they have been found to change drastically depending on how the image was taken, which segmentation algorithm is used and the focal depth of the image. It has been found that these metrics can vary two to three times their range when analyzing the same image if different contrast settings are used in the SEM when taking the image or in the selection of segmentation algorithm (unpublished data). However, these metrics can serve as relative measures between samples that were imaged under identical microscope settings and analyzed with identical segmentation algorithms. Additionally, for the first time, it has been shown that the consistency of the reported value of these metrics can be increased using the guidelines outlined in the training proposed in this document. The enhanced precision of reported results indicates that if labs use the training proposed here, that the values reported by labs will have more similarity and thus more comparability.

The fibers analyzed in this study were not polymeric nanofibers but instead were stainless steel micro wires and thus concern could be had that the principles outlined here could not apply to other fibrous networks. However, the training was developed independent of scale and material. Segmentation selection criteria were carefully chosen so that they could apply to any image of an interconnected fiber network as was the discussion of the analysis of the various metrics provided by DiameterJ. Additionally, as outlined in the original DiameterJ paper, fiber orientation and size play no role in the accuracy of DiameterJ’s results if images are taken within the range of relative dimensions specified in the training and in the original DiameterJ manuscript. Furthermore, fiber dimensionality is relatively scale invariant and thus, the accuracy of DiameterJ is the same regardless of if an image is taken of small fibers at high magnification or large fibers at low magnification. Therefore, this training prepares participants for analysis of any fibrous network not just those of microfibers or even inorganic nanofibers.

The results above are quantitative in nature. However, qualitative feedback was also received by the authors from many of the study participants. Generally, participants felt that the training was very long but that it did accomplish the goals of the study. When asked how long the total training took participants’ answers varied widely from 3 hours to 10 hours. However, unilaterally all participants indicated that the training would go more quickly if specific examples were given during quizzes that addressed the direct topic being asked in a particular question. Also, participants indicated that a summary sheet showing general analysis methodology would be useful in expediting both processing and analysis of image. This feedback has been incorporated into the version 3.0 of the training that will be released soon and it is hoped that training times will be reduced because of it.

In order to remove bias from the knowledge gained from participants taking the Pre-Test the authors chose to keep the Pre-Test in all future training exercises. The use of this method incorporates the experience of the Pre-Test as part of the training and thus, even if the Pre-Test biases participants’ answers in the Post-Test (either toward getting more accurate/precise answers or less accurate/precise answers) the results presented here reflect the total accuracy/precision gained as a result of the “total” training. The Authors note that one participant did not take the Pre-Test and only went through the training and Post-Test. This participant’s answers were not analyzed in this study. However, the Authors note that their answers are similar (within 0.5 μm) to the means of the diameters given by participants in the Post-Test that took both the Pre- and the Post-Test. While an N of one is not statistically robust the authors believe this result is a good indicator that the Pre-Test does not bias participants’ answers.

Based on feedback received by the initial 10 participants, whose data was not shown here, revisions to the training and the DiameterJ algorithm were made. These updates were extensive and the results from these initial participants were not comparable to the results from those that took the training after the updates. Similarly, feedback from the participants whose data is presented in this study was used to make improvements, such as the addition of better training examples and more targeted coaching during quizzes. Feedback will be collected from participants in the future and may be used to continuously improve the training website. The Authors are continuing to obtain Pre- and Post-Test results from participants who take the training and hope to publish a long-term study in 3–5 years with significantly higher number of participants. The Authors were driven to publish this study now, rather than wait for several more years, for two reasons: 1) to increase the awareness of the training for DiameterJ users and 2) because while the number of participants are relatively small, the results and their statistical significance are very clear. Users of DiameterJ that complete the training improve not only accuracy but also reproducibility of analysis. Even on metrics such as pore area where no ground truth exists, comparability between researchers is significantly enhanced. Thus, after training, morphological properties of scaffolds from SEM images reported by different institutions and labs will be more comparable and will hopefully reduce the amount of irreproducibility found in nanofiber studies.

## 1.5 Conclusion

An open-source training module has been deployed which teaches users how to use the DiameterJ plugin to analyze the morphological properties of fibrous tissue engineering scaffolds that have been imaged by scanning electron microscopy. Testing of user performance before and after training demonstrates that training improved the accuracy and precision of fiber diameter measurements. Training also improved the precision of measurements of pore area, porosity, intersection density and characteristic length between intersections. The enhanced measurement precision among users will help improve research reproducibility by increasing the comparability of results generated by those that complete the training.

## Supporting Information

S1 File File containing all raw data that is present in figures. The sheet is divided into 8 sheets. Each sheet contains data for a different metric that was assessed in the training. Sheets are named based by the metric they have data for.
(XLSX)

S2 File Overview of how to install DiameterJ and basic instructions for how to use DiameterJ. (DOCX)

S3 File Description of the output measures that DiameterJ produces in all files. (DOCX)

S4 File Pre- and Post-Test taken by all participants to show their competency in using DiameterJ. (DOCX)

S5 File First in-depth Training describing how to crop and segment images using ImageJ and the tools provided in DiameterJ. (DOC)

S6 File Second in-depth Training describing how to manually segment images using ImageJ as well as find the pixel to unit conversion factor for images that have scale bars. (DOC)

S7 File Third in-depth training describing how to analyze fibers with DiameterJ. (DOCX)

S8 File Fourth in-depth training describing how to combine the diameter analysis of several images into a single file and analyze the resultant summary statistics. (DOC)

S9 File Fifth in-depth training describing how to interpret, combine and analyze metrics other than the diameter from DiameterJ. (DOCX)

S1 Table Probability values for normality of the data, difference in variance, and differences in means. Probability values below the significance level (0.05) are highlighted in grey. For normality tests, a P value <0.05 indicates non-normal. Also, skewness and kurtosis values not between -1 and 1 were highlighted as these values generally indicated distributions that are non-normal. All other P values less than 0.05 indicate a rejection of the null hypothesis which was that means or variances were equal.
(PPTX)

## References

[1] Wadman M., NIH mulls rules for validating key results, Nature. 500 (2013) 14–16. 10.1038/500014a 23903729

[2] E. Baum, N. Littman, M. Ruffin, S. Ward, K. Aschheim, Key Tools and Technology Hurdles in Advancing Stem-Cell Therapies, California Institute for Regenerative Medicine, 2013. http://alliancerm.org/sites/default/files/Key%20Tools%20and%20Tech%20Hurdles%20in%20Advancing%20Stem%20Cell%20Therapies.pdf (accessed October 7, 2016).

[3] Porat Y., Abraham E., Karnieli O., Nahum S., Woda J., Zylberberg C., Critical elements in the development of cell therapy potency assays for ischemic conditions, Cytotherapy. 0 (n.d.). 10.1016/j.jcyt.2014.08.014 25728414

[4] Pharma and Biotech Survey, Alliance for Regenerative Medicine, 2014. http://alliancerm.org/sites/default/files/ARM_Pharma_SurveyRept_Mar2014_e.pdf (accessed October 7, 2016).

[5] Begley C.G., Ellis L.M., Drug development: Raise standards for preclinical cancer research, Nature. 483 (2012) 531–533. 10.1038/483531a 22460880

[6] Prinz F., Schlange T., Asadullah K., Believe it or not: how much can we rely on published data on potential drug targets?, Nat. Rev. Drug Discov. 10 (2011) 712–712. 10.1038/nrd3439-c1 21892149

[7] J. Lehrer, The Truth Wears Off, New Yorker. (2010). http://www.newyorker.com/magazine/2010/12/13/the-truth-wears-off (accessed October 7, 2016).

[8] D.H. Freedman, Lies, Damned Lies, and Medical Science, The Atlantic. (2010). http://www.theatlantic.com/magazine/archive/2010/11/lies-damned-lies-and-medical-science/308269/.

[9] O.S. Collaboration, Estimating the reproducibility of psychological science, Science. 349 (2015) aac4716 10.1126/science.aac4716 26315443

[10] French A., Bravery C., Smith J., Chandra A., Archibald P., Gold J.D., Artzi N., Kim H.-W., Barker R.W., Meissner A., Wu J.C., Knowles J.C., Williams D., García-Cardeña G., Sipp D., Oh S., Loring J.F., Rao M.S., Reeve B., Wall I., Carr A.J., Bure K., Stacey G., Karp J.M., Snyder E.Y., Brindley D.A., Enabling consistency in pluripotent stem cell-derived products for research and development and clinical applications through material standards, Stem Cells Transl. Med. 4 (2015) 217–223. 10.5966/sctm.2014-0233 25650438 PMC4339854

[11] Hutchinson L., Kirk R., High drug attrition rates|[mdash]|where are we going wrong?, Nat. Rev. Clin. Oncol. 8 (2011) 189–190. 10.1038/nrclinonc.2011.34 21448176

[12] Kaiser J., The cancer test, Science. 348 (2015) 1411–1413. 10.1126/science.348.6242.1411 26113698

[13] Errington T.M., Iorns E., Gunn W., Tan F.E., Lomax J., Nosek B.A., An open investigation of the reproducibility of cancer biology research, eLife. 3 (2014) e04333. 10.7554/eLife.04333 PMC4270077 25490932

[14] Zhu Y., Leong M.F., Ong W.F., Chan-Park M.B., Chian K.S., Esophageal epithelium regeneration on fibronectin grafted poly(l-lactide-co-caprolactone) (PLLC) nanofiber scaffold, Biomaterials. 28 (2007) 861–868. 10.1016/j.biomaterials.2006.09.051 17081604

[15] Lee C.H., Shin H.J., Cho I.H., Kang Y.-M., Kim I.A., Park K.-D., Shin J.-W., Nanofiber alignment and direction of mechanical strain affect the ECM production of human ACL fibroblast, Biomaterials. 26 (2005) 1261–1270. 10.1016/j.biomaterials.2004.04.037 15475056

[16] Ku S.H., Park C.B., Human endothelial cell growth on mussel-inspired nanofiber scaffold for vascular tissue engineering, Biomaterials. 31 (2010) 9431–9437. 10.1016/j.biomaterials.2010.08.071 20880578

[17] Bartneck M., Heffels K.-H., Bovi M., Groll J., Zwadlo-Klarwasser G., The role of substrate morphology for the cytokine release profile of immature human primary macrophages, Mater. Sci. Eng. C. 33 (2013) 5109–5114. 10.1016/j.msec.2013.08.028 24094233

[18] Li Y., Zhang C., Wu Y., Han Y., Cui W., Jia L., Cai L., Cheng J., Li H., Du J., Interleukin-12p35 Deletion Promotes CD4 T-Cell–Dependent Macrophage Differentiation and Enhances Angiotensin II–Induced Cardiac Fibrosis, Arterioscler. Thromb. Vasc. Biol. 32 (2012) 1662–1674. 10.1161/ATVBAHA.112.249706 22556333

[19] Xin X., Hussain M., Mao J.J., Continuing differentiation of human mesenchymal stem cells and induced chondrogenic and osteogenic lineages in electrospun PLGA nanofiber scaffold, Biomaterials. 28 (2007) 316–325. 10.1016/j.biomaterials.2006.08.042 17010425 PMC4035020

[20] Silva G.A., Czeisler C., Niece K.L., Beniash E., Harrington D.A., Kessler J.A., Stupp S.I., Selective Differentiation of Neural Progenitor Cells by High-Epitope Density Nanofibers, Science. 303 (2004) 1352–1355. 10.1126/science.1093783 14739465

[21] Pilehvar-Soltanahmadi Y., Akbarzadeh A., Moazzez-Lalaklo N., Zarghami N., An update on clinical applications of electrospun nanofibers for skin bioengineering, Artif. Cells Nanomedicine Biotechnol. (2015) 1–15. 10.3109/21691401.2015.1036999 25939744

[22] Stanger J.J., Tucker N., Buunk N., Truong Y.B., A comparison of automated and manual techniques for measurement of electrospun fibre diameter, Polym. Test. 40 (2014) 4–12.

[23] Hotaling N.A., Bharti K., Kriel H., Simon C.G. Jr., DiameterJ: A validated open source nanofiber diameter measurement tool, Biomaterials. 61 (2015) 327–338. 10.1016/j.biomaterials.2015.05.015 26043061 PMC4492344

[24] Schneider C.A., Rasband W.S., Eliceiri K.W., NIH Image to ImageJ: 25 years of image analysis, Nat. Methods. 9 (2012) 671–675. 22930834 10.1038/nmeth.2089 PMC5554542

[25] Schindelin J., Arganda-Carreras I., Frise E., Kaynig V., Longair M., Pietzsch T., Preibisch S., Rueden C., Saalfeld S., Schmid B., Tinevez J.-Y., White D.J., Hartenstein V., Eliceiri K., Tomancak P., Cardona A., Fiji: an open-source platform for biological-image analysis, Nat. Methods. 9 (2012) 676–682. 10.1038/nmeth.2019 22743772 PMC3855844

[26] Hotaling N.A., Bharti K., Kriel H., Simon C.G. Jr., Dataset for the validation and use of DiameterJ an open source nanofiber diameter measurement tool, Data Brief. 5 (2015) 13–22. 10.1016/j.dib.2015.07.012 26380840 PMC4556745

[27] Buck S., Solving reproducibility, Science. 348 (2015) 1403–1403. 10.1126/science.aac8041 26113692

[28] Goldstein J., Practical Scanning Electron Microscopy: Electron and Ion Microprobe Analysis, Springer Science & Business Media, 2012.

[29] Watt I.M., The Principles and Practice of Electron Microscopy, Cambridge University Press, 1997.

[30] ISO 5725–1:1994—Accuracy (trueness and precision) of measurement methods and results, ISO, 2016.

[31] Joint Committee for Guides in Metrology, International Vocabulary of Metrology—Basic and General Concepts and Associated Terms 200:2012, 3rd ed., JCGM, 2015.

[32] Rigor and Reproducibility, Natl. Inst. Health NIH. (n.d.). https://www.nih.gov/research-training/rigor-reproducibility (accessed October 14, 2016).

[33] Simon C.G., Lin-Gibson S., Elliott J.T., Sarkar S., Plant A.L., Strategies for Achieving Measurement Assurance for Cell Therapy Products, Stem Cells Transl. Med. 5 (2016) 705–708. 10.5966/sctm.2015-0269 27386605 PMC4878336

[34] Collins F.S., Tabak L.A., Policy: NIH plans to enhance reproducibility, Nat. News. 505 (2014) 612. 10.1038/505612a PMC4058759 24482835

