# Supplementary material for: Training to Improve Precision and Accuracy in the Measurement of Fiber Morphology
Source: PLoS One. 2016 Dec 1;11(12):e0167664. doi: 10.1371/journal.pone.0167664 (PMC5132175; doi:10.1371/journal.pone.0167664)
Supplement: S2 File — (DOCX) [file pone.0167664.s002.docx]

A more up-to-date version of this document can be found [here](https://docs.google.com/document/d/1lODEj02e2eFtQ-v0B3TJd53Q2XFZZW2i0szrfyX0kRY/edit?usp=sharing).

Starting out:

Download and install [ImageJ 1.48](http://rsbweb.nih.gov/ij/download.html) or newer or [Fiji](http://fiji.sc/Downloads) (any version). If you installed imageJ before the end of 2013 you should uninstall your current version of ImageJ (DO NOT UPDATE) and reinstall ImageJ 1.48 or newer. Before uninstall be sure to copy all of your old plugins into a separate folder as these will be removed when you uninstall your old version of ImageJ.

Download and unzip the files and move or copy the three folders (or one folder in the FIJI version) into the plugins folder of ImageJ/FIJI.

If you do not know where this is generally, for any system, navigate to the directory where ImageJ is installed. A folder in the ImageJ folder will be called “Plugins.” Simply move the file(s) into this folder and restart ImageJ/FIJI.

For more questions please see <http://imagej.net/Plugins> .

For ImageJ in windows that should be in directory:

"C:\Program Files\ImageJ\plugins"

Or

"C:\Program Files (x86)\ImageJ\plugins"

Image Segmentation:

***Do this first if your image is not a binary image (only black and white pixels):***

1. Go to: “Plugins-->DiameterJ-->Segment Mixed” or “Plugins-->DiameterJ-->Segment SRM” to segment images.
   1. Each uses a different set of 8 algorithms to analyze and segment your images.
2. Choose the directory of the file you want to segment
3. Segment XXX will then ask if you want to crop your image. If you do then it will ask what the height and width in pixels you want to crop your images to. The crop box begins at the top left of your image. If your image is 1280x960 and you want to crop out the scale bar at the bottom which takes up 100 of the bottom pixels of the image then you would say the width is 1280 while the height is 860.
   1. This is a crude crop in that it only crops rectangles starting at the top left of your image. For more complex cropping please see online tutorials on the crop tool in ImageJ.
4. The code will then produce three folders in the folder where the image that you selected for analysis is located. One folder called “Best Segmentation” another called “Montage Images” and the third called “Segmented Images".
   1. The “Best Segmentation” folder contains no images.
   2. The “Montage Images” have a comparison of the original image to the 8 different segmentation methodologies. Look at the montage images and find the image with the most accurate representation of your fibers. (Names are on the bottom of the images in Red.)
      1. Remember, not all fibers need to be included in the segmentation for it to be a “good” segmentation. The fibers just need to be without significant holes, edge defects, or too densely packed for the program to analyze them. (the program does not analyze intersections of fibers)
   3. The “Segmented Images_XXX" folder has all of the segmented images from the montage images. Copy and paste the best segmentation into the “Best Segmentation” folder.
5. Visually inspect these images to make sure that the segmentation worked well and that the segmentation represents the image you want to analyze. If it does not try the other segmentation algorithm.
   1. If none of the 16 algorithms segment your image well you can take the best segmentation and manually fix the errors to accurately reproduce the fibers.
6. After copying the best image into the “Best Segmentation” folder delete all unused images/montage images.

Pixel to Unit Distance Transformation

***All measures given by DiameterJ are in pixels by default***

1. To determine the pixel to unit distance (nanometer/micrometer/millimeter) conversion there are two options depending on how much metadata your SEM stores with your image. It is possible that it stores the pixel to distance conversions in the metadata and thus getting that number is easy. Simply go to:
   1. Analyze-->Set Scale
2. Look at what the unit of length is… if its inches then your SEM doesn’t store that information with the micrograph and you’ll have to do the conversion by hand.
3. In that case open any micrograph at the magnification that you have taken your SEM images in ImageJ.
4. In ImageJ/FIJI go to menu:
   1. Analyze-->Set Scale
5. Click “Click to Remove Scale”
6. Close the scale window
7. Place your mouse over the scale bar and hit the “+” key to zoom-in.
8. Continue to zoom-in on the scale bar until it fills the screen
   1. After zooming in you can widen the default width of the window that ImageJ chooses just like any normal window).
9. Next, select the “Rectangular” tool from the menu bar.
   1. If the rectanglular tool isn't available go to the tile that has “>>” and click “Restore startup tools” from the drop down menu.
10. Next, draw a square or a line that spans the scale bar.
11. To get the length or width of this rectangle simply look under the tool bar of ImageJ as you are drawing the rectangle
    1. As soon as you stop drawing the measurements disappear so don’t let go of the cursor until you’ve seen the rectangle width.
12. Record the number that 'w" equals.
13. Then it’s simply a matter of dividing the length represented by the scale bar by the number of pixels you just recorded.
14. Take that conversion factor and multiply all pixel measurements output by DiameterJ and you’ll have the distance in your desired units. (Percent measures do not need to be multiplied)
15. For the DiameterJ outputs of pore area, percent porosity, and intersection density you will do a different conversion then simply multiplying the length conversion.
    1. The pore area is an area and thus you should square the conversion factor calculated above and multiply by the pixel value given in the pore area.
    2. The percent porosity is already a percent and thus does not need to be multiplied by anything.
    3. The intersection density is reported as number of intersections per 100px x 100px area. This is an arbitrary unit and thus to convert it to intersections per unit area divide the "Intersection Density" by 10,000. Next, multiply it by 1 over the square of the conversion factor above. That will give you intersections per unit squared.

DiameterJ Analysis

***Analyzed images MUST be white fibers on a black background. If you have black fibers on a white background invert the image to analyze it with DiameterJ.***

In ImageJ/FIJI go to:

“Plugins-->DiameterJ-->DiameterJ 0.XXX” to analyze the segmented image.

Choose the directory of the file you want to analyze (right now it’s in batch form so if you only want to analyze one file move it into a folder by itself and select that folder. I intend to post both a batch and non-batch form of the segmentation and DiameterJ code but haven’t made the non-batch form yet.)

DiameterJ will then analyze all images in the directory and will separate its analysis into three folders “Diameter Analysis Images”, “Histograms”, and “Summaries.” The file labeled “DiameterJ Output Descriptions.docx” describes what the outputs are in each of these folders.

DiameterJ Location

***Analyzed images MUST be white fibers on a black background. If you have black fibers on a white background invert the image to analyze it with DiameterJ.***

If you are curious about where particular radii occur in your image a tool called “DiameterJ Loc” can be used.

In ImageJ/FIJI go to:

“Plugins-->DiameterJ-->DiameterJ Loc” to analyze the segmented image.

Select the lower and upper pixel diameter you wish to visualize in your image. A folder will be created called “Diameter Location” with an image of red lines over all diameters where DiameterJ measured a particular diameter or range of diameters that you specified.
