# Supplementary material for: Training to Improve Precision and Accuracy in the Measurement of Fiber Morphology
Source: PLoS One. 2016 Dec 1;11(12):e0167664. doi: 10.1371/journal.pone.0167664 (PMC5132175; doi:10.1371/journal.pone.0167664)
Supplement: S3 File — (DOCX) [file pone.0167664.s003.docx]

DiameterJ Output:

A more up-to-date version of this document can be found [here](https://docs.google.com/document/d/1n6a8VPy4tugZTQO7BYD-JU4JYvrmbuSib2AcXPXys9w/edit?usp=sharing).

For the below text “XXXX” represents whatever the image name was that was analyzed.

# Summaries Folder:

## XXXX_Total Summary.csv

1. Super Pixel: The mean fiber diameter as calculated using a super pixel determination
2. More accurate for samples with extremely diverse fiber diameters
3. Hitogram_Mean: The mean fiber diameter as calculated using the average of all diameters measured in the histogram.
4. Histogram_SD: The standard dev. Of the fiber diameter as calculated using all diameters measured in the histogram.
5. Histogram_Mode: Most occurring fiber diameter in the histogram
6. Histogram Median: Middle fiber diameter in the histogram
7. Mean Pore Area: (Total number of black pixels counted in pores) / (Total number of pores in image)
8. Pore Area SD: The standard deviation of all pore areas measured
9. Min. Pore Area: The minimum pore area measured
10. Max. Pore Area: The maximum pore area measured
11. Percent Porosity: (Total number of black pixels) / (Total pixels in an image)
12. Intersection Density (100x100px): (Number of fiber overlaps)*10000 / (Total pixels in image)
13. Characteristic Length: (Total length of all fiber centerlines) / (# of fiber overlaps)

# Histograms Folder:

## XXXX_Histogram.csv

1. Mean: Average Diameter from all measures found in the histogram
2. StdDev: Standard deviation from all measures found in the histogram
3. Mode: Most occurring diameter found in the histogram
4. IntDen: The product of Area and average radius
5. Median: Middle value in the histogram
6. RawIntDen: The sum of the radii at all pixels in the image or selection
7. Radius Value: Radius length (in pixels)
8. Radius Count: Number of times the radius value occurred in the image. Also known as the frequency of occurrence and can also be interpreted as the length of fiber in an image that has a given radius.
   1. The radius or diameter histogram is constructed from the radius value (x-axis) and radius count (frequency of occurrence on y-axis)

## XXXX_Intersection Coordinates.txt

1. Column 1 – Grey scale value at intersection
2. Column 2 – x coordinate of intersection (upper left hand corner is 0,0)
3. Column 3 – y coordinate of intersection (upper left hand corner is 0,0)

## XXXX_Pore Data.csv

1. Column 1: A unique number identifier for each pore counted in the image
2. Label: Contains the Mean, SD, Min, and Max labels at the bottom of every list. These rows represent the summary metrics (mean, standard deviation, minimum value, and maximum value) for the entire column above them.
3. Area: Total number of black pixels in an image not in groups touching the sides.
4. Perim.: The number of pixels in the perimeter of each pore.
5. Major: The length of the primary axis of the best fitting ellipse for each pore.
6. Minor: The length of the secondary axis of the best fitting ellipse for each pore.
7. Angle: The angle between the major axis of the pore and a line parallel to the X-axis of the image.
8. Circ.: 4π × [Area] / [Perimeter^2] with a value of 1.0 indicating a perfect circle. As the value approaches 0.0, it indicates an increasingly elongated shape. Values may not be valid for very small particles. Uses the heading Circ.
9. AR: The aspect ratio of the particle’s fitted ellipse, i.e., [Major Axis] / [Minor Axis].
10. Round: 4 × [*Area*] / (*π* × [*Major* *axis*]^2^) or the inverse of *Aspect Ratio*.
11. Solidity: [*Area*] / [*Convex* *area*]

## XXXX_Radius Histogram.tif

1. Image of the histogram of all fiber radii in the image.

# Diameter Analysis Images Folder

## XXXX_Axial Thinning.tif

1. Image of the centerline as determined by the axial thinning algorithm

## XXXX_Dilate.tif

1. Image of all centerlines counted in the histogram overlaid on the Euclidean distance transformed of the fibers fibers.
   1. Yellow lines are the locations where radii were counted
   2. Fibers are in greyscale as transformed by the Euclidean distance transform

## XXXX_Orientation.tif

1. An image with the frequency of orientation of the centerline of all fibers. This is an output of OrientationJ and was not coded by me.

## XXXX_Pores.tif

1. Image of all pores measured by the algorithm, along with their unique number identifier.

## XXXX_Voronoi.tif

1. Image of all centerlines as determined by the Voronoi tessellation algorithm.
