# Supplementary material for: Training to Improve Precision and Accuracy in the Measurement of Fiber Morphology
Source: PLoS One. 2016 Dec 1;11(12):e0167664. doi: 10.1371/journal.pone.0167664 (PMC5132175; doi:10.1371/journal.pone.0167664)
Supplement: S4 File — (DOCX) [file pone.0167664.s004.docx]

## Nanofiber Diameter Metric Assessment

### ****Overview of Study****

This form is designed for you to input the results of your fiber diameter analysis. Please **do not** discuss how you did your analysis, what you did for your analysis, or the results of your analysis with anyone until the study has been completed.

 We are trying to show how a protocol improves comparability between results and thus telling others how you did your analysis can, and probably will, greatly change the way that they do it. If you are struggling to complete the analysis with the given directions please just make your best guess as to what we are asking for and answer the questions accordingly.

This zip file contains six images of a single sample. The samples are called "Steel_07 - Steel_12".  Your job will be to analyze the images and enter your answers in the Pre-Test form.  The answers you place in the form should reflect the combined analysis of all six replicate images of the steel sample.

There are 11 questions found below, please have the answers to these prior to beginning the form submission. You will need the 6 digit unique personal identifier you typed in from the quiz. If you do not have this number, please contact Nathan.Hotaling@gmail.com to obtain it.

1. What is your ID number? - Fill in the blank
2. How many fiber diameters did you calculate for the Steel samples? - Toggle
3. What is the Mean and standard deviation of this(these) diameters? - Fill in the blank
4. What are the units you used for each of these measures? - Toggle
5. How did you calculate this mean and standard deviation? - Multiple choice
6. What did you find the following values to be for the Steel samples? - Fill in the blank
7. Mean pore area
8. Standard deviation of pore areas
9. Percent porosity
10. Std. Dev. Percent Porosity
11. What units did you use for each of these measures?
12. How did you calculate the pore metrics in 6? - Paragraph
13. What did you find the following values to be for the Steel samples? - fill in the blank
14. Mean intersection density for a 10 μm x 10 μm area
15. Mean characteristic length
16. What units did you use for each of these measures?
17. How did you calculate the intersection density and characteristic length measurements?

If you did not download the images on from previous training you can download them here: https://goo.gl/A7Up7w

***At no point during this assignment is any personal information obtained or recorded. All data will be analyzed using the 6 digit number provided by the user and no link between that reference number and a personal identity is stored or obtained.***

## Participant ID

### 1) ****What is your ID number from the Quiz?*****

_________________________________________________

## Fiber Diameter Metrics

### *****Remember the images are from replicates of the same sample. Thus, here you should indicate how many values you calculated based on a combination of all images from a sample type, not metrics for each image.*****

#### 2) ****What is the global average fiber diameter(s) and standard deviations(s) of the steel sample? If more than one enter each separately below.****      Remember to report values in real units, not in pixel units. (If more than 5 diameter values have been found please separate their values by commas in the last box.)*

|  | **Average** | **Standard Deviation** |
| --- | --- | --- |
| Diameter 1 | ___________________ | ___________________ |
| Diameter 2 | ___________________ | ___________________ |
| Diameter 3 | ___________________ | ___________________ |
| Diameter 4 | ___________________ | ___________________ |
| Diameter 5 | ___________________ | ___________________ |
| Diameters > 5 | ___________________ | ___________________ |

#### 3) What are the units you used for each of these measures?*

|  | **Pixels** | **Nanometers** | **Micrometers** | **Millimeters** | **Unitless** |
| --- | --- | --- | --- | --- | --- |
| Steel | ( ) | ( ) | ( ) | ( ) | ( ) |

## How and when you obtained mean and SD values

#### 4) ****How did you pre-process the data before calculating the mean and standard deviation?**** Choose the answer that best fits what you did for each step.*

( ) Obtained the Super Pixel diameter for each of the 6 images from the XX_Total Summary.csv file for each sample.

( ) Obtained the Histogram_Mean diameter for each of the 6 images from the XX_Total Summary.csv file for each sample.

( ) Obtained the Histogram_Mode diameter for each of the 6 images from the XX_Total Summary.csv file for each sample.

( ) Obtained the Histogram_Median diameter for each of the 6 images from the XX_Total Summary.csv file for each sample.

( ) Gaussian peak fit the main peaks from the histogram for each image, recorded the peak center and FWHM for each image and each peak for each sample.

( ) Summed frequencies of identical radius from all 6 image histograms and Gaussian peak fit the main peaks for each sample

( ) Other: _________________________________________________

#### 5) ****How did you calculate the means?**** Choose the answer that best fits what you did for each step.*

( ) Mean of the 6 Super Pixel diameter measurements

( ) Mean of the 6 Histogram_Mean diameter measurements

( ) Mean of the 6 Histogram_Mode diameter measurements

( ) Mean of the 6 Histogram_Median diameter measurements

( ) Mean of the centers of the Gaussian Peak fits from each image for each set of peaks

( ) The center of each Gaussian peak fit in the cumulative histogram.

( ) Other: _________________________________________________

#### 6) ****How did you calculate these standard deviations?**** Choose the answer that best fits what you did for each step.*

( ) Std. dev. of the 6 Super Pixel diameter measurements

( ) Std. dev. of the 6 Histogram_Mean diameter measurements

( ) Std. dev. of the 6 Histogram_Mode diameter measurements

( ) Std. dev. of the 6 Histogram_Median diameter measurements

( ) Average of the 6 Histogram_SD values from the XX_Total Summary.csv file.

( ) Calculated grand variance/composite variance using Histogram_SD values and the total frequency of the counts from each image.

( ) For each image find the FWHM of each of the Gaussian Peak fits, convert FWHM to SD, average SD values across images to get a total SD for each set of peaks.

( ) For each image find the FWHM of each of the Gaussian Peak fits, convert FWHM to SD, calculated grand variance/composite variance from SD and frequency values.

( ) For the cumulative histogram of all 6 images found the FWHM of each of the Gaussian Peak fits and converted them to SD.

( ) Other: _________________________________________________

#### 7) ****When did you convert the units?**** Choose the answer that best fits what you did for each step.*

( ) Did not convert units (couldn't figure out how)

( ) Converted units before getting the mean and SD

( ) Converted units before Gaussian peak fitting

( ) Converted units after getting mean and SD

( ) Converted units after Gaussian peak fitting

( ) Other: _________________________________________________

## Pore Metrics

#### 8) ****What are the values of the metrics listed below?****      Remember to report values in real units, not in pixel units.*

|  | **Average** | **Standard Deviation** |
| --- | --- | --- |
| Pore Area | ___________________ | ___________________ |
| Percent Porosity | ___________________ | ___________________ |

#### 9) ****What are the units you used for each of these measures?****

|  | **Pore Area** | **Percent Porosity** |
| --- | --- | --- |
| **Pixels** | ( ) | ( ) |
| **Nanometers** | ( ) | ( ) |
| **Micrometers** | ( ) | ( ) |
| **Nanometers Squared** | ( ) | ( ) |
| **Micrometers Squared** | ( ) | ( ) |
| **Percent** | ( ) | ( ) |
| **Unitless** | ( ) | ( ) |

### 10) ****How did you calculate each of the pore metrics from above?*****

____________________________________________

____________________________________________

____________________________________________

____________________________________________

## Nanofiber Intersection Density and Characteristic Length

#### 11) ****What are the values of the metrics listed below?****      Remember to report values in real units, not in pixel units*

|  | **Average** | **Standard Deviation** |
| --- | --- | --- |
| Intersection Density for a 10 μm x 10 μm area | ___________________ | ___________________ |
| Characteristic Length | ___________________ | ___________________ |

#### 12) ****What are the units you used for the characteristic length?****

|  | **Pixels** | **Nanometers** | **Micrometers** | **Millimeters** | **Unitless** |
| --- | --- | --- | --- | --- | --- |
| Characteristic Length | ( ) | ( ) | ( ) | ( ) | ( ) |

### 13) ****How did you calculate the density and length values from above?*****

____________________________________________

____________________________________________

____________________________________________

____________________________________________

## Thank You!

### ****Congratulations!  You have completed the preliminary training analysis of the SEM images.  You are half-way to completing this analysis!**** ****Please go to the link below to continue your training.  Read the protocol and answer all questions carefully and follow the analysis steps to the best of your ability.  Once you have completed the protocol and training, analyze the new set of images using your newly found analysis skills.  Submit your answers by returning to this survey, and filling it out again. Note:  Do not edit your answers to this survey, begin a new survey with your new answers.**** [Advanced Training](http://goo.gl/forms/6d2xZL5AgR)
