# Supplementary material for: Training to Improve Precision and Accuracy in the Measurement of Fiber Morphology
Source: PLoS One. 2016 Dec 1;11(12):e0167664. doi: 10.1371/journal.pone.0167664 (PMC5132175; doi:10.1371/journal.pone.0167664)
Supplement: S5 File — (DOC) [file pone.0167664.s005.doc]

# Image Cropping and Segmentation

## Introduction:

The first step to analyzing any image is to crop out features of the image that you would not like to measure. Typically, this occurs in images with scale bars or information panels in them. These areas are not representative of your sample and thus must be removed. Once cropped the image should then be divided or “segmented” into features that you will measure and those that you do not.

## Cropping images

1. DiameterJ’s segmentation algorithms include a very basic cropping function that allows the user to crop out any information panels or scale bars that are in the right or bottom sides of the image. Below is a step by step explanation of what the cropping feature is, what the feature does, and how to use this feature.
2. Open ImageJ

1. Go to:  “Plugins-->DiameterJ-->Segment Mixed” or “Plugins-->DiameterJ-->Segment SRM” to segment images.  (Either option is generically referred to as Segment XX below). Figure 1 below shows the menu.


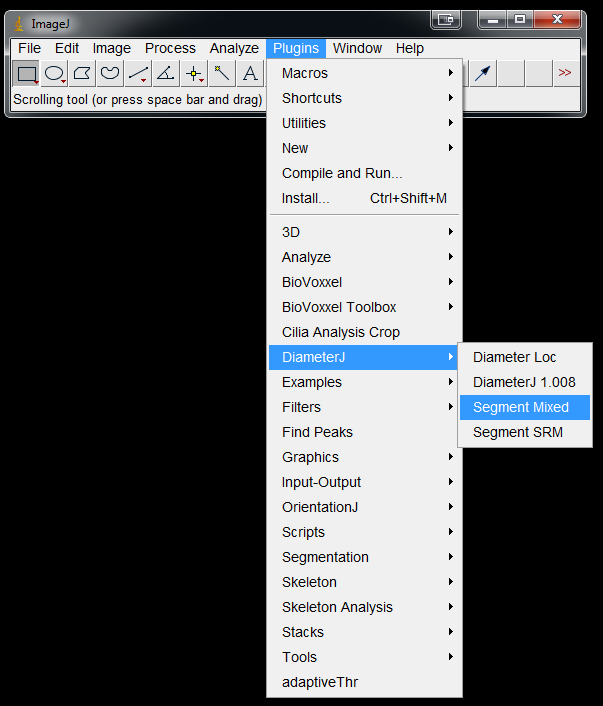


**Figure 1: DiameterJ plugin location in ImageJ.**

1. Choose the directory of the file(s) you want to segment. Figure 2 shows an example directory window.


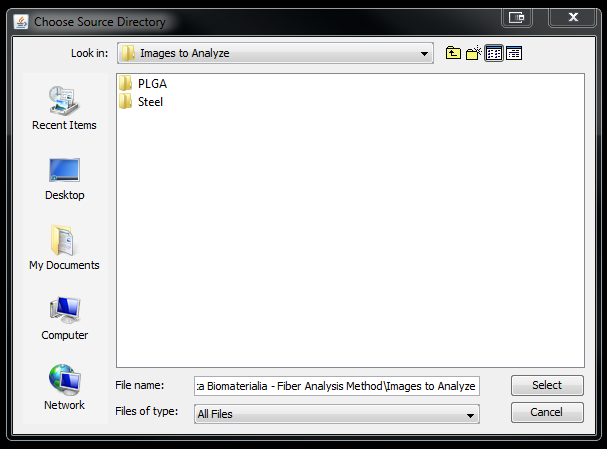


**Figure 2: Directory location example.**

1. Segment XX will then ask if you want to crop your image, by entering 1 (i.e., yes, I would like to crop my image) or 2 (i.e., do not crop my image). If you enter 1 (i.e., yes):
2. DiameterJ opens the image and removes all scaling on the image so that all units are pixels. If you were to look at the image it would appear like the image as shown in Figure 3. A) Shows the pixel location of the cursor (in this case in the top left hand corner of the image) and its grey value. B) Shows the dimensions of the image (in pixels expressed in image width x height), the type of image (in this case 8-bit) and the image size. A diagram of this process can be seen in Figure 3 below.


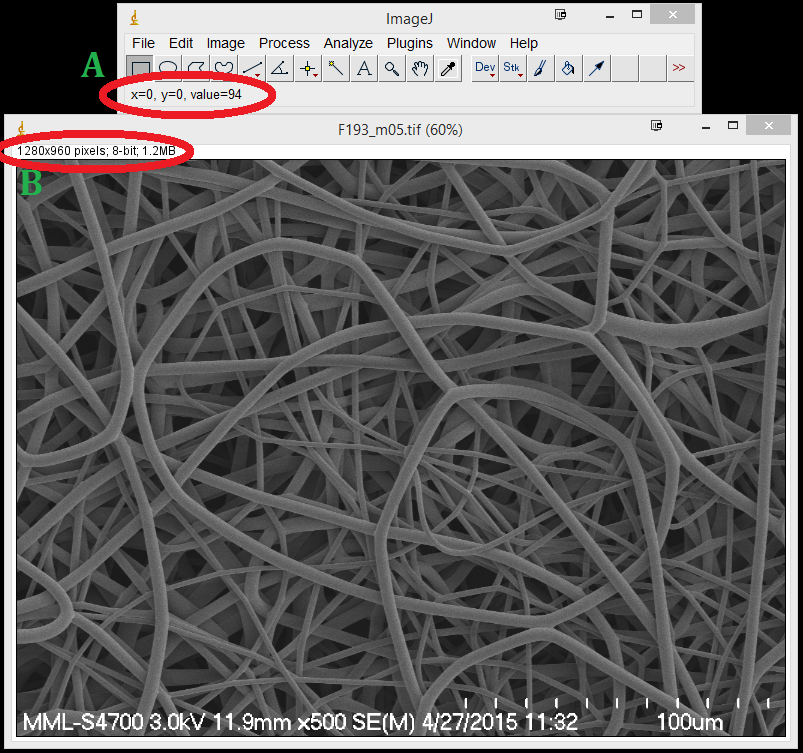


**Figure 3: Cursor and image information.**

1. DiameterJ then asks the user the width and the height that they would like to crop the image to. These values define the bottom right hand corner of a cropping box with the top left corner being located at the top left hand corner of the image (i.e., at image coordinates 0,0).

1. Below in Figure 4 is an example image with a cropping box that the user defined, in DiameterJ, as 1250 pixels wide by 890 pixels in height.
   1. **Note** that when using the standard box drawing tool in ImageJ or FIJI, the dimensions of the box that you are drawing (w = width, h = height) is dynamically displayed in the bottom information menu bar of the ImageJ GUI, shown circled in red in Figure 4.
2. Based on the user specified width and height, the image is then cropped to the exact dimensions of the box, and this cropped image is passed on for further segmentation in DiameterJ.
   1. **Note** in the above case, the width could have been specified as 1280 pixels (the total width of the image) because there are no artifacts (such as scale bars) on the right side of the image
3. In the below example, shown in Figure 5, both a scale bar and a menu on the right hand side of the image must be cropped out of the image. In this case, the image is 1280 x 891 pixels, the menu is 144 pixels wide and the scale bar is located 103 pixels above the bottom of the image. Thus, for this case, when the prompts are shown in DiameterJ the crop for these dimensions is defined as:
   1. Image width = 1280-144 = 1136 and the user would enter 1136 as the image width.
   2. Image height = 891-103 = 788 and the user would enter 788 as the image height.


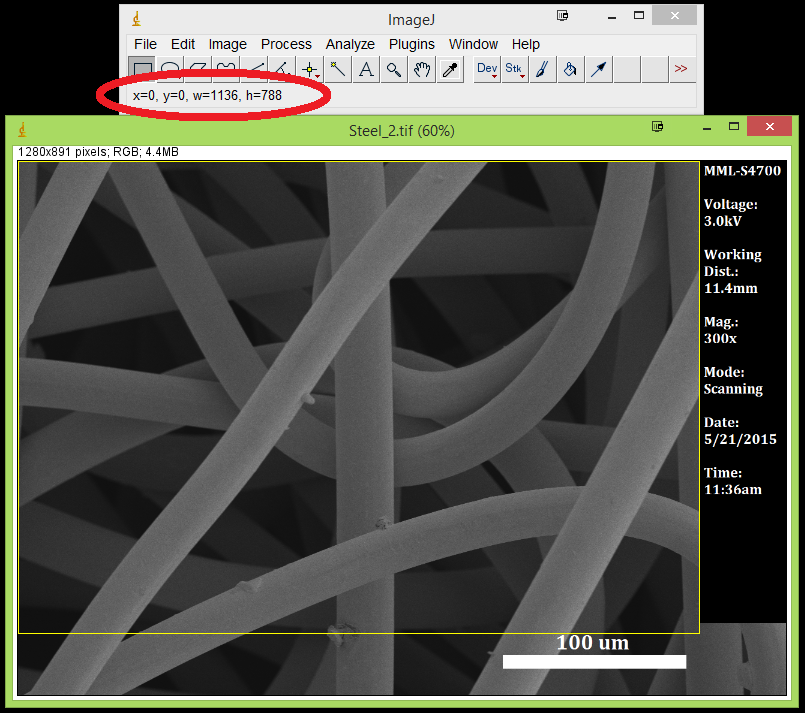


**Figure 5: Second cropping example: side and bottom cropping**

## Image Segmentation

As a refresher from the “[Installation and User Instructions](https://docs.google.com/document/d/1lODEj02e2eFtQ-v0B3TJd53Q2XFZZW2i0szrfyX0kRY/edit?usp=sharing)” document, image segmentation is the process of converting an image into a black and white (i.e. binary) representation of your image where the white pixels represent the portions of the original image that you would like to measure and the black pixels represent the background that are not measured.

Once you have entered the dimensions you want to crop all of your images to, Segment XX in DiameterJ then crops the images and runs through 8 different algorithms.

1. The DiameterJ code then produces three new folders that are located within the folder where the image that you selected for analysis is located.  One folder is called “Best Segmentation” another is called “Montage Images” and the third is called “Segmented Images_mix" or “Segmented Images_SRM".
   1. The “Best Segmentation” folder contains no images.
   2. The “Montage Images” folder contains a comparison of the original image to the 8 different segmentation methodologies.  In this folder, you will find the montage images and find the segmented image with the most accurate representation of your fibers.
      1. Names are displayed on the bottom of the images in red, as shown in Figure 6.
   3. The “Segmented Images_XXX" folder contains all of the segmented images that were shown in the montage images. From this folder, you will copy the image file that represents the best segmentation and paste this image file into the “Best Segmentation” folder.
2. Figure 6 shows an example of a sample montage image.


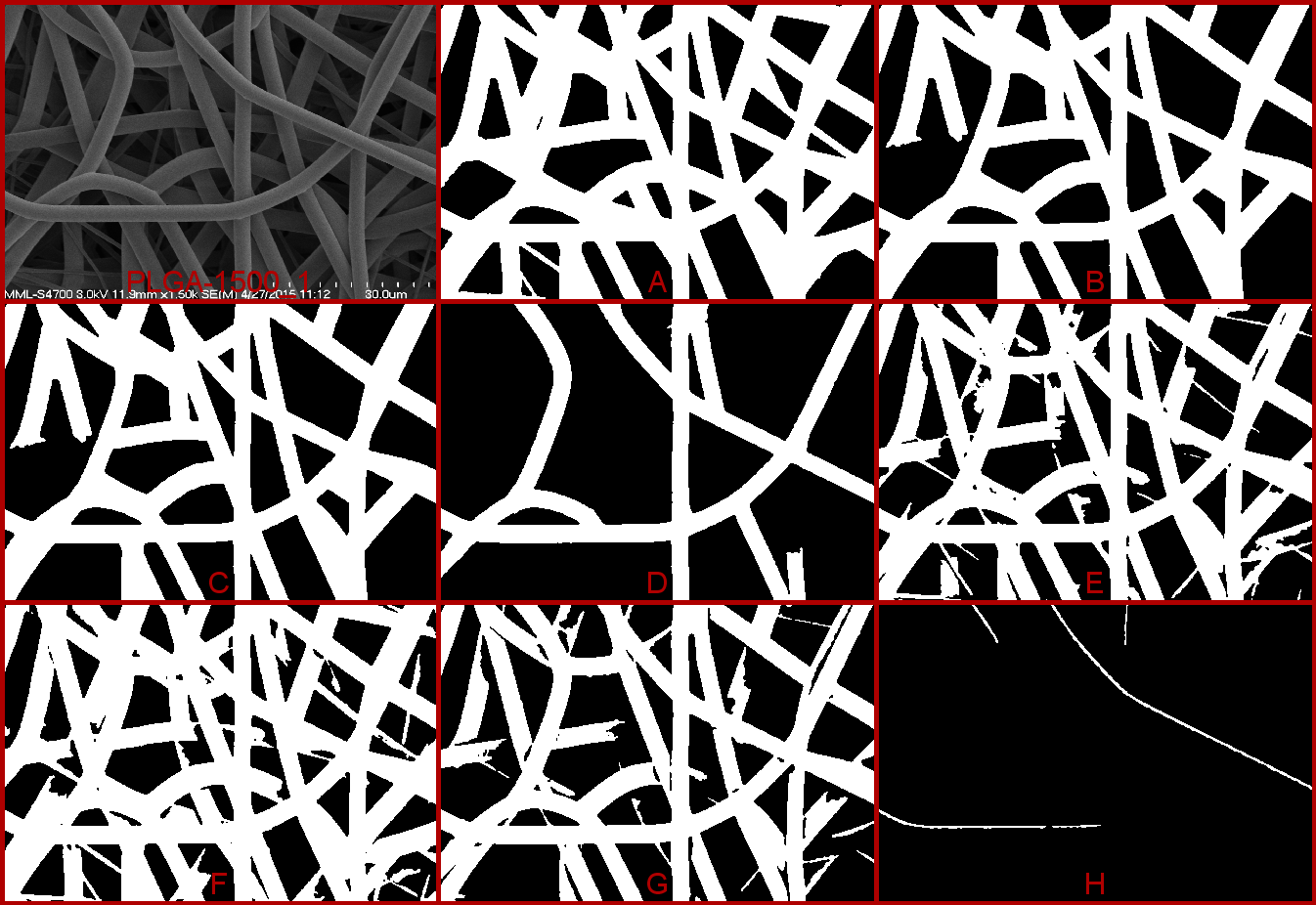


**Figure 6: Montage image example.**

1. In Figure 6, the user would compare each of the segmentations (e.g., A, B, C, etc.) with the original image (i.e., PLGA-1500_1) and find which image best represents the fibers in the original image.
2. To select the image that “best” represents the original, users should look for 5 key characteristics of each segmented image which will be further explained below:
   1. No partial fiber segmentations are present.
   2. Intersections of fibers do not contain black spots (i.e. holes).
   3. Segmented fibers are representative of actual fibers in the image and are not background/imaging artifacts
   4. Segmentations accurately represent fibers’ actual diameter
   5. In the case that all of the above are equally represented in the segmented images of the montage image, choose the image with more fibers in it. Thus, always choose the image with the most representative fibers that are whole (i.e. not partial).
3. Partial segmentations are defined as fibers that do not segment completely in the radial direction. For example in Figure 6, G and E have many partial fibers while A, B, C, and D do not. Figure 7 highlights partial fiber segmentations by circling several of them in red, and comparing them to A and B which have little to no partial fibers. Segmented images with many partial fibers should not be used for analysis or should manually corrected by the user before they are analyzed in DiameterJ.


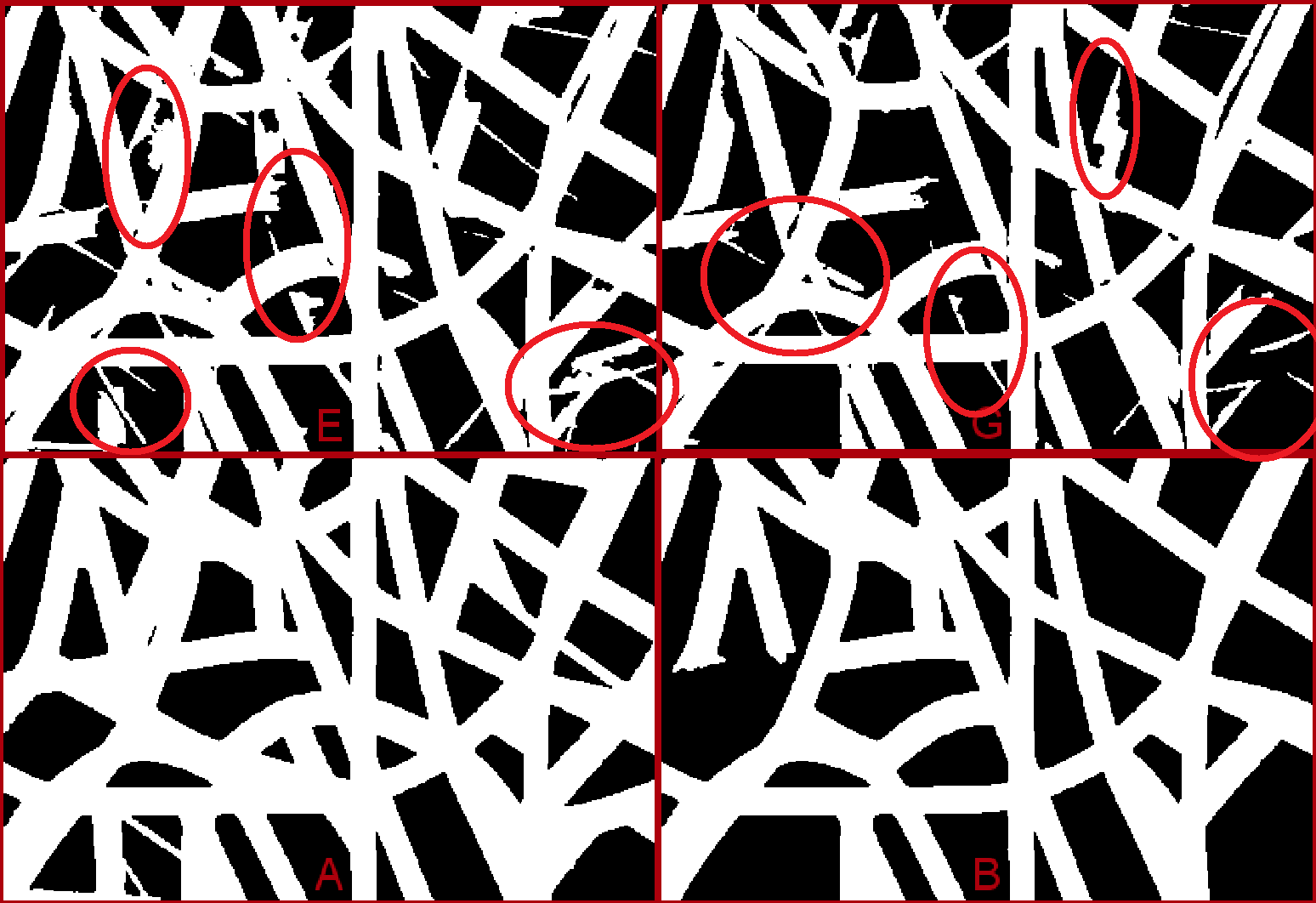


**Figure 7: Partial fibers in segmentation.**

1. Often when imaging fibers in SEM, fibers will appear slightly darker near intersections, which causes segmentation algorithms to segment out holes at intersections. Figure 6F shows a clear example of this phenomenon, which can also be seen more closely in Figure 8. Images where these holes are prevalent should not be used for analysis or should be manually corrected before they are analyzed.


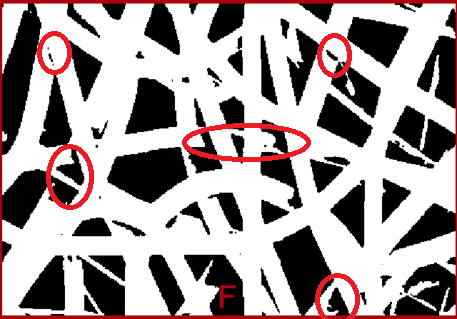


**Figure 8: Intersection holes in a segmented image.**

1. Occasionally, background signal or features of fibers will be incorporated into the segmentation and thus the segmented area will be a combination of fibers and non-fiber elements of the image. Figure 9 shows an example where this occurred in all segmented images. Images where background signal and/or non-fiber features are incorporated into the segmented fibers should not be used for analysis or should be manually corrected before they are analyzed.


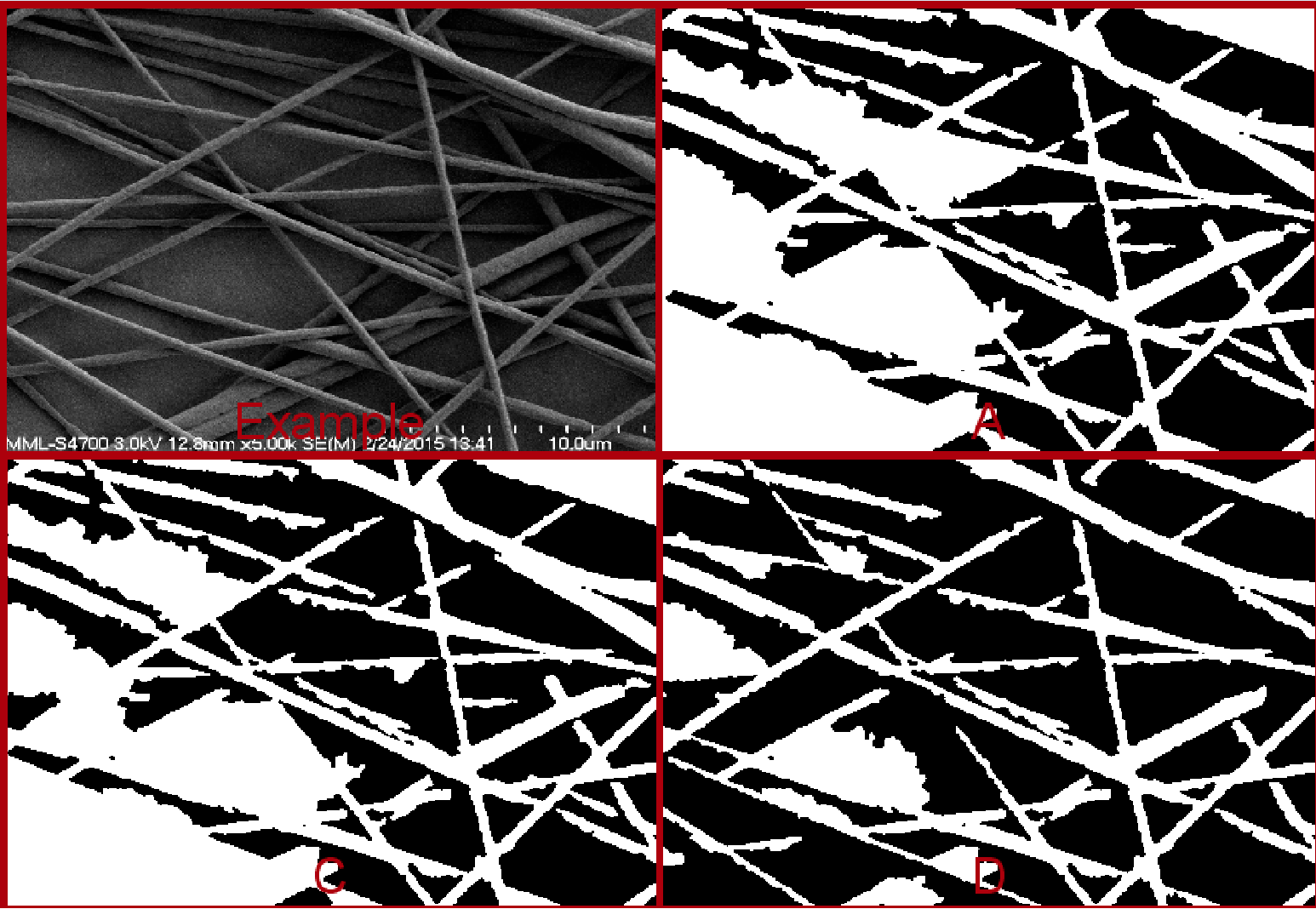


**Figure 9: Background incorporation into segmented fibers.**

1. Sometimes images that have fibers that intertwine, overlap or run parallel to each other will segment two fibers into a single larger fiber. Figure 10 highlights this issue via the red circles, and there are several examples of this occurring in the image A in Figure 6.
   1. Fiber overlap is almost unavoidable in images and thus this problem is prevalent in all samples that are densely spun. Thus, users should select images where this happens as infrequently as possible.


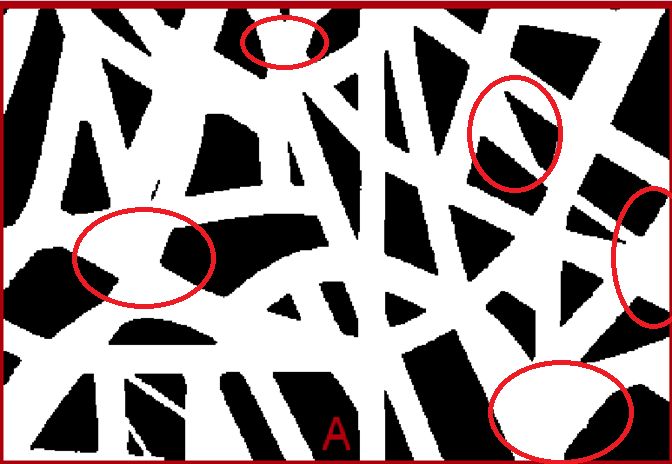


**Figure 10: Overlapping fibers that artifically enlarge fiber diameters.**

1. Finally, if several segmented images contain equally low amounts of the above undesired characteristics (i.e., the fibers in the images segmented well), then users should select the image with the most fibers in it. For example, from Figure 6, images B, C, and D (also shown in Figure 11 below) all contain low amounts of partial fibers, intersection holes, artifacts, and intertwined fibers. Thus, out of these three segmented images, B and C contain the most fibers, and either B or C could be selected by the user as the “best” segmentation.


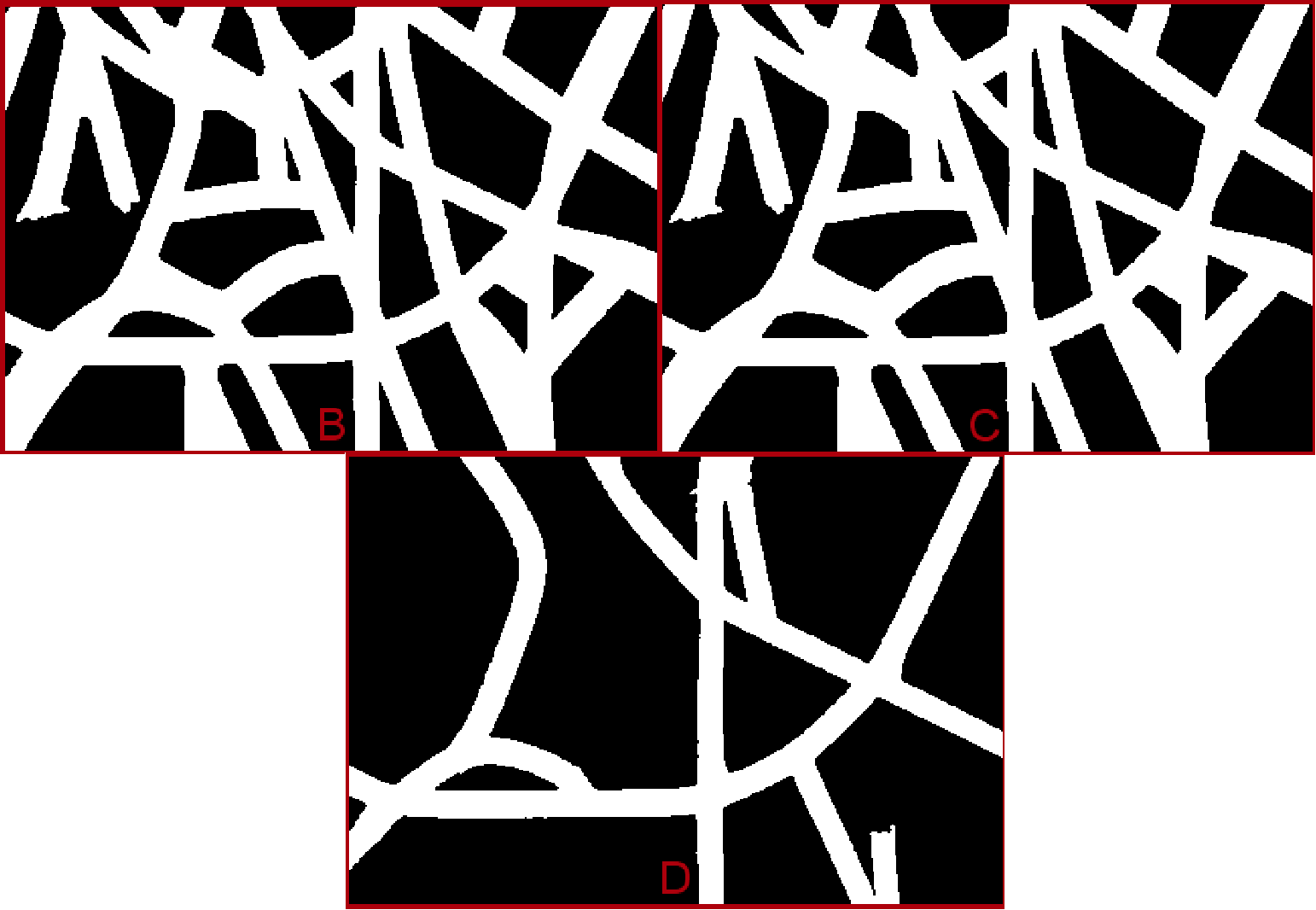


**Figure 11: Choosing the segmented image with the most fibers in it.**

1. The 1. above criteria assumes that at least one of the 16 segmentation algorithms will segment your image well. However, this is not always the case. After gaining some experience in choosing the best segmentation, you’ll be trained on how to manually correct segmentations in your images if no auto segmentation algorithm works well.

1. In summary, Figure 12 below shows an original SEM image with the following segmentation examples: what is considered “ideal” for segmentation of that image, what is considered “good” and will still get the user within 5% of the known answer , what is considered “poor” and produces over 10% error, and what is considered very poor and produces results with over 30% error.


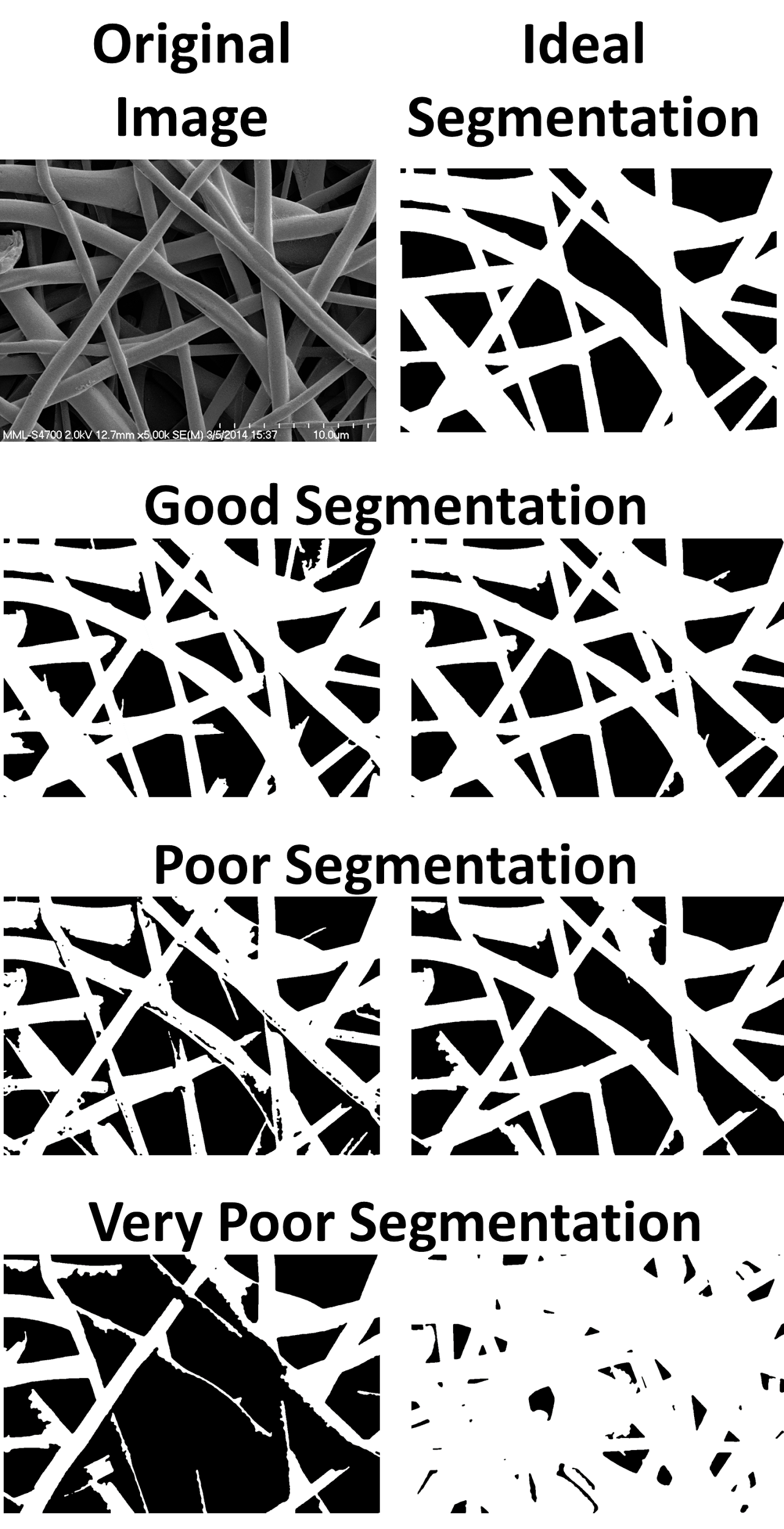


**Figure 12: Comparison of segmentation quality of fiber images.**
