# Supplementary material for: Training to Improve Precision and Accuracy in the Measurement of Fiber Morphology
Source: PLoS One. 2016 Dec 1;11(12):e0167664. doi: 10.1371/journal.pone.0167664 (PMC5132175; doi:10.1371/journal.pone.0167664)
Supplement: S6 File — (DOC) [file pone.0167664.s006.doc]

# **Manual Segmentation and Pixel to Unit Conversion**

## Introduction:

Occasionally, none of the 16 default segmentation algorithms will do an adequate job of segmenting an image into foreground/background. This can occur for a variety of reasons and does not mean that an image is not able to be analyzed by DiameterJ. Users have three main options if the default segmentation algorithms do not segment the image well:

1. Take the image that segmented the best with the default algorithms and manually edit the image until it is representative of original image.
2. Use another segmentation algorithm that is included in ImageJ, or can be found on the internet as a plugin for ImageJ, and segment the image independently of DiameterJ.
3. Develop a new segmentation algorithm/process.

Options 2 and 3 will not be extensively covered here. However, if you are interested below are links to several tools that are popular in the ImageJ community:

- [Trainable Weka Segmentation](http://fiji.sc/Trainable_Weka_Segmentation) machine learning segmentation implementation
- [Canny Edge Detection](http://rsbweb.nih.gov/ij/plugins/canny/index.html) and then fill the resulting image outline in with black
- [Auto local threshold](http://fiji.sc/Auto_Local_Threshold) (in ImageJ/FIJI at Image-->Adjust-->Auto Local Threshold)
- [Auto threshold](http://fiji.sc/Auto_Threshold) (in ImageJ/FIJI at Image-->Adjust-->Local Threshold)
- [Develop your own algorithm!](http://fiji.sc/Introduction_into_Developing_Plugins)

## Manual Segmentation

Manual segmentation of images should be done only when the default algorithms do not produce an image with low error. It is generally easier to start with an image that has already been segmented and then manually correct the mistakes in the image with the below protocol than it is to start with a completely un-segmented image. The steps below outline the procedure for manually segmenting an image if the user starts with a poorly segmented image and edits it.

1. Open ImageJ
2. Open the original image that has not been segmented or cropped.
3. Open the best segmentation of the original image.
4. Below, in Figure 1, is an example image and the “best” segmentation of that image. The rest of this review will follow the steps necessary for editing the segmented image so that it closely resembles that found in the original image.
   1. *Remember, not all fibers need to be included in the segmentation for it to be a “good” segmentation. The fibers just need to be without significant holes, edge defects, or too densely packed for DiameterJ to analyze them.*


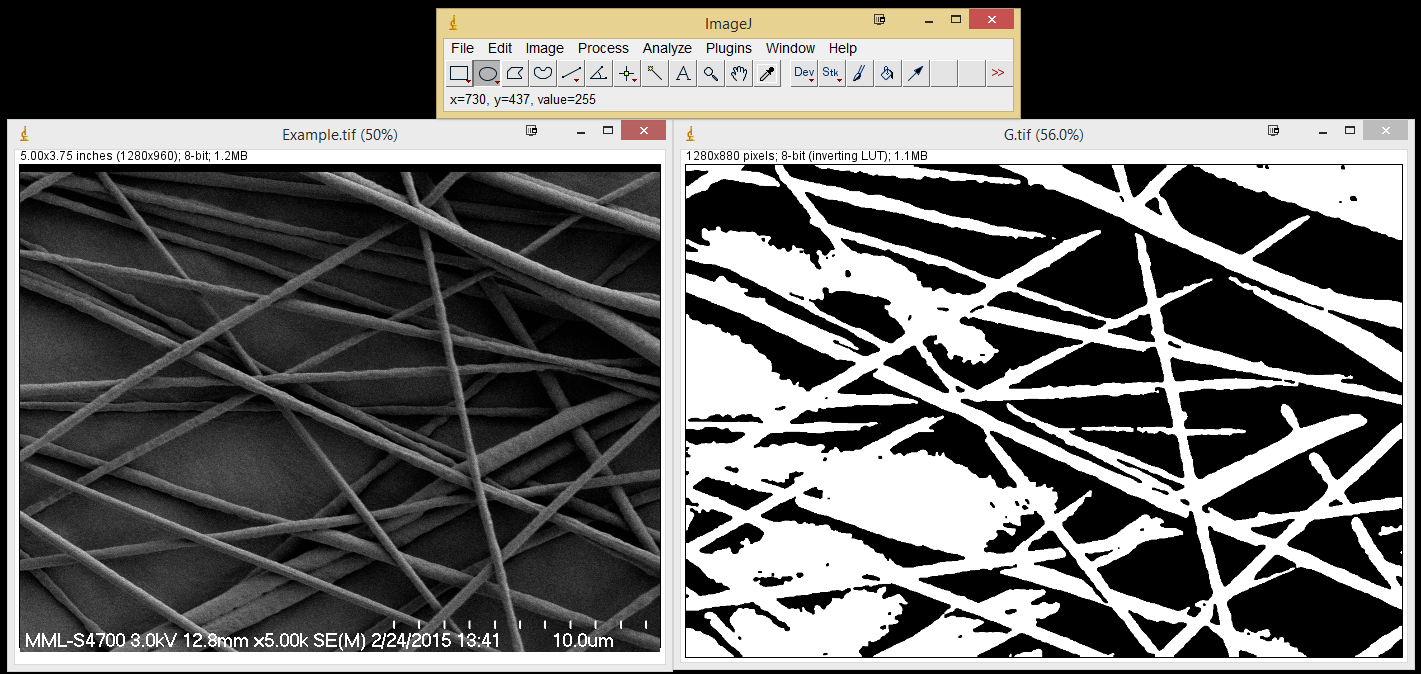


**Figure 1: Original image and “best” segmented image from the segmetnation algorithms.**

1. Click the segmented image and then go to Image → Overlay → Add Image
   1. **This step is critical, simple but critical, to the process below not giving an error**


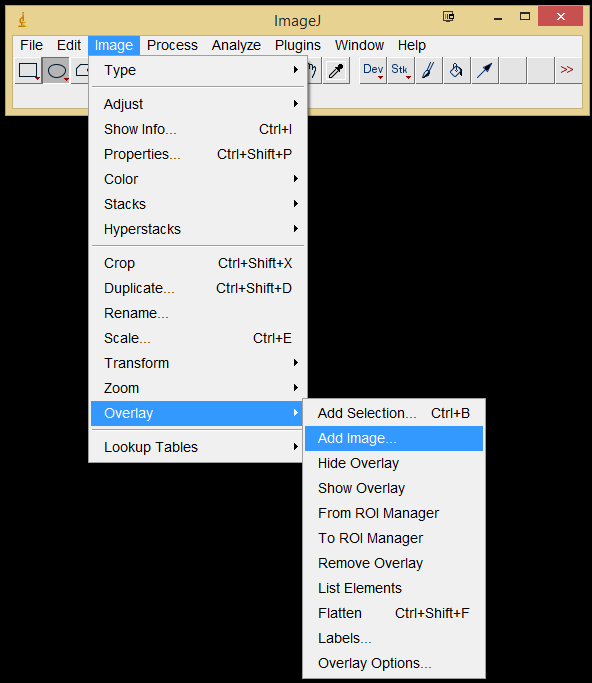


**Figure 2: Overlaying an image menu**

1. A pop-up window will appear asking for which image to add to the segmented image, i.e. be overlayed on top of the segmented image, and the location of the overlay. Figure 3 shows this window.


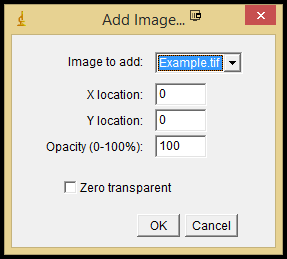


**Figure 3: Pop-up menu for image overlay**

1. From the drop-down box select the image that is not segmented. In this case that image is called “Example.tif”


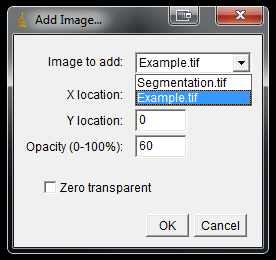


**Figure 4: Choosing the non-segmented image**

1. Next, change the opacity from 100 percent to somewhere between 50 and 75 percent. The level chosen doesn’t affect the outcome and it is up to personal preference. In the example shown in Figure 4 opacity has been set to 60 percent.
2. The original image is now overlayed on top of the segmented image. Figure 5 shows what the overlayed image looks like for the example shown in Figure 1.


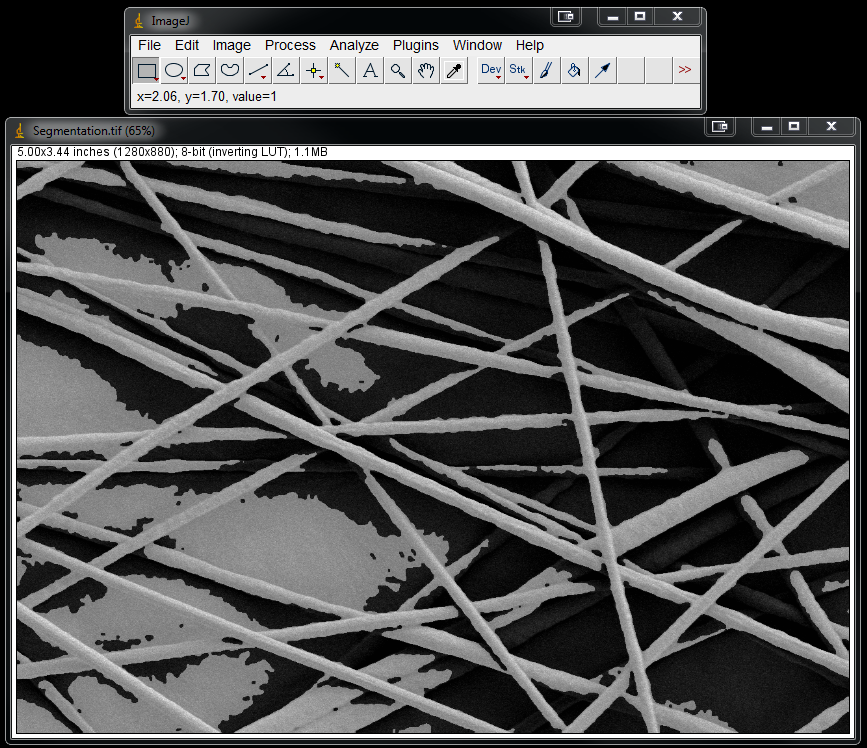


**Figure 5: Image overlay**

1. Zoom in on the image until it is easy for you to see fiber borders and intersections. To zoom the user can either press the “+” key or go to the menu: Image → Zoom → In (+).
   1. To navigate around the zoomed image use the hand tool, located in the menu bar, shown in Figure 6.


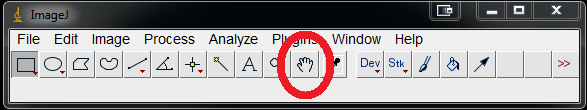


**Figure 6: Hand tool in the menu bar.**

1. Figure 7 shows the upper left hand corner of the image, zoomed to 150%.


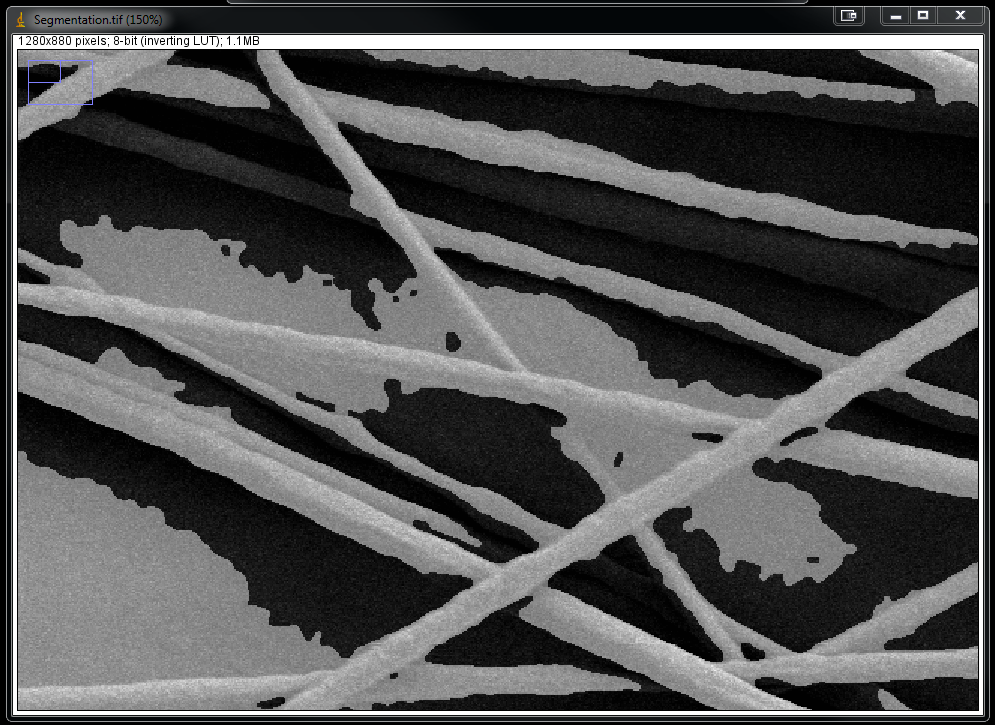


**Figure 7: Zoom of overlayed image**

1. Use the polygon selection tool (circled in Figure 8 to select regions of fibers or background that you would like to fill in. Generally, fill background with black and fill fibers with white. Figure 9 shows a selection of a large area the should be background.
   1. To fill shapes either press cntrl+F or got to Edit → Fill
   2. Shapes drawn with the polygon tool must be closed shapes thus the final point that you create must be in the same location as the first point that you created.
   3. The default color for all fills and shapes in black in ImageJ unless the user changes it. Thus, filling in background pixels is easy with default settings.


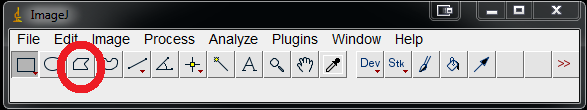


**Figure 8: Polygon selection tool**


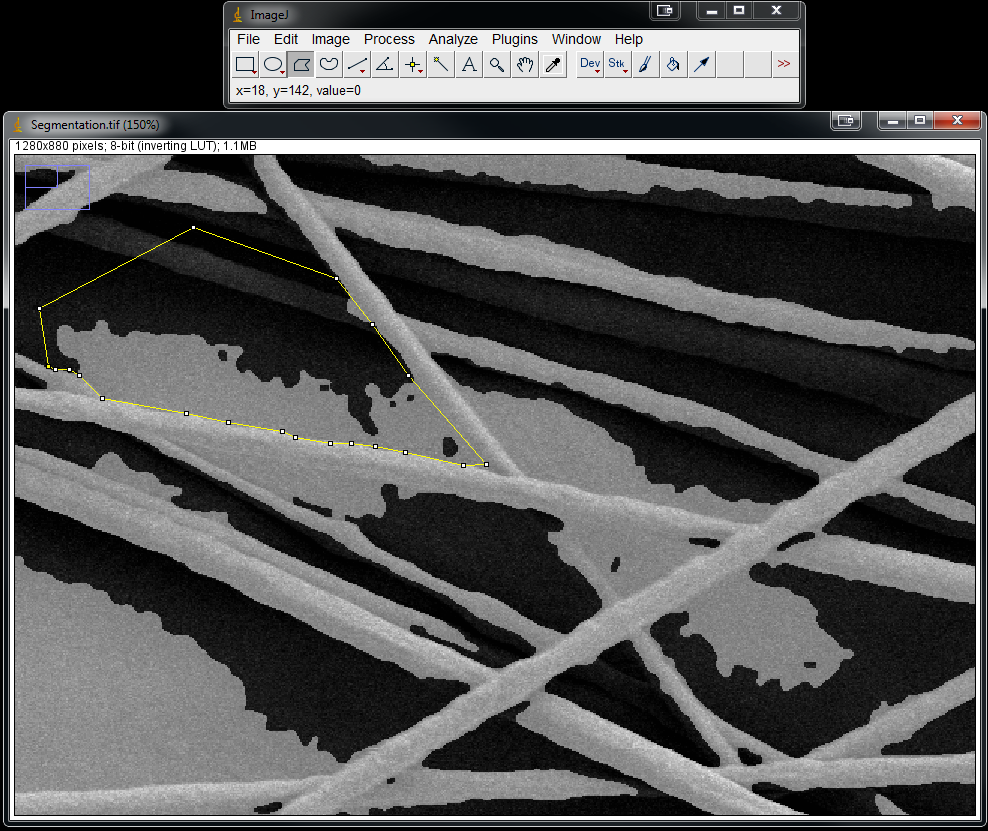


**Figure 9: Polygon selection tool highlighting an area of background. White dots represent mouse clicks and slight inflection points in the polygon. Note that a part of a fiber that was not included in the original segmentation is also being added to the background.**

1. To fill in fibers, and not background, the fill color must be changed. To change the fill color go to Edit → Options → Colors… shown in Figure 10 below.


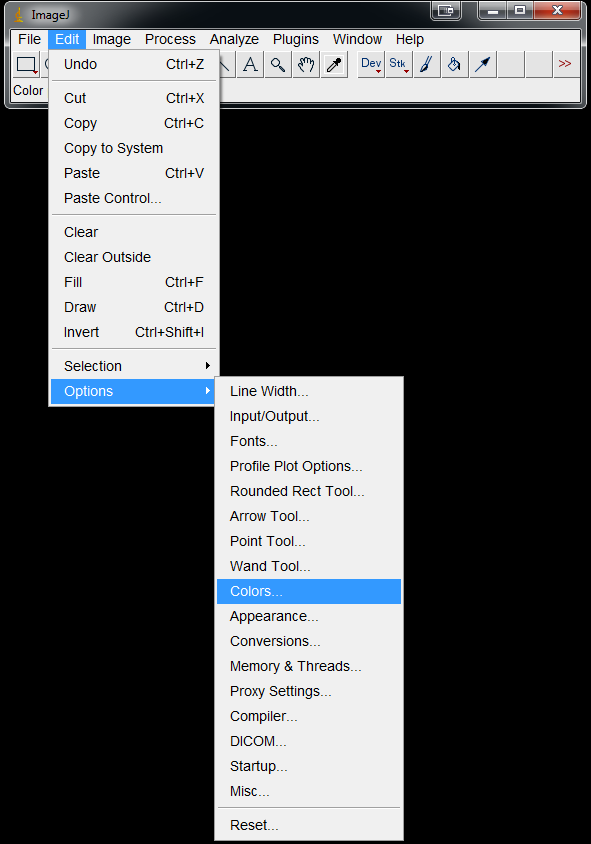


**Figure 10: Changing the fill color**

1. Once selected a pop-up menu will appear showing drop down menus for the “Foreground”, “Background” and “Selection” colors, as shown in Figure 11.


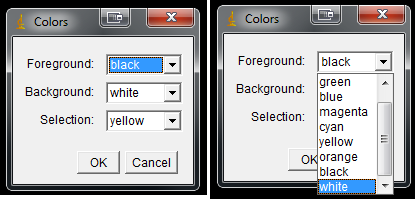


**Figure 11: Color pop-up menu and changing the foreground color.**

1. Change the Foreground color from black to white and click “OK” to save the change.
2. Find a fiber that you would like to add, a section of a fiber that was incorrectly segmented, or a hole in a fiber and carefully outline it with the polygon selection tool. Figure 12 shows a fiber that was not segmented into the image that is now outlined.


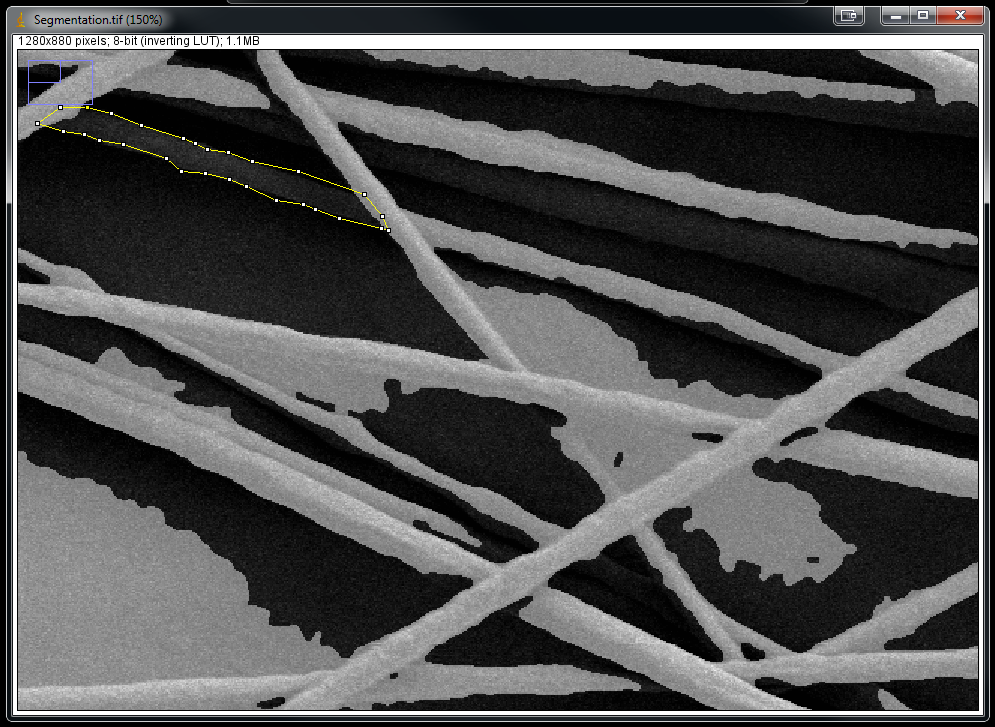


**Figure 12: Polygon selection tool highlighting an area offiber. White dots represent mouse clicks and slight inflection points in the polygon.**

1. Fill the fiber selection in the same way that you filled the background. Figure 13 shows the fiber after filling.


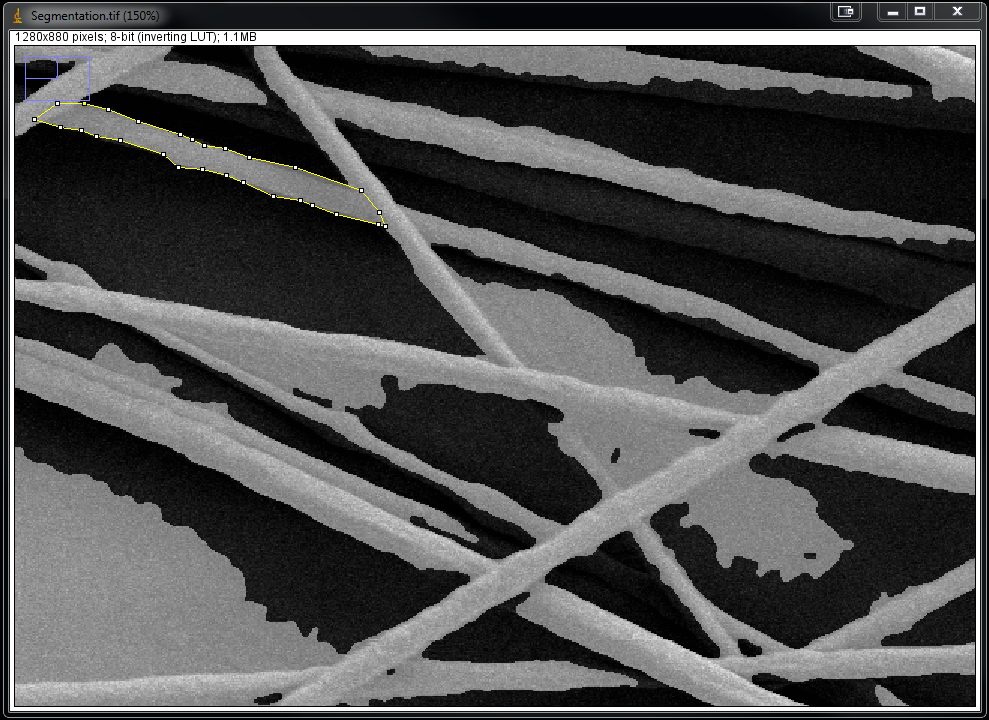


**Figure 13: Filled fiber.**

1. Repeat the background and fiber fill procedures until the entire image has been segmented to the user’s level of satisfaction.
   1. *Remember, not all fibers need to be included in the segmentation for it to be a “good” segmentation. The fibers just need to be without significant holes, edge defects, or too densely packed for DiameterJ to analyze them.*
2. Figure 14 shows the image from Figure 5 after all modifications have been made.


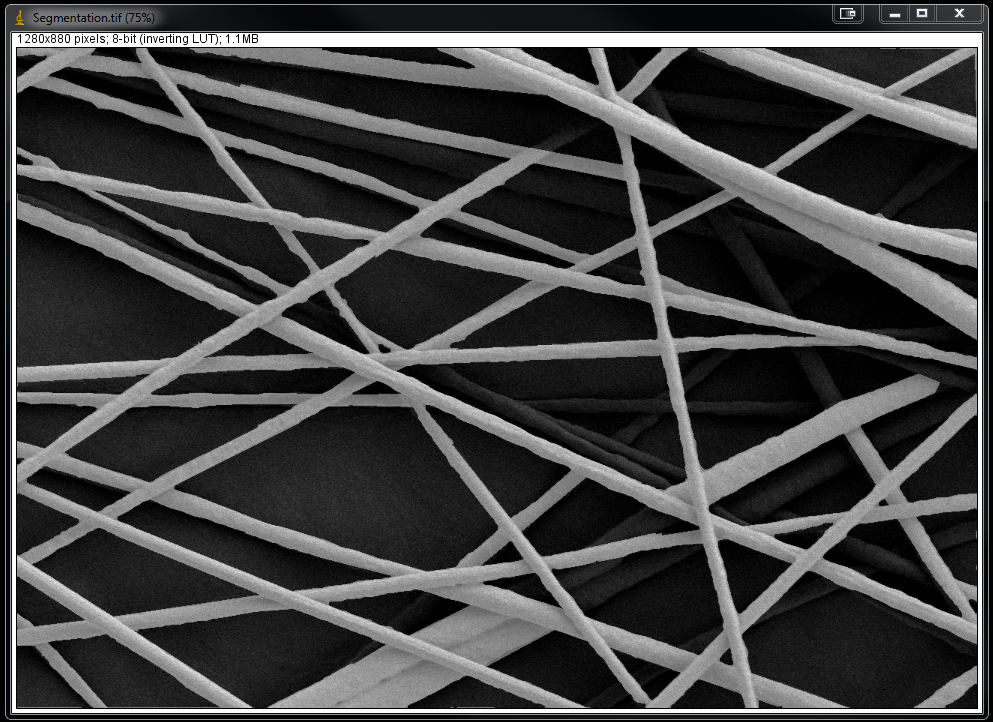


**Figure 14: Segmentation after manual editing.**

1. Once the user is satisfied with the segmentation, go to Image → Overlay → Remove Overlay to remove the overlayed original image. Figure 15 shows the location of the menu and Figure 16 shows the segmented image after the overlay has been removed.


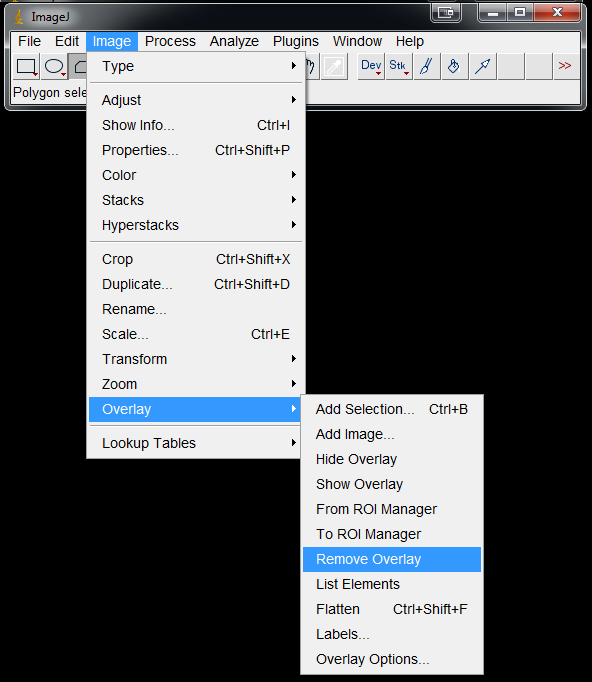


**Figure 15: Menu to remove the overlay from the original segmentation**


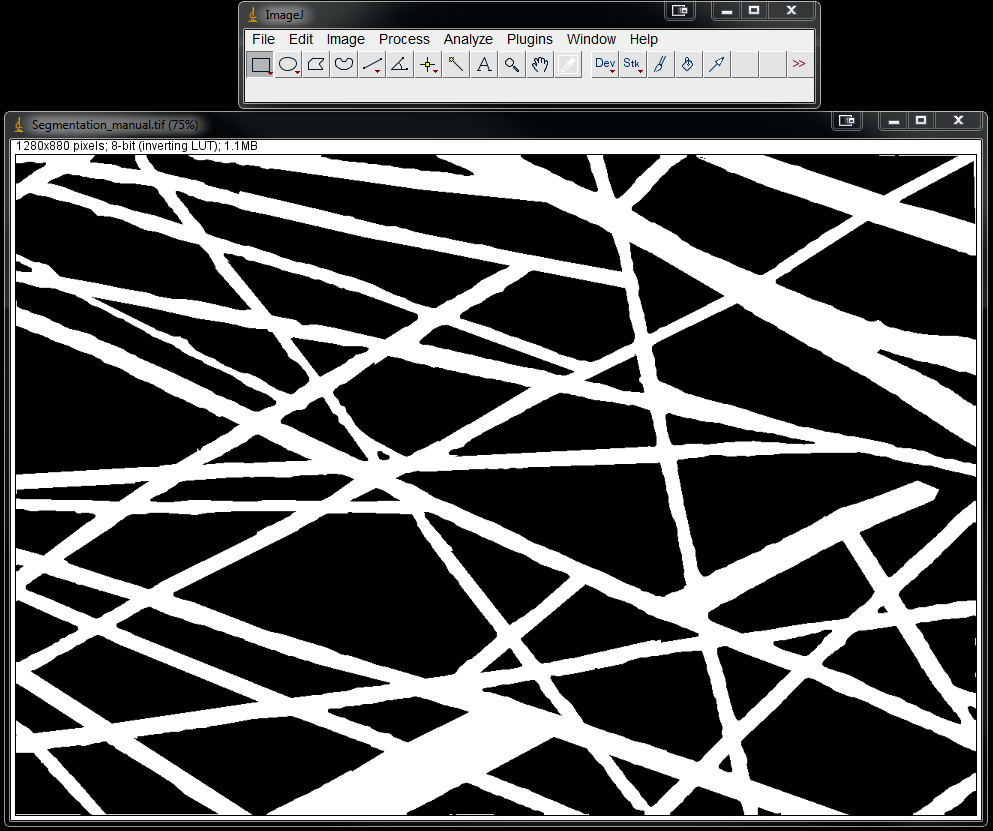


**Figure 16: Segmented image after overlay has been removed.**

1. To insure that the image has not had an artifacts added to it unintentionally users should the force the image to return to a binary state by going to Process → Binary → Make Binary. The menu can be seen in Figure 17 below.


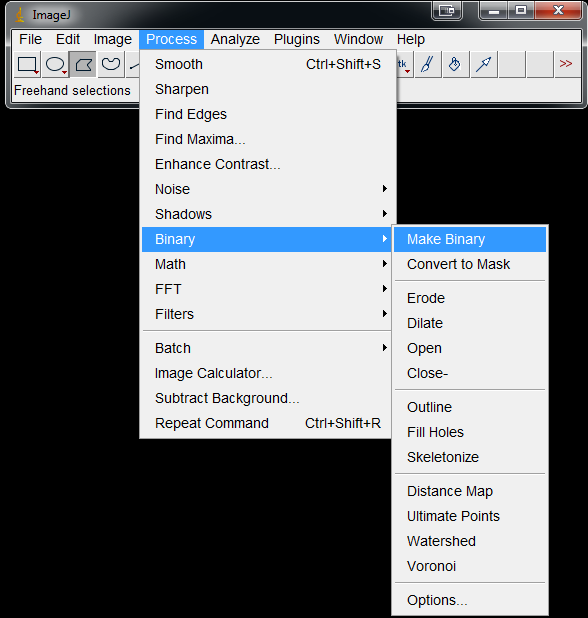


**Figure 17: Binary Image menu.**

1. Often when changing an image to binary the image will invert the black and white pixels so that the fibers are black and the background is white. Figure 18 shows an example of this occurring.


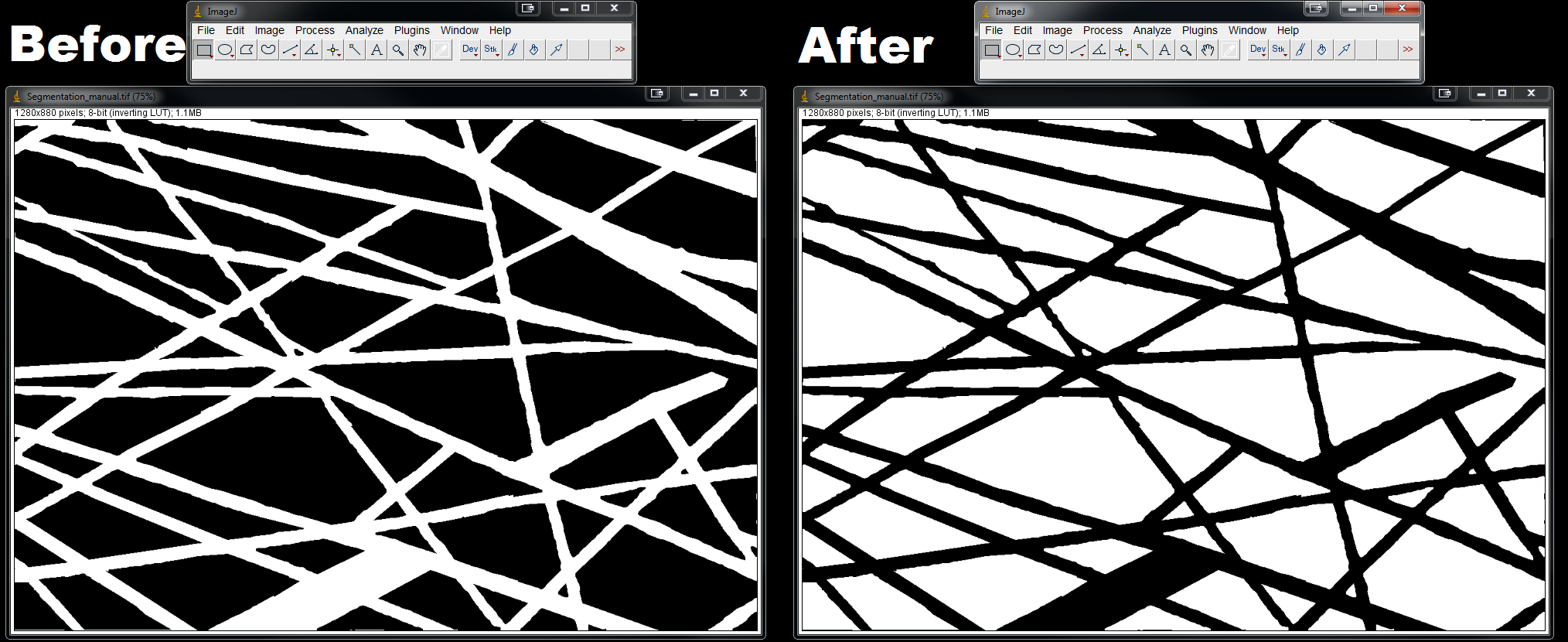


**Figure 18: A segmented image before and after executing the “Make Binary Command”.**

1. If this occurs simply go to Edit → Invert or press “cntrl+shift+i” to invert the image. Figure 19 below shows the Invert menu.


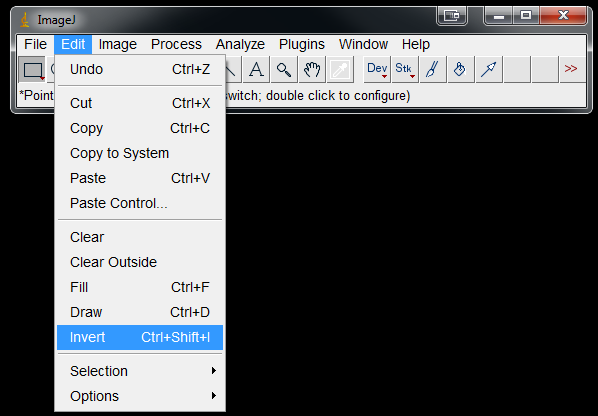


**Figure 19: Invert menu.**

1. Save the image as a TIF image in the “best segmentations” folder and analyze this new image with DiameterJ using the standard procedure outlined in the “Installation and User Instructions” document.

# **Pixel to Unit Distance Transformation**

***All measures given by DiameterJ are in pixels by default***

Pixel units are not very informative and should be converted to real units whenever analyzing an image with a known magnification. Please go to “[Installation and User Instructions](https://docs.google.com/document/d/1lODEj02e2eFtQ-v0B3TJd53Q2XFZZW2i0szrfyX0kRY/edit?usp=sharing)” to learn how to do this conversion.
