# Supplementary material for: Training to Improve Precision and Accuracy in the Measurement of Fiber Morphology
Source: PLoS One. 2016 Dec 1;11(12):e0167664. doi: 10.1371/journal.pone.0167664 (PMC5132175; doi:10.1371/journal.pone.0167664)
Supplement: S7 File — (DOCX) [file pone.0167664.s007.docx]

# **DiameterJ Fiber Diameter Analysis**

## Introduction:

In this training participants will carefully analyze the output of DiameterJ from six SEM micrographs of real PLGA, *poly(lactic-co-glycolic acid*), electrospun fibers. Please download the zip file below. In it are the six original SEM images, the six “best” segmentations of these images, and a file explicitly stating the pixel to unit distance conversion.

Zip file download - <https://goo.gl/Ncin8D>

Unzip the files to any location and analyze the “best segmentations” folder with the DiameterJ plugin. The below instructions will review the exact steps for analyzing these images and are meant to help the user understand the general process for analyzing any set of images.

The user should not alter the best segmentation images before analyzing them as the results from this analysis will be used in the training “Protocol for the Analysis of the Output of DiameterJ - 3/4 Quiz.”

## Image Analysis

1. Go to ImageJ → Plugins → DiameterJ → DiameterJ 1.00X shown in Figure 1 below.


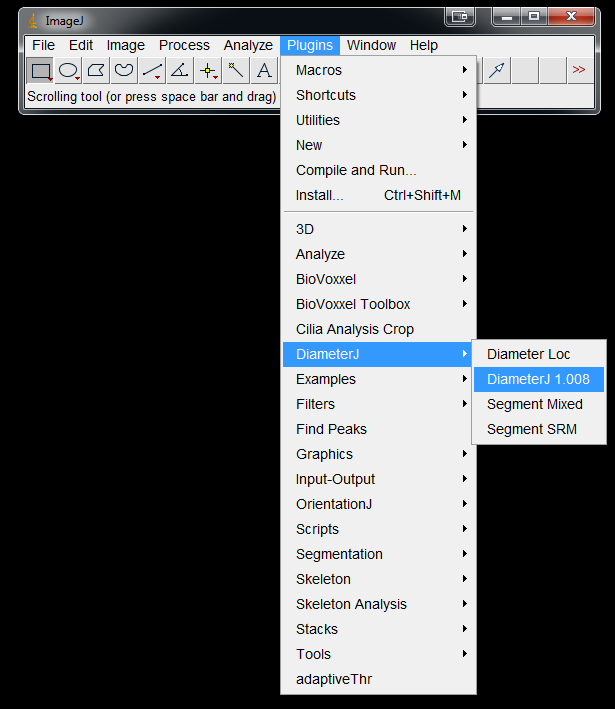


**Figure 1: Menu for DiameterJ’s analysis algorithm**

1. A pop-up window will appear, navigate to the directory where you saved the “Best Segmentation” images and click select. Figure 2 below shows the pop-up window after navigating to the “Best Segmentation” folder on an example computer.


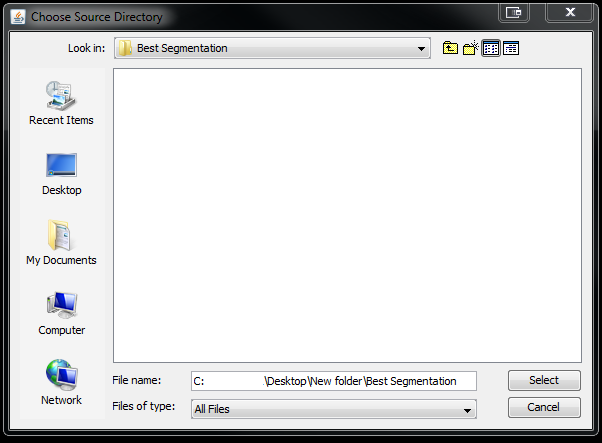


**Figure 2: Pop-up window after navigating to the “Best Segmentation” directory**

1. A series of windows will open and close and all .tif images in the “Best Segmentations” folder will be analyzed. Once completed, the “log” window will say the total number of files analyzed and the amount of time it took DiameterJ to analyze those images. Figure 3 below shows the final log window.


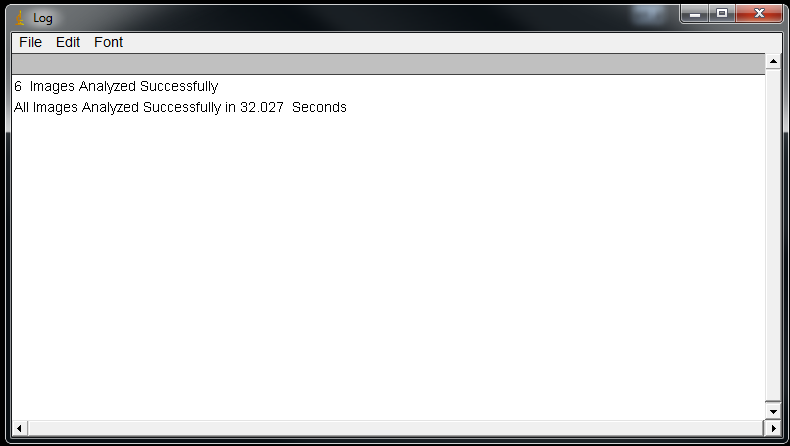


**Figure 3: Log output after DiameterJ has completed.**

1. Three folders will be created in the same folder that the segmented images are located. These three files are called “Diameter Analysis Images”, “Histograms”, and “Summaries”. Figure 4 shows the “Best Segmentations” folder after DiameterJ has completed its analysis.


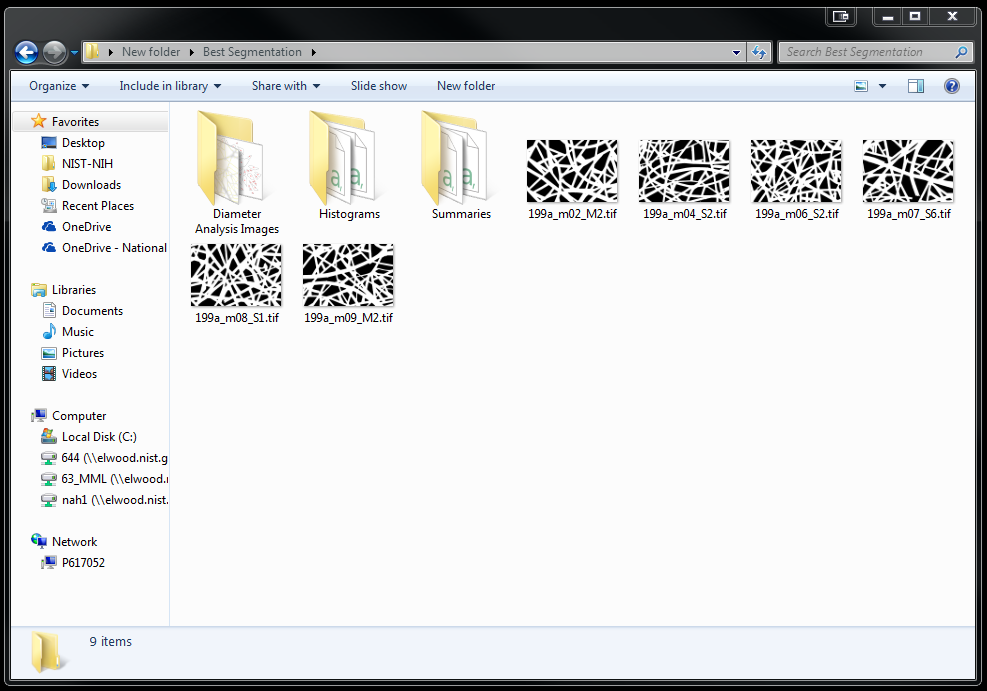


**Figure 4: Best segmentations folder after DiameterJ analysis.**

1. The first step after DiameterJ has completed its analysis is to review the images located in the “Diameter Analysis Images” folder to make sure that the fibers that were measured and the centerlines of the fibers that were measured correspond to the original image.
2. The first image to check should be a montage image (XX_Compare.png) of the segmented image. The montage image contains the segmented image, a centerline of the fibers, an overlay showing the euclidean distance transform of the fibers with a yellow centerline overlayed on top of it, and the pores that DiameterJ analyzed from the image. Figure 5 shows the montage image for file “199a_m02_M2.tif”.


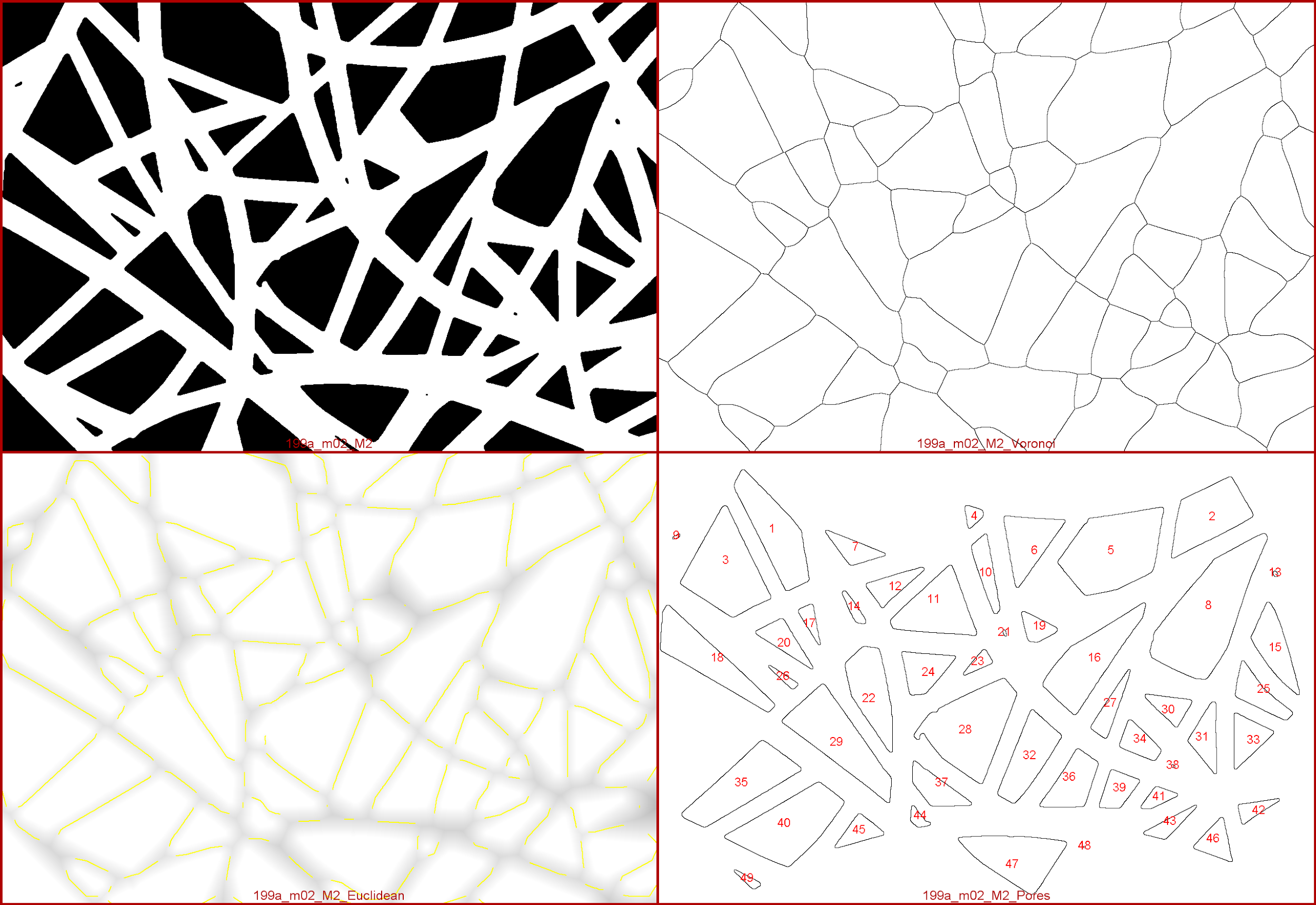


**Figure 5: Montage image produced by DiameterJ of an image.**

1. The yellow centerline overlaid on the euclidean distance transform represents the location on the fibers where DiameterJ measured the fibers to obtain its histogram of fiber radii. When examining the montage image make sure that the centerlines follow the fibers and that the pores are the pores that appear in the image. If they are not, then DiameterJ has inverted the fibers and pore space and the user should invert their segmentation and re-run DiameterJ.
   1. Of note is that fiber intersections have no yellow line overlaid on them because DiameterJ does not measure areas where fibers overlap due to inaccuracies in these regions. Additionally, not all pores show up in the pores image because DiameterJ does not analyze pores that touch the edge of the image.
2. If all centerlines and pores appear to be consistent with the segmented image then the user can go on to analyzing the other outputs of DiameterJ. If they do not, then either the user needs to select a different segmentation, further edit the current segmentation, or re-image their sample.

The next steps in this tutorial involve assessing nanofiber diameter.

1. There are two different approaches that DiameterJ uses to determine fiber diameter. An algorithm known as the “Super Pixel” algorithm and the “Histogram” output. The super pixel algorithm is explained in detail [here](http://imagej.net/DiameterJ#Super_Pixel_Diameter). The histogram is the average value of the Gaussian fit of the histogram found in the “XX__Histogram.csv” file. Generally, the histogram average is most accurate when multiple fiber diameters are not present in an image and the Super Pixel average is the most accurate when multiple fiber diameters are present.
2. Check the file named “XX_Total Summary.csv” in the folder named “Summaries” to see the total image averages of diameter. Figure 6 shows the output in the “_Total Summary.csv” file.
   1. These values can be easily biased if the diameter histogram analyzed has a non-normal distribution (i.e. multiple peaks, significant noise leading or trailing to/from the peak, etc.).

| **Diameter_Metrics** | **Diameter_Values** | **_** | **Other_Metrics** | **Values** |
| --- | --- | --- | --- | --- |
| Super Pixel | 36.3854 |  | Mean Pore Area | 7181.8571 |
| Histogram Mean | 36.6629 |  | Pore Area SD | 7382.116 |
| Histogram SD | 8.9639 |  | Min. Pore Area | 12 |
| Histogram Mode | 34 |  | Max. Pore Area | 30015 |
| Histogram Median | 36 |  | Percent Porosity | 0.4582 |
| Histogram Min Diam. | 2 |  | Number of Pores | 49 |
| Histogram Max Diam. | 88 |  | Intersection Density (100x100px) | 1.0298 |
| Histogram Integrated Density | 173723 |  | Characteristic Length | 153.4871 |
| Histogram Raw Integrated Density | 173723 |  |  |  |
| Diameter Skewness | 0.1122 |  |  |  |
| Diameter Kurtosis | 1.1743 |  |  |  |
| Fiber Length | 9218 |  |  |  |

**Figure 6: Total Summary Output file.**

1. To determine if a radius distribution is non-normal look first at the “XX__Radius Histogram.tif” file in the “Histograms” folder. If the histogram has multiple peaks then the distribution is non-normal and you should do more analysis with another piece of software. In steps 14-35 below we will discus how to perform this analysis.
   1. An example of a histogram with a single peak (Figure 7A) and with multiple peaks (Figure 7B) can be seen below.


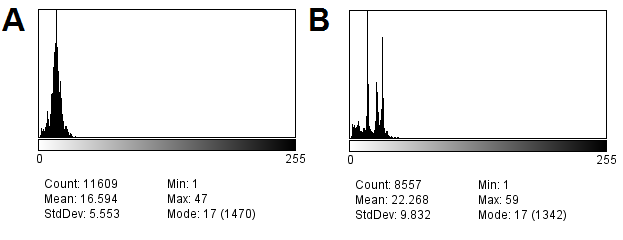


**Figure 7: Histograms of fiber radii from “XX_Radius Histogram.tif” files. (A) A histogram with only one peak present. (B) A histogram with multiple peaks present.**

1. If only one peak is present the next file to check to see if your data is sufficiently normal is the “_Total Summary.csv” file located in the “Summaries” folder. In this file check the “Diameter Skewness” and “Diameter Kurtosis” values. Generally, if both Skewness and Kurtosis are are between -1 and 1 then your diameter distribution is sufficiently normal to use the “Histogram Mean” and “Histogram SD” values in the summary file.
   1. **If both Skewness and Kurtosis are not between -1 and 1 it is strongly recommend that you perform a peak fit of the diameter histogram to obtain a more accurate measure of the mean radius**. Figure 7b shows an example fiber diameter histogram that has a non-normal distribution and that should be analyzed with further peak fitting.
   2. It is possible to get skewness and kurtosis values between -1 and 1 with multiple peaks if those peaks are symmetric. Thus, the skewness and kurtosis values indicating a normal value is only relevant if 1 large peak is shown in the histogram.
2. Peak fitting has been found to produce the most consistently accurate results when analyzing images of real samples and thus, the remainder of the analysis of the fiber diameter data will be focused on peak fitting of the histogram data.
3. Multi-mode peak fitting can be performed with almost any scientific analysis software. However, the vast majority of these pieces of software are not open source and available to the community. There are two prevalent, open source, tools that can be used for accurate and validatable peak fitting of histogram data, called R and Python. These tools are actually programming languages and have extensive documentation available online. However, in order to peak fit with these pieces of software an in-depth knowledge of how to program in these languages is necessary. This is outside of the scope of this tutorial. Thus, another piece of software will be outlined in this tutorial:
   1. Fityk 0.9.8 for Windows and Linux
      1. If you run OSX (any version) you will need to use a virtual machine to run this software.
4. This piece of software is not required for peak fitting and Fityk is in no way recommended above any other pieces of software. It has been chosen for this training only because it is open source and available freely to the community. (Versions after 0.9.8 are not free or open source but this version is sufficient for the work you will need to do)
   1. To download Fityk for Windows - <https://github.com/wojdyr/fityk/downloads>
   2. To install Fityk for Linux - <https://launchpad.net/~wojdyr/+archive/ubuntu/fityk>
5. Install Fityk with the recommended settings and open the program after it has completed the installation.
6. Once opened go to Data → Load File menu to open your histogram. Figure 8 shows the Fityk window after opening with the Data → Load File menu.


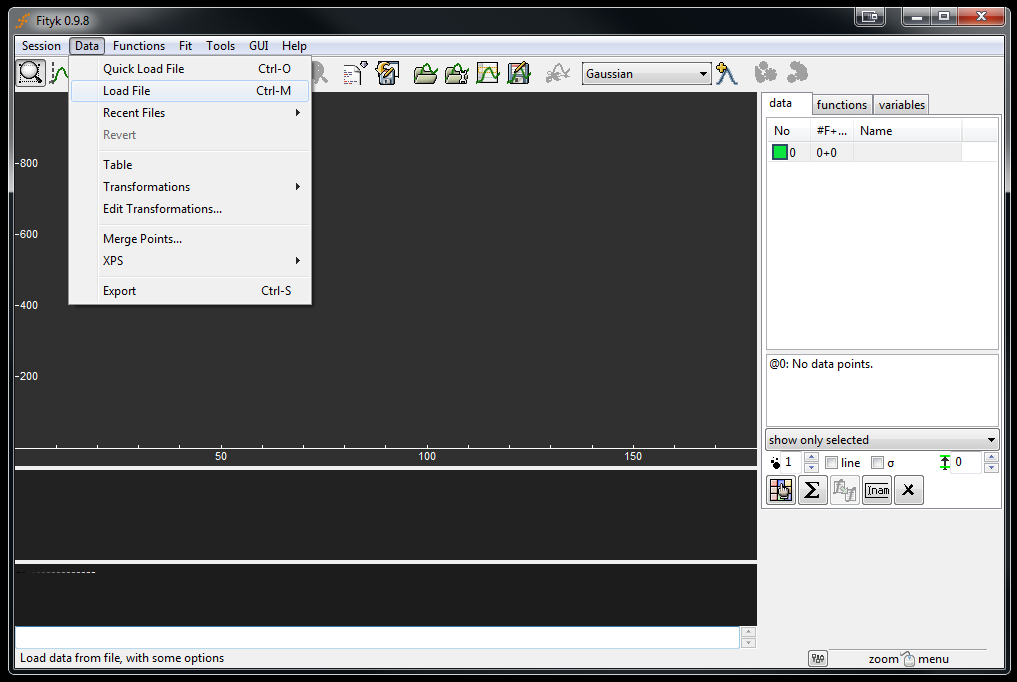


**Figure 8: Fityk after installation and the Data menu open**.

1. Once you have clicked the “Load File” drop down menu a pop-up window will appear asking you to navigate to the file you would like to load. Navigate to the location where the “Histograms” file has been created. Click any of the “XX_Histogram.csv” files present in this folder. By default Fityk loads the first column as the X of your histogram and the second column as your y values. It then shows a preview of what your histogram looks like. Figure 9 shows a screenshot of the pop-up window with the x and y columns circled in red and the preview of the histogram also circled in red.


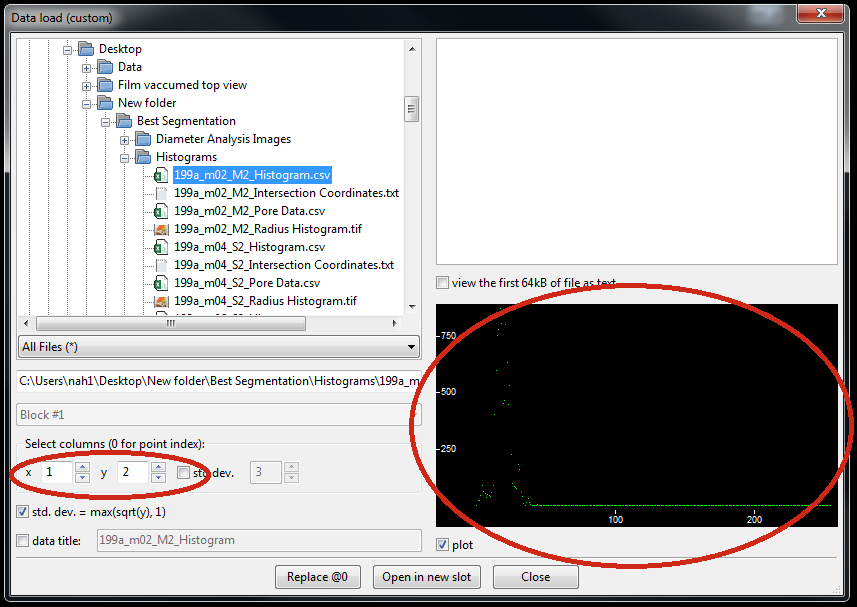


**Figure 9: Load file menu of Fityk.**

1. The preview of the histogram should look similar in shape to the histogram shown in the “XX_Radius Histogram.tif” file.
2. After selecting the file you would like to peak fit click the “Replace @0” button at the bottom of the window and then click “Close”. The histogram file should now be loaded and ready to be fit with a curve.
3. In this tutorial we will cover Gaussian Peak fitting of histograms because these curves have the easiest to interpret mean, standard deviation, etc. However, Fityk allows for using a variety of curves. If your data clearly shows non-normal distributions the user should use other curves instead.
   1. In practice, especially when fitting peaks from cumulative histograms (described further below) the authors have found that 1 of 4 distributions appear to be likely across many samples and spinning conditions 1) Gaussian 2) Log Normal 3) Lorentzian and 4) Pearson
   2. Fitting with each of these curves lowers the residual error of the fit and can reduce the standard deviation reported. However, means tend to change only slightly between different fits of the same data. Thus, in most cases, Gaussian fits are sufficient to determine the average fiber diameter and offer a more conservative (i.e. larger) standard deviation.
4. To select the type of curve to fit to your data click the drop-down menu and select the type of curve you would like to add to your histogram. Figure 10 has the drop-down menu for the type of curve circled.
   1. For this example set the type of curve to Gaussian.


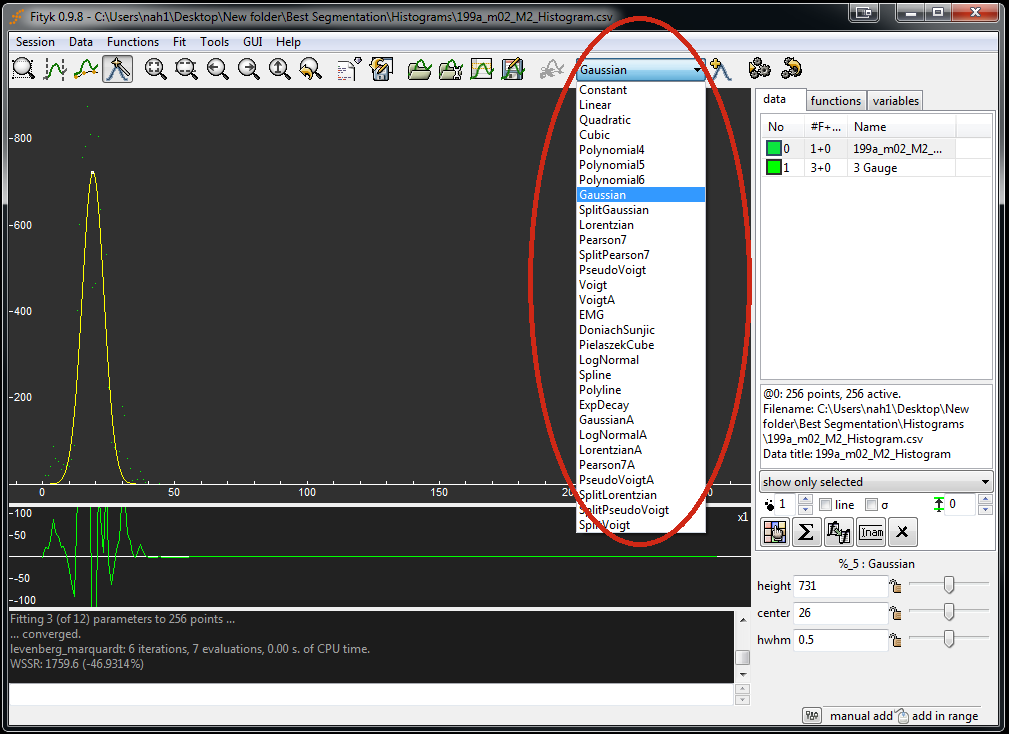


**Figure 10: Setting the type of curve to add to your radius histogram data to Gaussian.**

1. The user can now manually add peak(s) to be “fit” to the data or the user can tell the software to perform its best guess on where to add peak(s). Figure 11A shows the icon to click if the user wants to manually add peaks and Figure 11B shows the icon to click if the user wants to have the computer add a peak automatically.
   1. Often if the histogram has several distinct peaks in it that are very close together and the user wants to resolve the peaks it is better to manually add peaks. If peaks are distinct and separate the auto-peak creator usually does a good job.


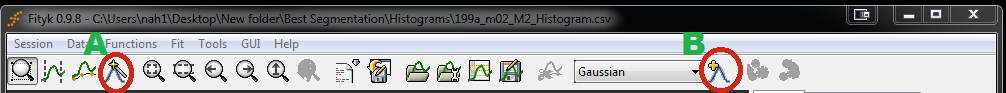


**Figure 11: Location of manual and auto-peak add icons**.

1. To manually add peaks, click the icon circled in Figure 11A, then left-click and hold the cursor at the location you would like the peak to be centered at and move the mouse left or right to broaden the curve.
2. Repeat this process as many times as there are distinct peaks in your data.
3. To auto-add peaks, click the icon circled in Figure 11B. Every time you click this icon another peak will be added to the histogram.
   1. To delete a peak that has been either manually or automatically added, right click the top of the peak and select “delete” from the menu that appears.
4. Figure 12A shows a histogram with only one distinct peak while Figure 12B shows a histogram with multiple peaks added before “fitting” has occurred.


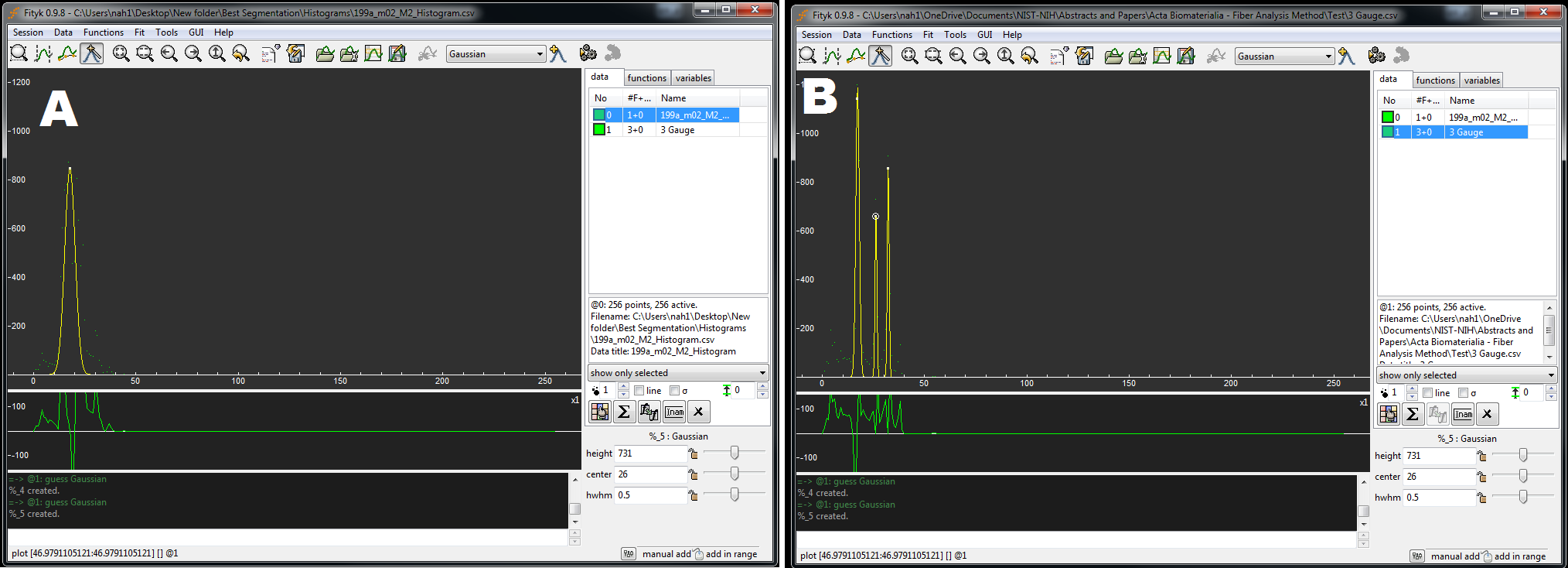


**Figure 12: Two instances of Fityk with data loaded. A) A histogram of fiber radii with only one peak present. B) A histogram of fiber radii with three peaks present.**

1. Once the appropriate number of peaks have been added click the “fit” icon circled in red in Figure 13.


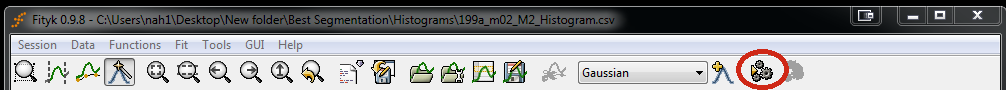


**Figure 13: Location of the “fit” icon in Fityk’s menu bar.**

1. The algorithm will optimize the residual (distance between curve fit and real data) so that the Gaussian curves fit the data with the minimum amount of error possible. Once this process is complete the user can obtain several metrics about the peaks that have been fit. Most importantly a mean, full width half max, and height of the peak fit.
2. To find information about the peak hover the mouse over the top of the peak that was just fit or export peak parameters by going to Functions → Export Peak Parameters (Figure 14). Save the file under any name.
   1. The output of the file is a .peaks file which you can open in any spreadsheet program. Figure 14 shows the location of the “Export Peak Parameters” menu.
   2. By default spreadsheet programs (such as excel) do not “see” the .peaks file. While we do not recommend Excel over any other spreadsheet program, a general fix for this problem using Excel can be extrapolated to other programs. In Excel go to File → Open and then navigate to the location of the .peaks file. Then, next to the “File Name” box select “All Files” from the drop down menu. (this process can be seen in Figure 15 below). The .peaks file should appear. Open this file.
   3. Alternatively, in Windows you can right click the .peaks file and choose “Open With…” and select Excel as the default program to open these files.


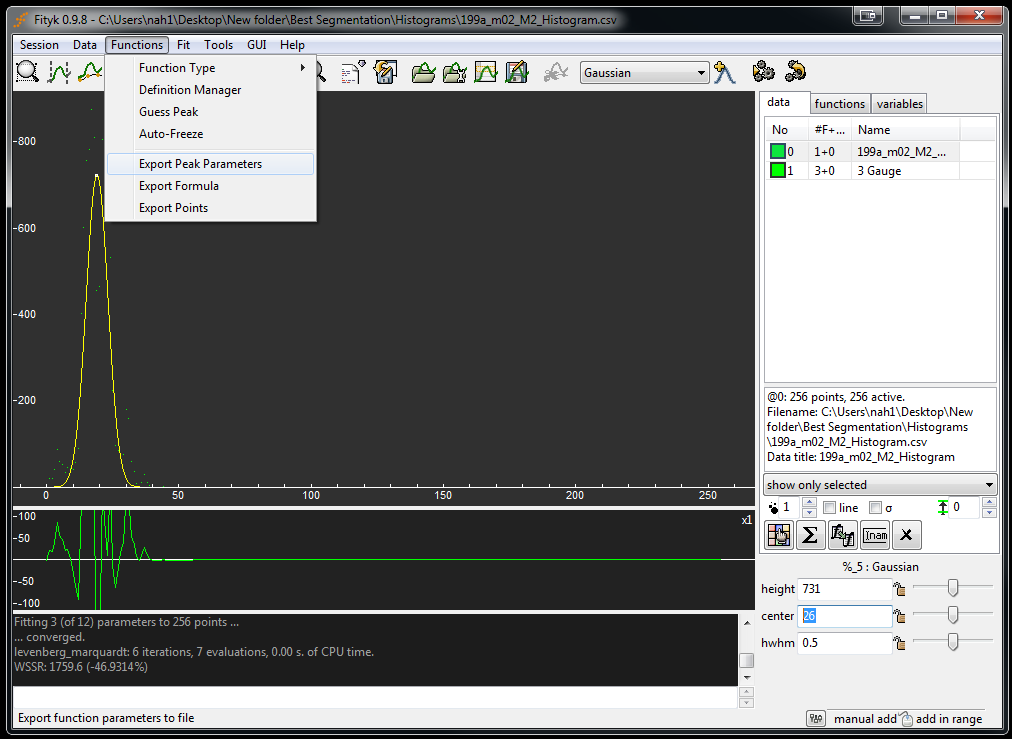


**Figure 14: Location of the “Export Peak Parameter” menu in Fityk.**


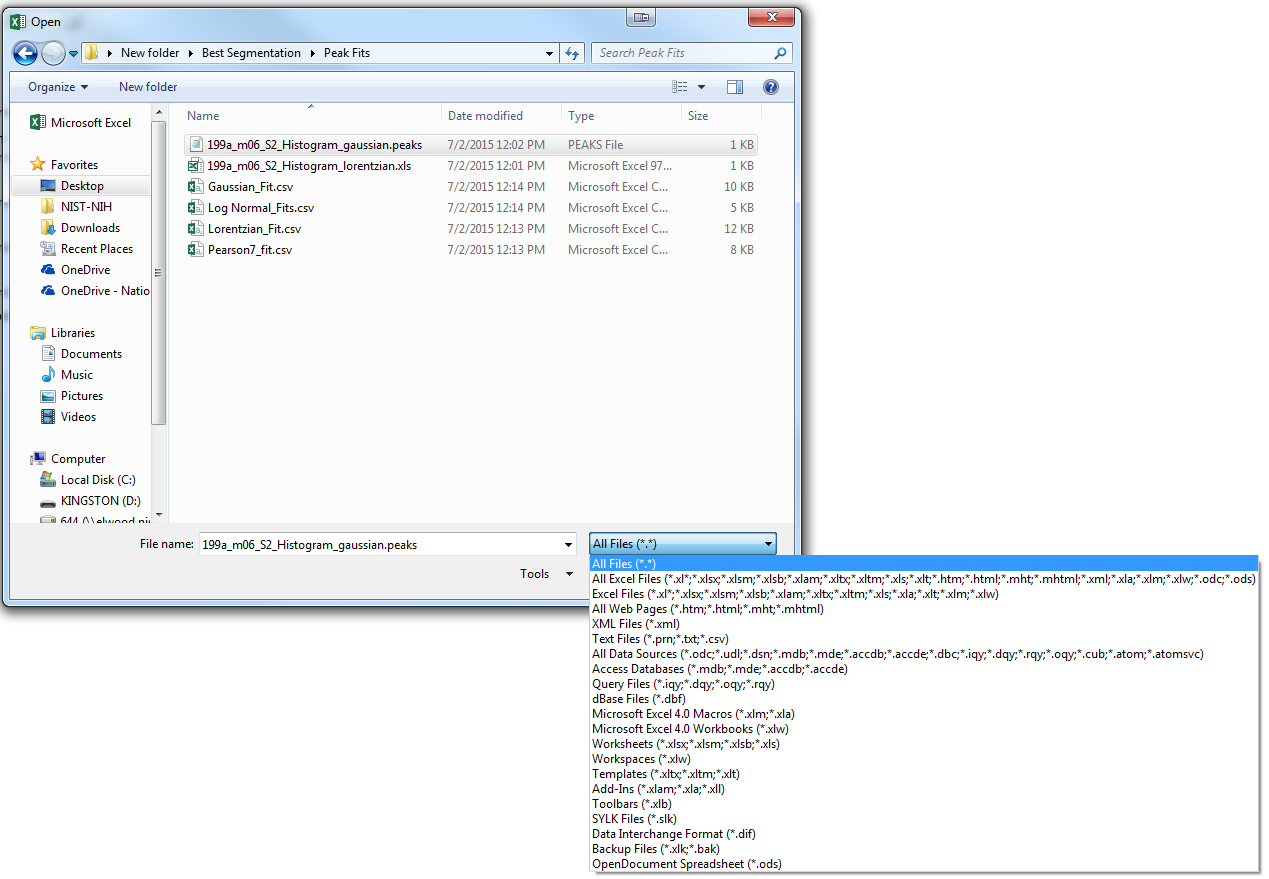


**Figure 15: Excel setting of “All Files” from the drop down menu.**

1. Once open the .peaks file contains information about the peak that was fit including the Peak center, height, area, and FWHM. As well as a column for “Parameters” which are actually just the Height, Center, and HWHM of the peak. Figure 16 shows an example output of the .peaks file.
   1. FWHM stands for Full Width Half Maximum of the peak and represents the width of the Gaussian peak at half its maximum intensity.
   2. HWHM stands for Half Width Half Maximum of the peak and represents half the width of the Gaussian peak at half its maximum intensity.


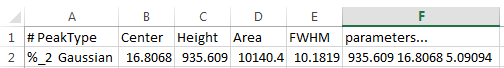


**Figure 16: .peaks file output.**

1. The center of a Gaussian peak is also its mean. The relationship between the FWHM of a Gaussian Peak and the standard deviation of that peak is $StDev = FWHM/(2\sqrt{2ln(2)})$ . Thus, in the .peaks file shown in Figure 16. The Mean fiber radius is 16.8068 pixels and the standard deviation of the fiber radius is $10.1819/(2\sqrt{2ln(2)}) = 4.3239$pixels.
2. If the user has fit multiple peaks to a histogram the .peaks file will contain output for each of the peaks.
3. To convert these radius measures into diameter measurements with actual units, multiply these values by 2 and follow the process covered in the “Manual Segmentation and Pixel to Unit Conversion” training.
   1. For your convenience a text file (Pixel to Unit Conversion.txt) has been included with the images that states the micrometer to pixel correction.
4. Perform the above analysis on each of the 6 images given to you and record your answers in Questions 1-6 from the “Protocol for the Analysis of the Output of DiameterJ - 3/4" form.
