# Supplementary material for: Training to Improve Precision and Accuracy in the Measurement of Fiber Morphology
Source: PLoS One. 2016 Dec 1;11(12):e0167664. doi: 10.1371/journal.pone.0167664 (PMC5132175; doi:10.1371/journal.pone.0167664)
Supplement: S8 File — (DOC) [file pone.0167664.s008.doc]

# **DiameterJ Combined Fiber Diameter Analysis**

## Introduction:

Often replicate images are taken of a single sample to ensure that the images represent the total sample. Peak fitting each of these images is time consuming and then combining the means and standard deviations can be cumbersome. Thus, the authors recommend combining replicate images of the same sample into one total “mesh” analysis. In effect, combining the histograms of images taken at identical magnifications and similar working distances into one cumulative histogram and then fitting only that histogram to get cumulative statistics about your sample.

This is preferred over analysis of individual images because conceptually you are merely stitching your images together into one large sample image and then obtaining a histogram from this image. Thus, distributions are usually found to be more normal (as replicate image number increases) and statistics obtained from the fit of these histogram peaks are stronger statistically (have lower residual error).

Thus, combined histogram peak fitting is highly recommended if you would like to analyze replicate images of a single sample. In the below example a template will be given to you to make the analysis of the combined histograms of multiple images more simple. You will be analyzing the same images from the previous section (199a_m02.tif - 199a_m09.tif). The instructions below will walk through each step of the process and how to use the template.

**Image Analysis**

1. The link below is to a spreadsheet that is set up to automatically tabulate a cumulative histogram.
   1. <https://goo.gl/r8pRdg>
2. Click the link and once loaded, find the triple dot icon in the top right of the sheet. This icon is circled in red in the image below. Download the sheet and paste in your histogram values.
   1. All cells should be locked except the cells that are dark gray.


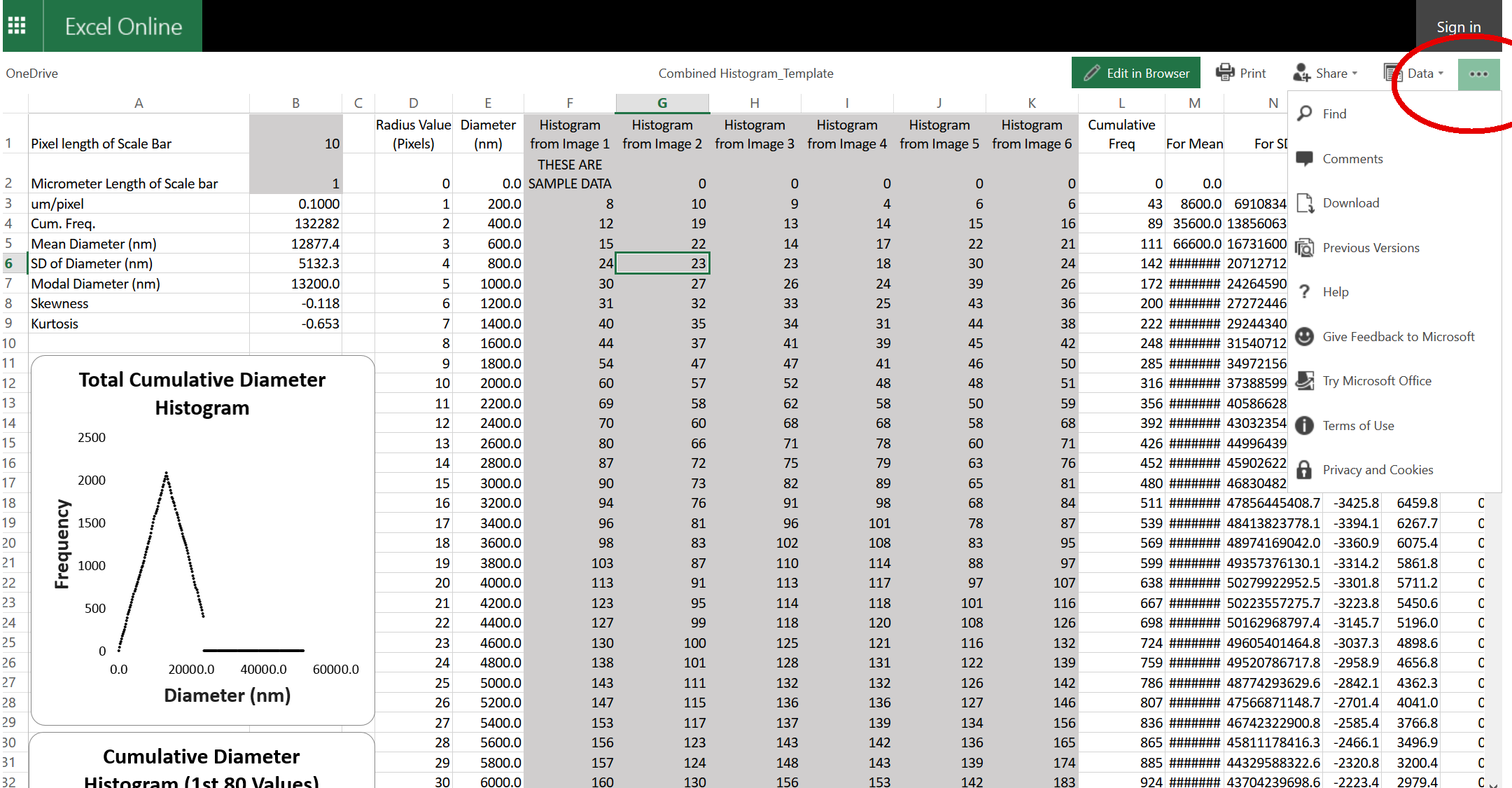


1. If the user prefers they can use the below editable sheet in their own google account. However, it is strongly recommended that the user create an immediate copy of this spreadsheet, in their own google account, and modify that because no changes will be saved in this document after the user closes this document and other users can make changes while the user is editing this document. <https://goo.gl/8BPytk>
2. The spreadsheet is designed to do most of the work for you. It is color coded so that grey cells are cells that the user should edit and the white cells are filled with formuli to calculate various statistics and do not need to be edited by the user.
3. In the template all grey cells have dummy values to show an example of what the sheet will look like once populated by real data. The user should replace this data by copying and pasting all histograms from replicate images into the file. Figure 1 belows shows the template before any data has been pasted in.


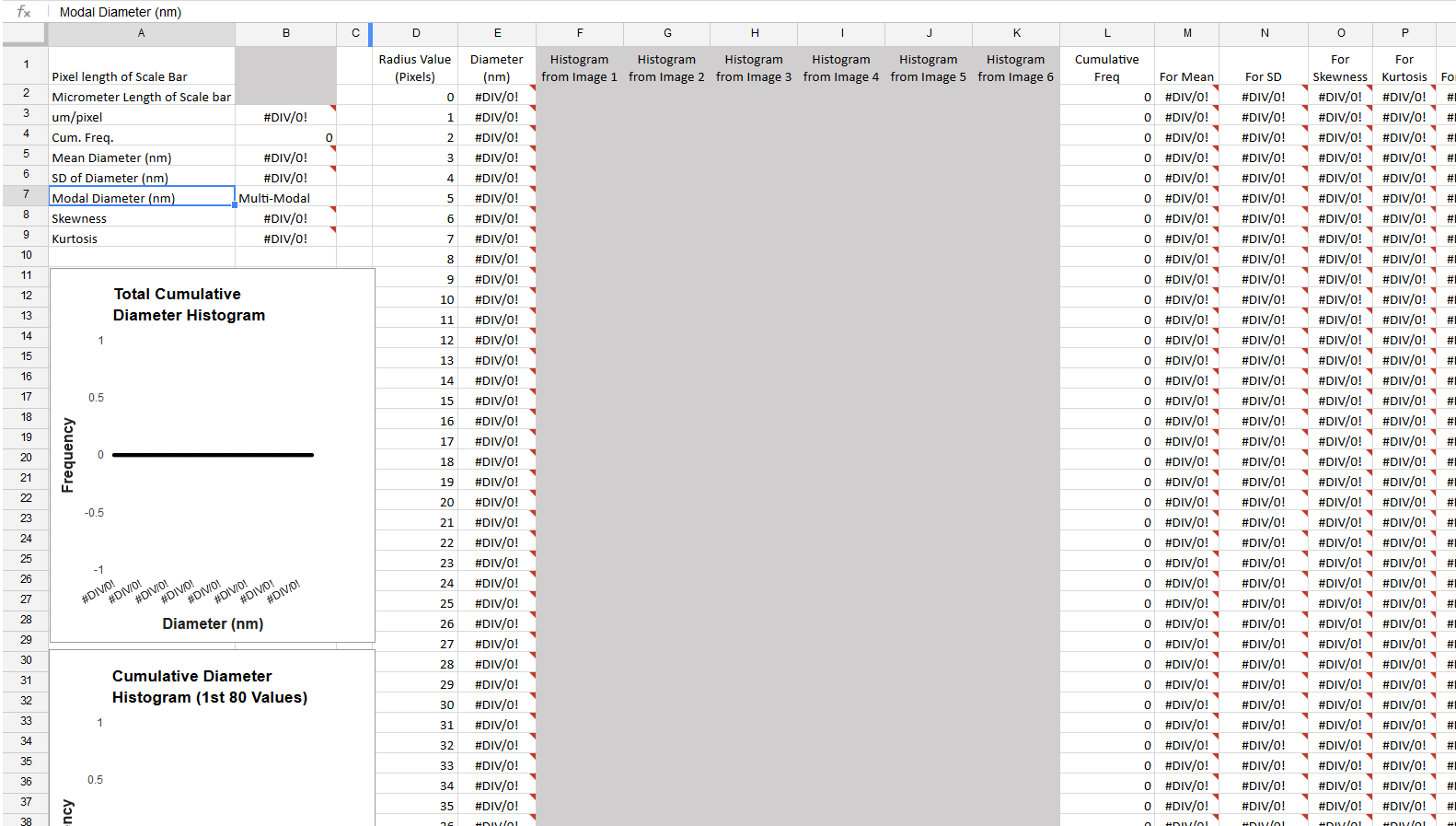


**Figure 1: Template for pixel to unit conversion.**

1. As values are pasted into the spreadsheet the error values (#DIV/0!) will disappear and the graphs will populate themselves.
2. All formulae are shown when clicking on a cell if the user would like to create their own spreadsheet template.

**The remainder of this tutorial assumes that the analysis completed in the “**[**DiameterJ Fiber Metric Analysis**](https://goo.gl/1u1j91)**” document has been completed. If it has not, please go back to this document and complete the analysis outlined there and then return to this tutorial.**

1. The first step, after downloading/saving the spreadsheet is to enter in the Pixel and micrometer length of the scale bar in the images you already downloaded from - <https://goo.gl/Ncin8D>
   1. These images are files 199a_m02.tif - 199a_m09.tif
2. To obtain the scale bar dimensions follow the instructions outlined in the [Manual Segmentation and Pixel to Unit Conversion](https://docs.google.com/document/d/1Hywk3DsRz7tpkafyjncpBxCQTZw5NnqkkWKp4LKguYk/edit?usp=sharing) document.
   1. A text file (Pixel to Unit Conversion.txt) is included with the images that gives the user the correct answer. But we encourage the user to do this manually to confirm that you can still do the conversion correctly.
3. Once the unit conversion has been entered open the “XX_Histogram.csv” files found in the Histogram folder.
4. Copy and paste the “Frequency” column from the “XX_Histogram.csv” files into the empty cell of the template. Be sure to replace the text that says “Frequency from Image X” with the name of the image the frequency is from. Figure 2 shows the first few rows of the template after adding the unit conversion and pasting in the 199a_m02.tif and 199a_m04.tif frequency data.
   1. **All of these frequencies can be pasted into the same file because all of the images were taken at the same magnification and approximately the same working distance. The user CANNOT combine histograms in this manner if images were taken at different magnifications.**


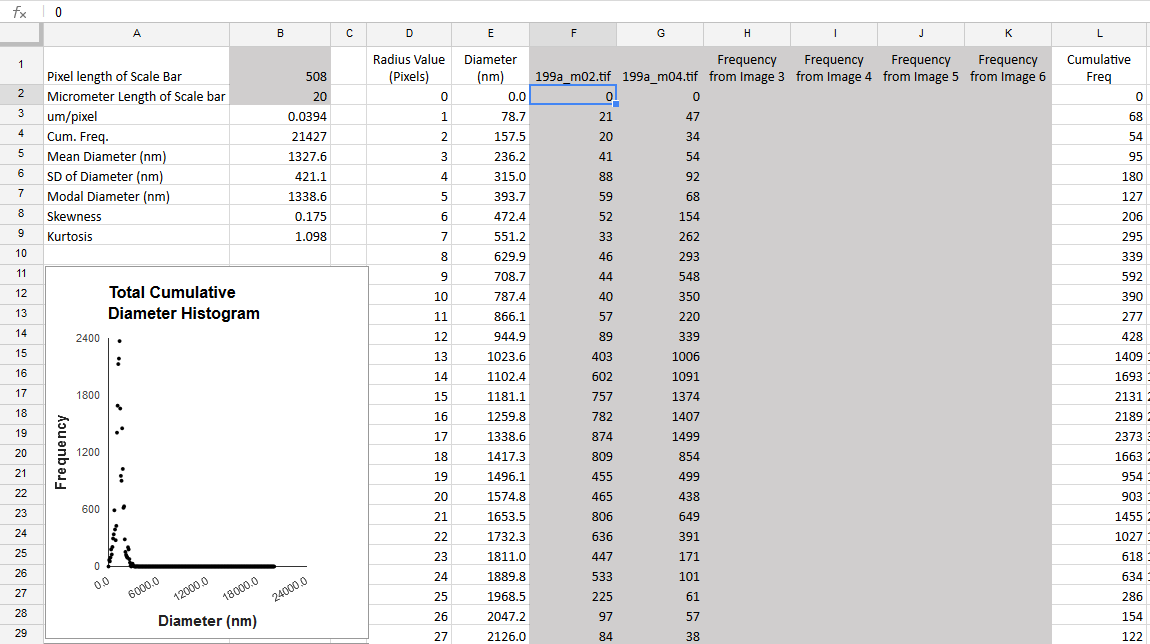


**Figure 2: Template after adding two images.**

1. After pasting in the last frequency values from the images the first thing to check is the cumulative histogram graphs on the left side of the sheet. The user should look at these to see if there is only one peak, and how parametrically distributed that peak appears.
2. If there is only one peak the next values the user should check are the skewness and kurtosis values. If both of these values are between -1 and 1 and there is only one large peak in the histogram than the mean diameter and standard deviation (SD of Diameter (nm)) are correct. If they are not then further peak fitting is needed.
3. If the images that were taken were taken at multiple magnifications or significantly different working distances than the unit conversion of pixels to nanometers or micrometers will not be the same for the two different images.
4. If images HAVE NOT been imaged at multiple magnifications then skip to step 27.
5. If Images have been taken at multiple magnifications see steps 16 through 24 below.
6. Make a copy of the template to paste in the frequencies from the other magnifications.
   1. For example if three images were taken at 1000x and 3 were taken at 5000x then only paste the frequencies from the 1000x images in one template and the 5000x images in the other template.
7. To combine the two histograms open a new spreadsheet, referred to as “Combined Spreadsheet” below. Select the template with the frequencies copied into it from the lower magnification images and copy the “Diameter (nm)” column from this template into Column A of the “Combined Spreadsheet”.
   1. When copying be sure to only paste the values, not the formulas, into the “Combined Spreadsheet”. To do this, before pasting right click the cell you would like to paste into and choose “Paste values only” in Google Sheets or the “123” symbol in Excel. Figure 3 shows the location of these menus and the Excel icon to paste values is circled in red.
   2. The latest version of Google Sheets has trouble pasting “values only” between sheets. While this bug is being fixed the user can use Excel, or any other desktop spreadsheet software, or the user can paste the copied column into a new blank column in the same spreadsheet as “values only” and then copy these cells into the Combined Spreadsheet.


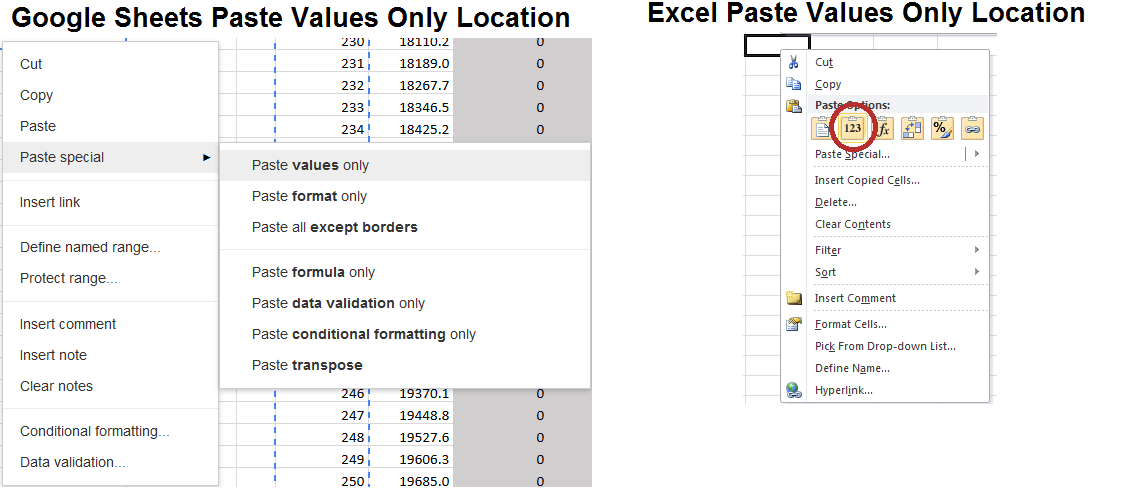


**Figure 3: Paste values only location in Google Sheets and Excel.**

1. Next, copy the “Cumulative Freq” column of the lower magnification template into Column B of the “Combined Spreadsheet”.
   1. When copying be sure to only paste the values, not the formulas, into the sheet.
2. Now, select the template with the frequencies copied into it from the higher magnification images and copy the “Diameter (nm)” column from this template below the values of the lower magnification in Column A of the “Combined Spreadsheet”.
   1. When copying be sure to only paste the values, not the formulas, into the sheet.
3. Copy the “Cumulative Freq” column of the higher magnification template below the values of the lower magnification in Column B of the “Combined Spreadsheet”.
4. The user should now have only two columns of data in the “Combined Spreadsheet”. Column A with the diameter values and Column B with the cumulative frequencies. Figure 4 shows an example of what this should look like.


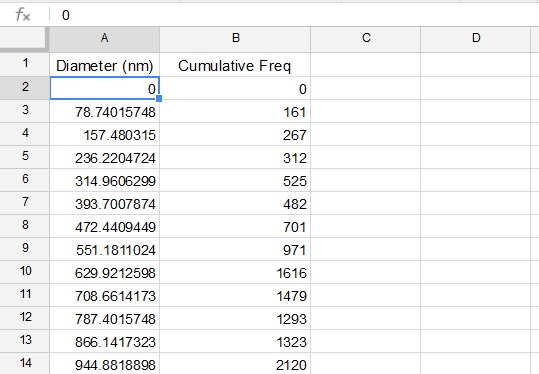


**Figure 4: Combined Spreadsheet layout.**

1. Highlight all the values in both columns and sort them based on column A from lowest to highest. Figure 5 shows where the menu commands to do this are.
   1. This can be done in Google Sheets by choosing Data → Sort Sheet by Column A, A → Z
   2. This can be done in Excel by going to Sort & Filter Icon on the top right of the Home menu and selecting “Sort Smallest to Largest”.


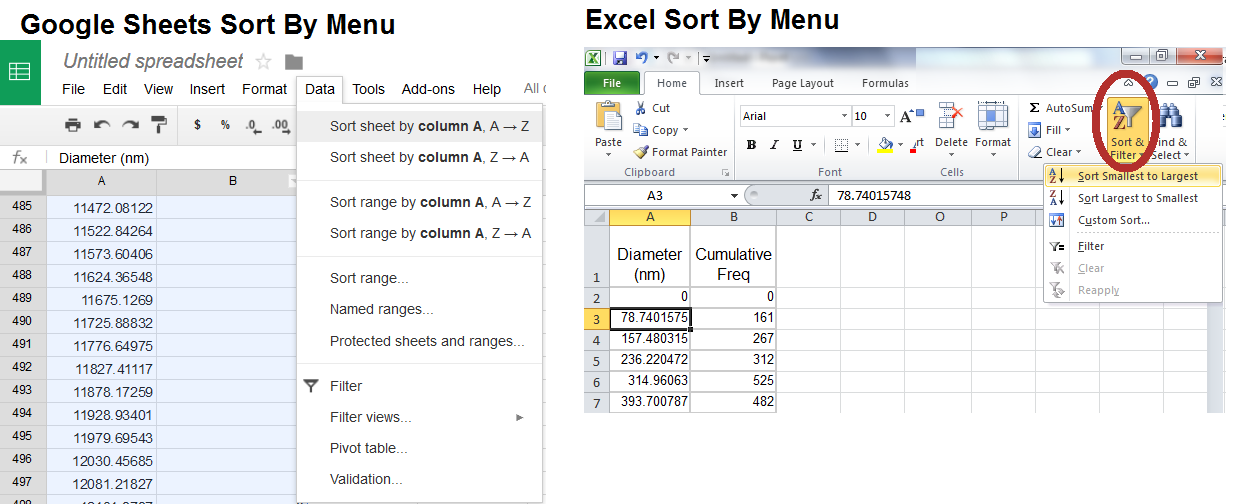


**Figure 5: Google Sheets and Excel sort by menus.**

1. Once sorted, remove the text from the bottom two lines of the data.
2. Save the “Combined Sheet” as a .csv file.
3. If you were analyzing images at multiple magnifications and completed steps 16-24 above please skip to step 29 below.
4. Open a new spreadsheet, referred to as “Combined Spreadsheet” below. Copy the “Diameter (nm)” column from the template into Column A of the “Combined Spreadsheet”.
   1. When copying be sure to only paste the values, not the formulas, into the “Combined Spreadsheet”. To do this, before pasting right click the cell you would like to paste into and choose “Paste values only” in Google Sheets or the “123” symbol in Excel. Figure 6 shows the location of these menus and the Excel icon to paste values is circled in red.
   2. The latest version of Google Sheets has trouble pasting “values only” between sheets. While this bug is being fixed the user can use Excel, or any other desktop spreadsheet software, or the user can paste the copied column into a new blank column in the same spreadsheet as “values only” and then copy these cells into the Combined Spreadsheet.


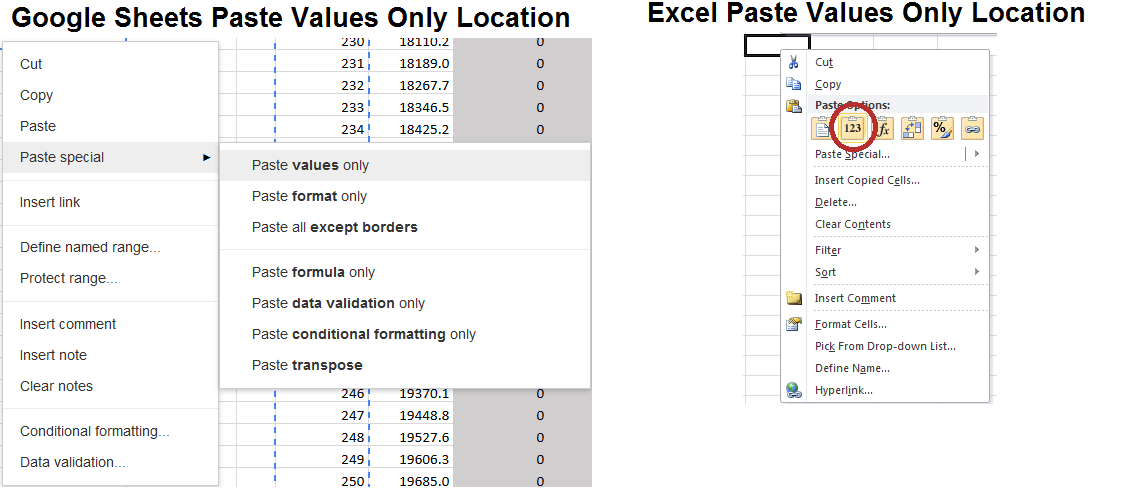


**Figure 6: Paste values only location in Google Sheets and Excel.**

1. Next, copy the “Cumulative Freq” column of the template into Column B of the “Combined Spreadsheet”.
   1. When copying be sure to only paste the values, not the formulas, into the sheet.
2. Save the “Combined Spreadsheet” as a .csv file.
3. Once the “Combined Spreadsheet” is saved open the “Combined Sheet” with Fityk and fit the histogram using the exact same procedure outlined in the “[Fiber Metric Analysis](https://goo.gl/1u1j91)” training.
4. The skewness, kurtosis and mean and standard deviation of fiber diameter obtained from the worksheet and the Fityk fits of the cumulative histogram will then be used to answer questions 7 and 8 of the training.
