# Supplementary material for: Training to Improve Precision and Accuracy in the Measurement of Fiber Morphology
Source: PLoS One. 2016 Dec 1;11(12):e0167664. doi: 10.1371/journal.pone.0167664 (PMC5132175; doi:10.1371/journal.pone.0167664)
Supplement: S9 File — (DOCX) [file pone.0167664.s009.docx]

# **DiameterJ Fiber Metric Analysis**

## Introduction:

Up to this point, this training has focused on quantification and analysis of fiber diameter. However, DiameterJ produces many other metrics and outputs for a total analysis of fiber mat morphologies. These metrics are collectively referred to as “Image Dependent Metrics” because they have been found to change drastically depending on how the image was taken, which segmentation algorithm is used, and the focal depth of the image. It has been found that these metrics can vary two to three times their range when analyzing the same image if different contrast settings are used in the SEM when taking the image or in the selection of segmentation algorithm. However, these metrics can serve as relative measures between samples that were imaged under identical microscope settings and analyzed with identical segmentation algorithms. Additionally, if enough images were collected to generate a representative area of the total sample these metrics can provide insight into the composition of the fiber scaffolds. Work is underway to determine how these metrics compare to 3 dimensional analysis of fiber scaffolds, however at this time no firm conclusions can be drawn between 2-D SEM images and 3-D scaffold structures. Finally, fiber diameter has been shown to be relatively insensitive to the above variances, especially once the user has been trained in “proper” segmentation selection

For the below training you will be analyzing the same images from the previous section ([199a_m02.tif - 199a_m09.tif](https://drive.google.com/open?id=0B2P6KQtDoo2NbEhzQlplYTRueTA)). The instructions below will walk through each step of the process to analyze the image dependent metrics of these images.

**Image Analysis**

1. Pore information is often compared in the literature and correlated to mechanical properties, nutrient diffusion, and scaffold morphology. However, determining pore size from 2D images, when “pores” are actually large interconnected void spaces produced by fibers overlapping, is extremely challenging because it difficult to produce metrics that are representative of the entire sample. Additionally, the definition of “pores” for nanofiber mats must be explicitly defined due to the nebulous nature of the structure of these void spaces. Thus, the pore space referenced to in this document refers to the area of the image which is not segmented as fiber. In Figure 1 below all of the space that is black is counted as pore space.


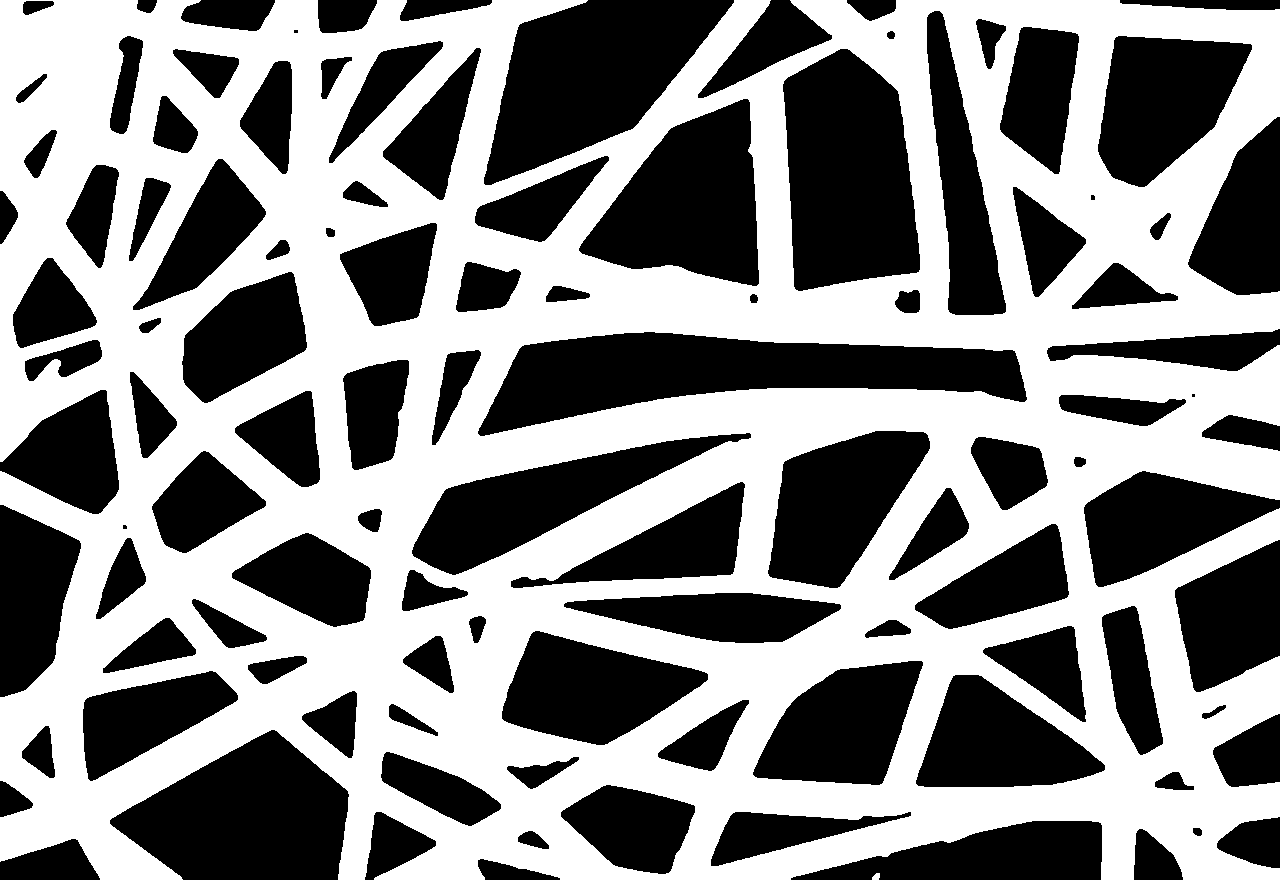


**Figure 1: Example of pore area. All black space in this image is counted as pore area.**

1. Pore area, and any other image dependent metric (percent porosity, pore size, characteristic length, intersection density, and number of pores) can only be compared and/or combined with other images if all images were taken at the same magnification, working distance, microscope settings (voltage, amerpage, brightness, contrast, etc.), and post processing (segmentation algorithm and image clean-up technique). If this is not the case then images cannot be compared.
2. For all images in this training and in subsequent tests assume that all microscope settings were identical for each image and that the same microscope segmentation algorithm was chosen.
3. To find the mean pore area of an image users can go to either the “XX_Total Summary.csv” file in the “Summaries folder or the “XX_Pore Data.csv” in the “Histograms” folder.
   1. The mean pore area can be found in the “XX_Total Summary.csv” file next to the variable “Mean Pore Area”
   2. The mean pore area can be found in the “XX_Pore data.csv” file in the area column next to the value “Mean” in the label column.
4. Figure 2 shows the location of the mean pore area value in both files.


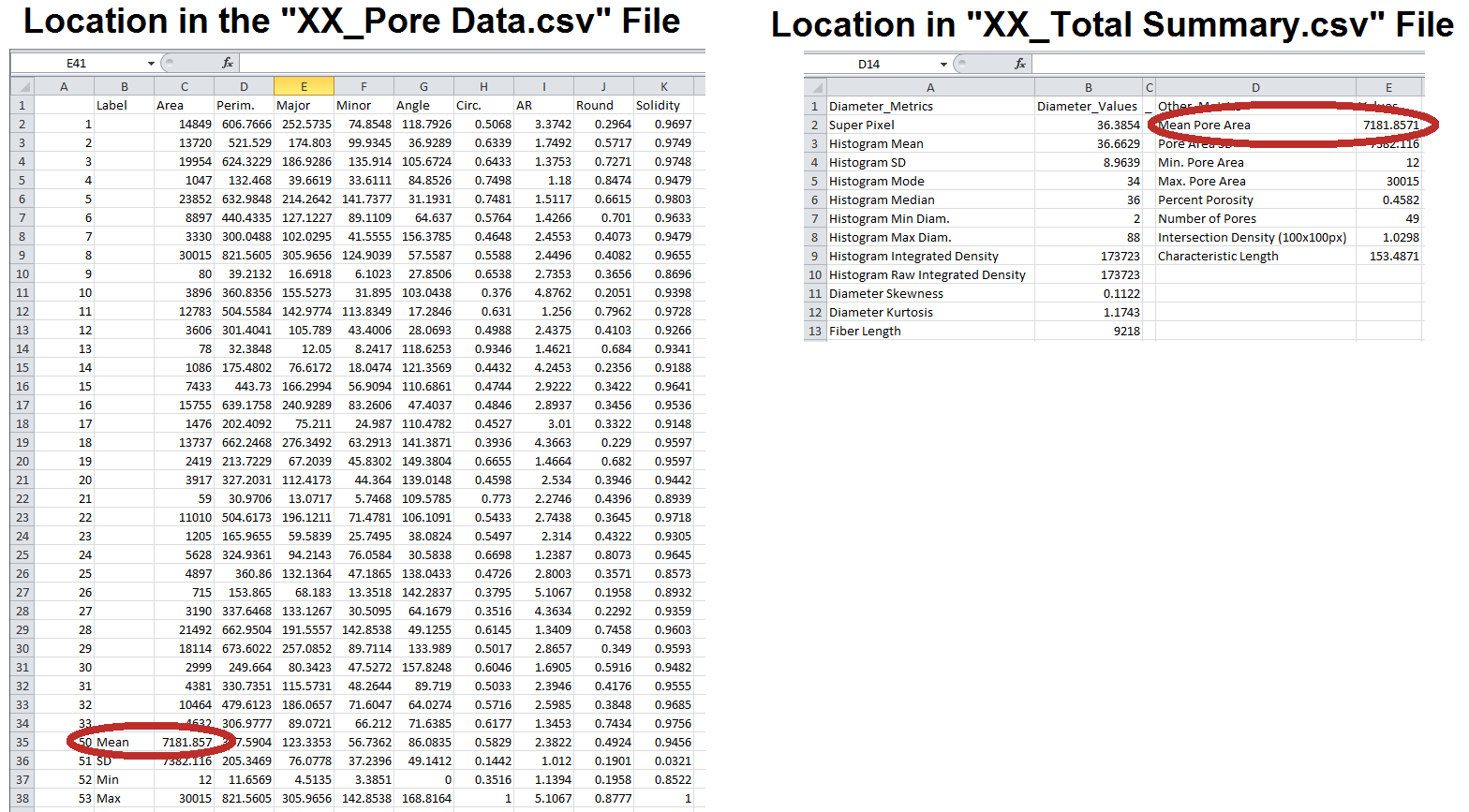


**Figure 2: Mean pore area metric location.**

1. To find the mean pore area standard deviation of an image users can go to either the “XX_Total Summary.csv” file in the “Summaries folder or the “XX_Pore Data.csv” in the “Histograms” folder.
   1. The mean pore area standard deviation can be found in the “XX_Total Summary.csv” file next to the variable “Pore Area SD”
   2. The mean pore area standard deviation can be found in the “XX_Pore data.csv” file in the area column next to the value “SD” in the label column.
2. Figure 3 shows the location of the mean pore area standard deviation value in both files.


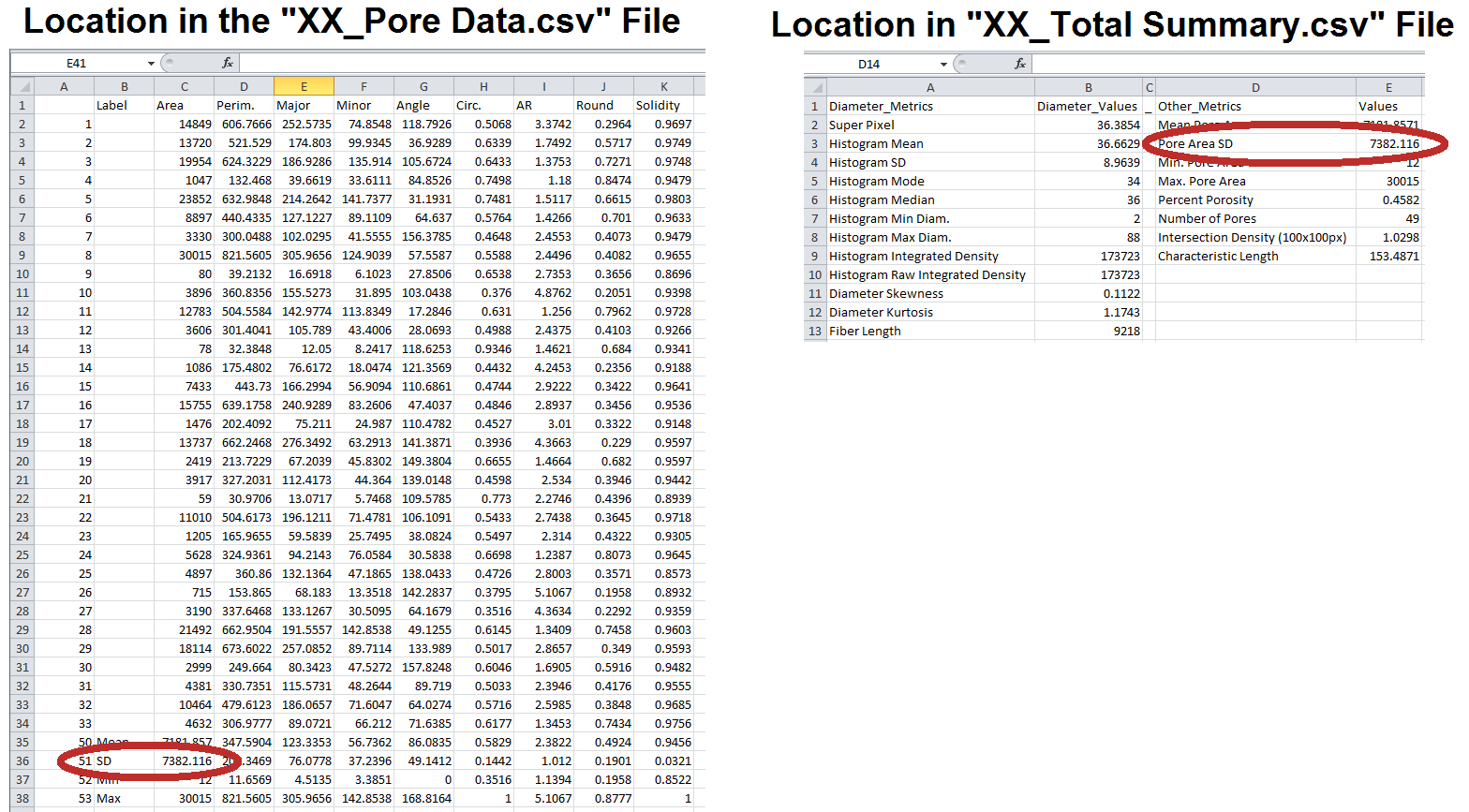


**Figure 3: Mean pore area standard deviation location.**

1. However, if the user wants to combine replicate images from a single sample, as was done in the previous training, taking the average of averages and combining the variance can lead to skewed results.
2. Thus, it is recommended that the user copy and paste all of the areas of the pores from each replicate image to a single sheet and take the mean and standard deviation of this total population rather than taking the average of averages and combining variances.
3. For characteristic length an identical procedure is recommended. Go to the “XX_Intersection.csv” file in the Histograms folder and copy and paste all of the characterisitc lengths of the fibers from each replicate image to a single sheet and take the mean and standard deviation of this total population rather than taking the average of averages and combining variances.
4. Other metrics that can be of value to the user are percent porosity, number of pores, and intersection density.
5. Percent porosity is written as a decimal thus, if percent porosity says 0.4582 it means that 45.82% of the image is pore space.
6. Because none of the metrics mentioned in step 10 above have standard deviations, they are cumulative statistics, to get an average and standard deviation from replicate images simply take the average and standard deviation as normal.

The last hurdle to overcome is in unit conversion for both area metrics and for the intersection density.

1. All area metrics (mean pore area, pore area SD, min. pore area, and max. pore area) are given in pixels. In these metrics each pixel takes up an area that is the square of the unit conversion.
   1. For example if your scale bar was 500 pixels long and represented 5 micrometers. 5 micrometers/500 pixels = 0.01 micrometers/pixel. Thus, the area that the pixel represents equals 0.01 micrometers/pixel tall and 0.01 micrometers/pixel wide.
      1. In other words pixel area = 0.01*0.01 = 0.0001 micrometers^2.
   2. Thus, if you had a pore area of 5000 pixels the area in micrometers^2 would be 5000*0.0001 = 0.5 micrometers^2
   3. Another example: if you scale bar was 254 pixels long and represented 10 micrometers. What would the area of a 6543 pixel pore be?
      1. Answer: 10.14 micrometers^2
2. Intersection density is given as number of fiber intersections found in a 100 px x 100 px area. Thus, to convert this factor into intersections per micrometer^2 multiply the unit conversion by 100 and square this term. Then divide the number DiameterJ produces by this new value and you get the intersections/micrometer^2.
   1. For example: if your scale bar was 400 pixels long and represented 5 micrometers. 5 micrometers/400 pixels = 0.0125 micrometers/pixel. Thus, 100 pixels equals 0.0125 * 100 = 1.25 micrometers. 1.25 micrometers* 1.25 micrometers = 1.5625 micrometers^2. Now if the original intersection density was 2 intersections/(100px x 100px) area you need only divide 2/1.5625 = 1.28 intersections/micrometer^2.
      1. In other words intersection density = 2/(.0125*.0125*10000) intersections/micrometer^2.
   2. Another example: DiameterJ reports an intersection density of 1.5526. If your scale bar was 405 pixels long and represented 20 micrometers. What is your number of intersections per micrometer^2?
      1. Answer: 0.0637 intersections/micrometer^2

The user may note that a considerable amount of the trainings they have completed were about the diameter and a relatively small amount of time was devoted to analyzing other metrics. That is because these metrics are “image dependent” and thus change drastically based off of how the image is segmented and by extension the imaging conditions. It has been found that this is not the case for diameter as long as “good” segmentations have been selected.

Once you have completed the quiz on this training you will be asked to analyze a final set of images. Once you have entered the values from this analysis into the online form you will be done!
