# Supplementary material for: Training to Improve Precision and Accuracy in the Measurement of Fiber Morphology
Source: PLoS One. 2016 Dec 1;11(12):e0167664. doi: 10.1371/journal.pone.0167664 (PMC5132175; doi:10.1371/journal.pone.0167664)
Supplement: S1 Table — Probability values below the significance level (0.05) are highlighted in grey. For normality tests, a P value <0.05 indicates non-normal. Also, skewness and kurtosis values not between -1 and 1 were highlighted as these values generally indicated distributions that are non-normal. All other P values less than 0.05 indicate a rejection of the null hypothesis which was that means or variances were equal. (PPTX) [file pone.0167664.s010.pptx]

## Slide 1
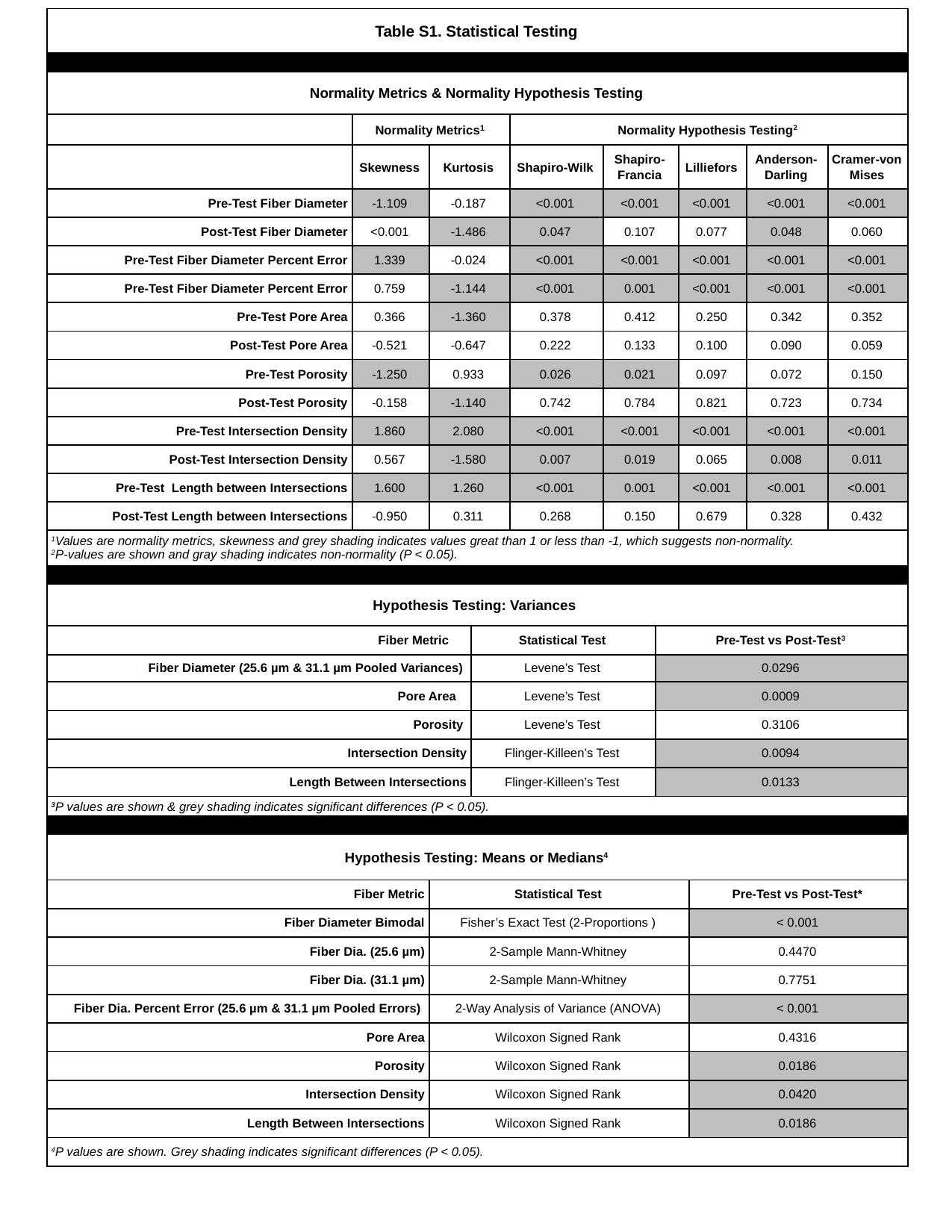

| Table S1. Statistical Testing | | | | | | | | | | |
| --- | --- | --- | --- | --- | --- | --- | --- | --- | --- | --- |
| | | | | | | | | | | |
| Normality Metrics & Normality Hypothesis Testing | | | | | | | | | | |
| | Normality Metrics1 | | | Normality Hypothesis Testing2 | | | | | | |
| | Skewness | Kurtosis | | Shapiro-Wilk | Shapiro-Francia | | Lilliefors | | Anderson-Darling | Cramer-von Mises |
| Pre-Test Fiber Diameter | -1.109 | -0.187 | | <0.001 | <0.001 | | <0.001 | | <0.001 | <0.001 |
| Post-Test Fiber Diameter | <0.001 | -1.486 | | 0.047 | 0.107 | | 0.077 | | 0.048 | 0.060 |
| Pre-Test Fiber Diameter Percent Error | 1.339 | -0.024 | | <0.001 | <0.001 | | <0.001 | | <0.001 | <0.001 |
| Pre-Test Fiber Diameter Percent Error | 0.759 | -1.144 | | <0.001 | 0.001 | | <0.001 | | <0.001 | <0.001 |
| Pre-Test Pore Area | 0.366 | -1.360 | | 0.378 | 0.412 | | 0.250 | | 0.342 | 0.352 |
| Post-Test Pore Area | -0.521 | -0.647 | | 0.222 | 0.133 | | 0.100 | | 0.090 | 0.059 |
| Pre-Test Porosity | -1.250 | 0.933 | | 0.026 | 0.021 | | 0.097 | | 0.072 | 0.150 |
| Post-Test Porosity | -0.158 | -1.140 | | 0.742 | 0.784 | | 0.821 | | 0.723 | 0.734 |
| Pre-Test Intersection Density | 1.860 | 2.080 | | <0.001 | <0.001 | | <0.001 | | <0.001 | <0.001 |
| Post-Test Intersection Density | 0.567 | -1.580 | | 0.007 | 0.019 | | 0.065 | | 0.008 | 0.011 |
| Pre-Test Length between Intersections | 1.600 | 1.260 | | <0.001 | 0.001 | | <0.001 | | <0.001 | <0.001 |
| Post-Test Length between Intersections | -0.950 | 0.311 | | 0.268 | 0.150 | | 0.679 | | 0.328 | 0.432 |
| 1Values are normality metrics, skewness and grey shading indicates values great than 1 or less than -1, which suggests non-normality. 2P-values are shown and gray shading indicates non-normality (P < 0.05). | | | | | | | | | | |
| | | | | | | | | | | |
| Hypothesis Testing: Variances | | | | | | | | | | |
| Fiber Metric | | | Statistical Test | | | Pre-Test vs Post-Test3 | | | | |
| Fiber Diameter (25.6 µm & 31.1 µm Pooled Variances) | | | Levene’s Test | | | 0.0296 | | | | |
| Pore Area | | | Levene’s Test | | | 0.0009 | | | | |
| Porosity | | | Levene’s Test | | | 0.3106 | | | | |
| Intersection Density | | | Flinger-Killeen’s Test | | | 0.0094 | | | | |
| Length Between Intersections | | | Flinger-Killeen’s Test | | | 0.0133 | | | | |
| 3P values are shown & grey shading indicates significant differences (P < 0.05). | | | | | | | | | | |
| | | | | | | | | | | |
| Hypothesis Testing: Means or Medians4 | | | | | | | | | | |
| Fiber Metric | | Statistical Test | | | | | | Pre-Test vs Post-Test\* | | |
| Fiber Diameter Bimodal | | Fisher’s Exact Test (2-Proportions ) | | | | | | < 0.001 | | |
| Fiber Dia. (25.6 µm) | | 2-Sample Mann-Whitney | | | | | | 0.4470 | | |
| Fiber Dia. (31.1 µm) | | 2-Sample Mann-Whitney | | | | | | 0.7751 | | |
| Fiber Dia. Percent Error (25.6 µm & 31.1 µm Pooled Errors) | | 2-Way Analysis of Variance (ANOVA) | | | | | | < 0.001 | | |
| Pore Area | | Wilcoxon Signed Rank | | | | | | 0.4316 | | |
| Porosity | | Wilcoxon Signed Rank | | | | | | 0.0186 | | |
| Intersection Density | | Wilcoxon Signed Rank | | | | | | 0.0420 | | |
| Length Between Intersections | | Wilcoxon Signed Rank | | | | | | 0.0186 | | |
| 4P values are shown. Grey shading indicates significant differences (P < 0.05). | | | | | | | | | | |
